# Burden of disease in francophone Africa, 1990–2017: a systematic analysis for the Global Burden of Disease Study 2017

**DOI:** 10.1016/S2214-109X(20)30024-3

**Published:** 2020-02-19

**Authors:** Charbel El Bcheraoui, Honoré Mimche, Yodé Miangotar, Varsha Sarah Krish, Faye Ziegeweid, Kris J Krohn, Martin Herbas Ekat, Jobert Richie Nansseu, Zacharie Tsala Dimbuene, Helen Elizabeth Olsen, Roger C K Tine, Christopher M Odell, Christopher E Troeger, Nicholas J Kassebaum, Tamer Farag, Simon I Hay, Ali H Mokdad

**Affiliations:** a Institute for Health Metrics and Evaluation, University of Washington, Seattle, WA, USA; b Department of Health Metrics Sciences, School of Medicine, University of Washington, Seattle, WA, USA; c Department of Anesthesiology & Pain Medicine, University of Washington, Seattle, WA, USA; d Evidence-Based Public Health, Centre for International Health Protection, Robert Koch Institute, Berlin, Germany; e Institut de Formation et de Recherche Démographiques, Université de Yaoundé II, Yaoundé, Cameroon; f Faculté des Sciences Humaines et Sociales, Université de N'Djaména, N'Djaména, Chad; g National AIDS Control Program, Ministry of Health and Population, Brazzaville, Congo; h Department for the Control of Disease, Epidemics and Pandemics, Ministry of Public Health, Yaoundé, Cameroon; i Department of Public Heath, Faculty of Medicine and Biomedical Sciences, Université de Yaoundé I, Yaoundé, Cameroon; j Department of Population Sciences and Development, Faculty of Economics and Management, University of Kinshasa, Kinshasa, Democratic Republic of the Congo; k Microdata Access Division, Statistics Canada, Ottawa, ON, Canada; l Global Health, Bill & Melinda Gates Foundation, Seattle, WA, USA; m Service de Parasitologie, Faculté de Médecine, de Pharmacie et d'Odontologie, Université Cheikh Anta Diop, Dakar, Senegal

## Abstract

**Background:**

Peer-reviewed literature on health is almost exclusively published in English, limiting the uptake of research for decision making in francophone African countries. We used results from the Global Burden of Diseases, Injuries, and Risk Factors Study (GBD) 2017 to assess the burden of disease in francophone Africa and inform health professionals and their partners in the region.

**Methods:**

We assessed the burden of disease in the 21 francophone African countries and compared the results with those for their non-francophone counterparts in three economic communities: the Economic Community of West African States, the Economic Community of Central African States, and the Southern African Development Community. GBD 2017 employed a variety of statistical models to determine the number of deaths from each cause, through the Cause of Death Ensemble model algorithm, using CoDCorrect to ensure that the number of deaths per cause did not exceed the total number of estimated deaths. After producing estimates for the number of deaths from each of the 282 fatal outcomes included in the GBD 2017 list of causes, the years of life lost (YLLs) due to premature death were calculated. Years lived with disability (YLDs) were estimated as the product of prevalence and a disability weight for all mutually exclusive sequelae. Disability-adjusted life-years (DALYs) were calculated as the sum of YLLs and YLDs. All calculations are presented with 95% uncertainty intervals (UIs). A sample of 1000 draws was taken from the posterior distribution of each estimation step; aggregation of uncertainty across age, sex, and location was done on each draw, assuming independence of uncertainty. The lower and upper UIs represent the ordinal 25th and 975th draws of each quantity and attempt to describe modelling as well as sampling error.

**Findings:**

In 2017, 779 deaths (95% UI 750–809) per 100 000 population occurred in francophone Africa, a decrease of 45·3% since 1990. Malaria, lower respiratory infections, neonatal disorders, diarrhoeal diseases, and tuberculosis were the top five Level 3 causes of death. These five causes were found among the six leading causes of death in most francophone countries. In 2017, francophone Africa experienced 53 570 DALYs (50 164–57 361) per 100 000 population, distributed between 43 708 YLLs (41 673–45 742) and 9862 YLDs (7331–12 749) per 100 000 population. In 2017, YLLs constituted the majority of DALYs in the 21 countries of francophone Africa. Age-specific and cause-specific mortality and population ageing were responsible for most of the reductions in disease burden, whereas population growth was responsible for most of the increases.

**Interpretation:**

Francophone Africa still carries a high burden of communicable and neonatal diseases, probably due to the weakness of health-care systems and services, as evidenced by the almost complete attribution of DALYs to YLLs. To cope with this burden of disease, francophone Africa should define its priorities and invest more resources in health-system strengthening and in the quality and quantity of health-care services, especially in rural and remote areas. The region could also be prioritised in terms of technical and financial assistance focused on achieving these goals, as much as on demographic investments including education and family planning.

**Funding:**

Bill & Melinda Gates Foundation.

## Introduction

**Figure 1:**
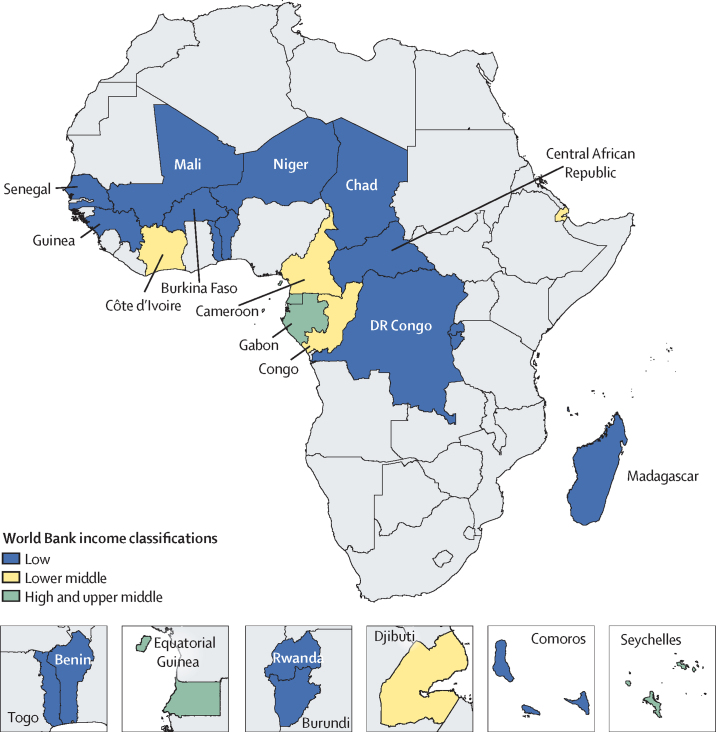
Francophone African countries classified by World Bank income level

Francophone Africa is composed of 21 countries where French is an official language, and is divided between the west, central, and eastern parts of Africa (figure 1).^1^ While populations of other countries in Africa might speak French, such as Algeria, Tunisia, or Morocco, French is not an official language in these countries.^2^ With this definition, francophone Africa includes Benin, Burkina Faso, Burundi, Cameroon, the Central African Republic, Chad, Comoros, Congo (Brazzaville), Côte d'Ivoire, the Democratic Republic of the Congo, Djibouti, Equatorial Guinea, Gabon, Guinea, Madagascar, Mali, Niger, Rwanda, Senegal, the Seychelles, and Togo. This region is home to more than 324 334 000 inhabitants, representing 25·2% of the African population.^3^

**Research in context**

**Evidence before this study**

While previous iterations of the Global Burden of Diseases, Injuries, and Risk Factors Study (GBD), including GBD 2016, provided estimates for diseases and disability in 195 countries and territories, they did not provide estimates on the burden of disease in francophone Africa as a region. Evidence from the area has been limited to epidemiological reports from ministries of health and periodic surveys. Importantly, the peer-reviewed literature on health in the region, including GBD analyses, is almost exclusively published in English, limiting the uptake of research for decision making in African countries where French is an official language.

**Added value of this study**

This study provides a complete picture of health and health trends since 1990 in all francophone African countries, including publication in a language (French) that is accessible to those countries. The study highlights the delay of the francophone African region along the epidemiological transition compared with other regions of Africa, as well as the disparities between francophone and non-francophone countries within three major African economic communities. The findings from this study will allow scientists and decision makers from francophone Africa to use the best available evidence for local health programming.

**Implications of all the available evidence**

This analysis gives francophone African countries access to the most current and complete estimates of disease burden in terms of years of life lost, years lived with disability, disability-adjusted life-years, and life expectancy, and the changes in disease burden since 1990. The results highlight the most urgent needs for financial assistance in the region, with a particular focus on strengthening health systems.

Within the region, francophone African countries are part of different economic groups that promote development collaborations. The largest of these are the Economic Community of West African States (15 countries, nine of which are francophone), the Economic Community of Central African States (11 countries, nine francophone), and the Southern African Development Community (16 countries, four francophone), with the Democratic Republic of the Congo a member of the last two groups (appendix 2 p 3). However, within each of these communities, francophone countries show weaker development indicators that can adversely affect health. For instance, in terms of the Socio-demographic Index (SDI)—a composite measure of income per capita, mean years of education over the age of 15 years, and fertility rate under the age of 25 years—francophone countries in these three economic groups average 0·33, 044, and 0·48 compared with 0·45, 0·48, and 0·52 for their non-francophone counterparts, respectively.^4^

Epidemiological transition is highly driven by social and economic development, including that of the health system.^5^ Hence, with their sociodemographic disadvantages and weaker health systems, francophone African countries might be at a different stage of their epidemiological transition than their non-francophone counterparts and facing different health patterns and challenges to the rest of the region. As non-communicable diseases are increasing in prevalence in Africa, infectious diseases, nutritional deficiencies, and complications of pregnancy and childbirth are still prevalent.^4^

The burden of disease is the cornerstone of decision making in population health.^6^ Health decision makers use it to describe and attempt to understand health issues at the population level, with a view to prioritising interventions. Thus, the timely availability of reliable data is an essential part of health decision making. However, with the quasi-domination of the scientific literature, and particularly peer-reviewed literature, by the English language, the scientific community in francophone Africa, including health decision makers, is isolated from the contribution to and the use of this literature.^7^, ^8^ In general, very few writings on the health of populations in these countries are available in an accessible language. For example, of the 246 peer-reviewed articles published from 1990 to 2015 that originated in west Africa, only 37 were in French.^9^ In francophone African countries, the weakness of scientific production on population health and health systems is linked to the small number of health research structures, the lack of resources allocated to universities, and the absence or inadequacy of health information systems.^10^

The Global Burden of Diseases, Injuries, and Risk Factors Study (GBD) has been published in English since 1990 and, since 2015, the study's data visualisation tool has been published in Chinese, Spanish, Japanese, Norwegian, Portuguese, Russian, and Swedish, allowing a wider audience to access the study's results, thus increasing its usefulness around the world.^11^, ^12^ Unfortunately, no GBD publications have been in French—in particular, those regarding countries where French is the main language, forcing the local health professionals to rely on English-language publications when English is sometimes not commonly used. We used GBD results to assess the burden of disease in francophone Africa to inform health professionals and their partners in the region, and to highlight the health disparities experienced by these countries within their networks of economic communities. This Article is also being published in French to ensure accessibility to the francophone community worldwide.

## Methods

### Overview

Before the GBD project was initiated in 1991, no comprehensive assessments of human health at a global level had been done. GBD is a global comparative risk assessment exercise, with the first preliminary results (for base year 1990) published in the World Development Report 1993.^13^ This international collaborative effort is currently led by the Institute for Health Metrics and Evaluation in Seattle, WA, USA.

GBD uses methodologies for correcting the under-reporting of deaths and those assigned garbage codes. These codes are misclassifications of deaths present in the data. Some of these codes represent cases where the indicated cause cannot logically have caused the death, such as abdominal stiffness, senility, and yellow nail syndrome. Correction of the codes uses evidence from the medical literature, expert opinion, and statistical techniques to reassign each item to the most probable cause of death.^13^

In this study, we consider the 21 francophone African countries and how they differ from their non-francophone counterparts in three economic communities (appendix 2 p 3). We assess the evolution in the main causes of death in francophone Africa, compared with their evolution in their non-francophone counterparts, and rank the main causes of death in each of the francophone African countries. We assess burden of disease with years of life lost (YLLs) due to premature death, years lived with disability (YLDs), and disability-adjusted life-years (DALYs). We present the evolution of the main causes of YLLs, the total DALYs by sex and country, the expected burden based on SDI, the main risk factors contributing to DALYs, the drivers of change in burden of disease in francophone Africa, and a comparison of the disease burden between francophone and non-francophone countries within the three economic communities.

Information about the data sources, estimation methods, computational tools, and statistical analysis used in the derivation of GBD estimates are available elsewhere.^14^ Data sources used for the GBD analysis in francophone Africa are listed in appendix 3. All data sources used in GBD are evaluated before being included in the analysis. A detailed description of our data sources, their limitations, and their use is published elsewhere.^15^

All GBD research is done on a public-domain secondary database, without nominal identification, in accordance with US Decree number 7724 of May 16, 2012, and Resolution number 510 of April 7, 2016; thus, there was no need to submit this study to a research ethics committee as no ethics approval was required. This analysis complies with the Guidelines for Accurate and Transparent Health Estimates Reporting.^16^

### Metrics and causes disaggregation

After addressing data-quality issues, GBD 2017 employed a variety of statistical models to determine the number of deaths from each cause, through the Cause of Death Ensemble model algorithm.^17^ To ensure that the number of deaths per cause did not exceed the total number of estimated deaths, a correction technique called CoDCorrect was used. This technique ensures that estimates of the number of deaths from each cause do not total more than 100% of deaths in a given year, age group, and sex strata.^18^ After producing estimates for the number of deaths from each of the 282 fatal outcomes included in the list of causes in the GBD 2017 study, YLLs were calculated. For every death due to a particular cause, the number of years lost was estimated on the basis of the highest life expectancy in the deceased individual's age group.^19^, ^20^ YLDs were estimated as the product of prevalence and a disability weight for all mutually exclusive sequelae, corrected for comorbidity and aggregated to cause level.^15^ DALYs were calculated as the sum of YLLs and YLDs. GBD used a list of causes that placed 282 causes of death within a four-level hierarchy (appendix 2 pp 4–13). Level 1 divided causes into three groups: communicable, maternal, neonatal, and nutritional diseases; non-communicable diseases; and injuries. Level 2 consisted of 22 major causes of diseases such as neonatal disorders, cardiovascular diseases, and traffic injuries. Level 3 subdivided Level 2 causes into types such as neonatal preterm birth complications, cerebrovascular disease, and traffic injuries. Level 4 further subdivided those types in some cases—eg, ischaemic stroke and haemorrhagic stroke; and pedestrian road injuries, cyclist road injuries, motorcyclist road injuries, motor vehicle road injuries, and other road injuries.

The leading causes of death were analysed using the Level 3 aggregation of causes of death from the GBD 2017 study (appendix 2 pp 4–13).

### Risk factors

Information on risk factors and their attributable DALYs have been described in detail previously.^21^ Briefly, GBD uses the comparative risk assessment framework developed for previous iterations to estimate levels and trends in exposure, attributable deaths, and attributable DALYs, by age group, sex, year, and location for 84 behavioural, environmental and occupational, and metabolic risks or clusters of risks from 1990 to 2017. The GBD 2017 study included 476 risk–outcome pairs that met the GBD study criteria for convincing or probable evidence of causation. Relative risk and exposure estimates were extracted from 46 749 randomised controlled trials, cohorts, pooled cohorts, household surveys, census data, satellite data, and other sources, according to the GBD 2017 source counting methods. Using the counterfactual scenario of theoretical minimum risk exposure level, the portion of deaths and DALYs that could be attributed to a given risk were estimated.

### SDI and decomposition of change

Since 2015, GBD has estimated the expected burden for each of the three principal measures—deaths, YLLs, and YLDs, with DALYs being the sum of the last two—as a function of each country's SDI.^22^ SDI was first developed for GBD 2015 to provide an interpretable synthesis of overall development, as measured by lag-dependent income per capita, average educational attainment in the population over 15 years of age, and total fertility rates. In GBD 2017, SDI was computed by rescaling each component to a scale of zero to one, with zero being the fewest years of schooling, lowest income per capita, and highest fertility, and one being the most years of schooling, highest income per capita, and lowest fertility, and then taking the geometric mean of these values for each location-year.^17^ Starting with GBD 2016, some modifications have been made to better use each of the scales. The minimum and maximum have been set by examining the relationships each of the inputs had with life expectancy at birth and under-5 mortality and identifying points of limiting returns at both high and low values, if they occurred before theoretical limits.^14^ Furthermore, for GBD 2017, total fertility rate was replaced with fertility rate under 25 years of age, which provides a better measure of women's status in society as it focuses on ages where childbearing disrupts the pursuit of education and entrance into the workforce, and income was replaced with the lag-distributed income per capita.^23^ The average relationship between SDI and disease burden was evaluated for each age-sex-cause group using a smoothing regression spline on SDI for each cause in the GBD cause hierarchy. The estimates were scaled from more detailed causes up to the most aggregated to ensure the total predicted burden at the highest level equalled the sum of the lower levels.^22^

To analyse the drivers of change in disease burden, GBD decomposes trends in diseases and attributable burden into contributions from population growth, changes in population age structures, changes in exposure to environmental and occupational risks, changes in exposure to behavioural risks, changes in exposure to metabolic risks, and changes due to all other factors, approximated as the risk-deleted death and DALY rates. These methods are detailed elsewhere.^21^

### Uncertainty analysis

Uncertainty levels were propagated at multiple stages throughout the GBD modelling process. Uncertainty for mortality and YLLs reflected uncertainty in the levels of all-cause mortality and in the estimation of each mortality cause in each age group, sex, and year. Uncertainty in the disability weight for each sequela was propagated into the estimates of YLDs for each disease and injury. A sample of 1000 draws was taken from the posterior distribution of each estimation step; aggregation of uncertainty across age, sex, and location was done on each draw, assuming independence of uncertainty. The lower and upper uncertainty intervals (UIs) represent the ordinal 25th and 975th draws of each quantity and attempt to describe modelling as well as sampling error.^24^ 95% UIs take into account the uncertainty in the epidemiological parameters used to estimate YLLs, YLDs, and DALYs.

### Role of the funding source

The funder of the study had no role in study design, data collection, data analysis, data interpretation, or writing of the report. The corresponding author had full access to all the data in the study and had responsibility for the decision to submit for publication.

## Results

**Figure 2:**
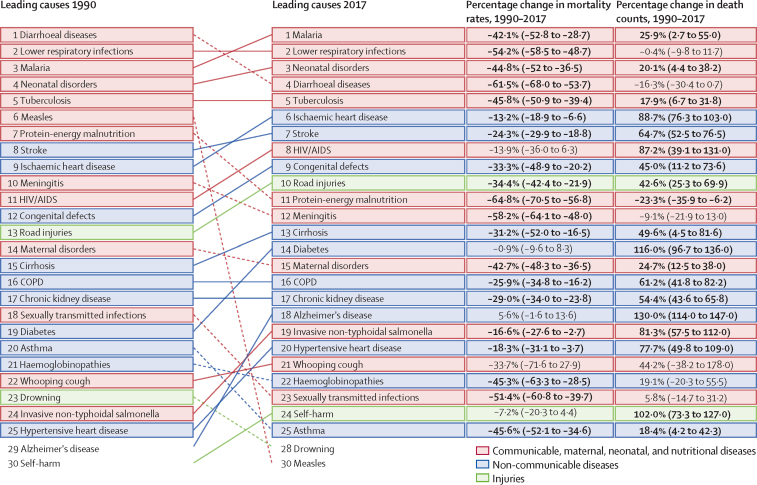
Leading 25 Level 3 causes of death and their evolution, all ages, in francophone Africa, 1990–2017 Data in parentheses are 95% uncertainty intervals. Solid lines indicate increases and dashed lines indicate decreases in rank between periods. Significant changes are shown in bold. COPD=chronic obstructive pulmonary disease.

**Figure 3:**
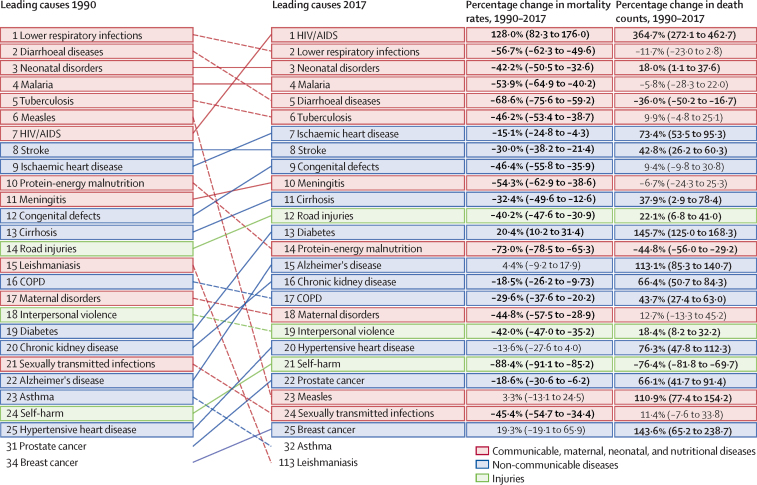
Leading 25 Level 3 causes of death and their evolution, all ages, in non-francophone African countries of the three economic communities, 1990–2017 Data in parentheses are 95% uncertainty intervals. Solid lines indicate increases and dashed lines indicate decreases in rank between periods. Significant changes are shown in bold. COPD=chronic obstructive pulmonary disease.

In 2017, 779 deaths (95% UI 750–809) per 100 000 population occurred in francophone Africa, a decrease of 45·3% since 1990 (appendix 2 pp 26–27). Malaria, lower respiratory infections, neonatal disorders, diarrhoeal diseases, and tuberculosis were the top five Level 3 causes of death in francophone countries (figure 2). All five categories of causes of death decreased during the period 1990–2017 (figure 2). The leading causes differed slightly for the non-francophone African countries from the three economic communities, where HIV/AIDS was the leading cause of death, followed by lower respiratory infections, neonatal disorders, malaria, and diarrhoeal diseases (figure 3). Mortality rates from the top five causes in francophone Africa also decreased in non-francophone Africa. Mortality rates from the top five causes of death generally decreased to a lesser extent in francophone countries than in non-francophone countries, between 1990 and 2017, when each economic community is considered separately (appendix 2 pp 14–19). In francophone African countries, HIV/AIDS was ranked the eighth cause of death in 2017 whereas ischaemic heart disease was sixth (figure 2). Although Niger had the highest mortality rate in 1990 with 2080 deaths (95% UI 1960–2200) per 100 000 population, the Central African Republic led this rate in 2017 with 1380 deaths (1210–1570) per 100 000 population (appendix 2 pp 26–27). The lowest mortality rate in 2017 was observed in Equatorial Guinea at 540 deaths (413–702) per 100 000 population, a decrease of 69·5% since 1990.

**Figure 4:**
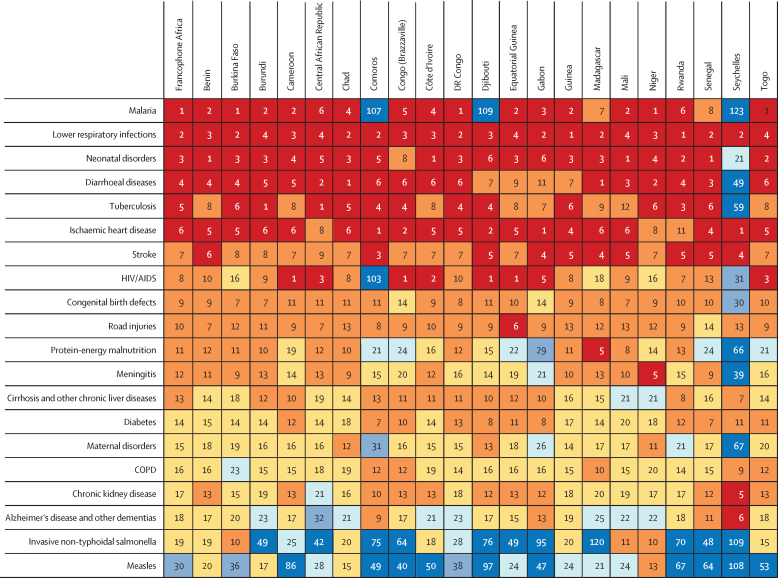
Ranking of main Level 3 causes of death, all ages, by country in francophone Africa, 2017 Causes are organised by overall ranking in francophone Africa. COPD=chronic obstructive pulmonary disease.

When considering the main Level 3 causes of death according to their country-specific mortality burden, malaria, lower respiratory infections, neonatal disorders, diarrhoeal diseases, and ischaemic heart disease are found among the top five causes of death in most countries (figure 4), with some exceptions. For instance, malaria ranks far down the list in Comoros, Djibouti, and the Seychelles. Tuberculosis is ranked between first and tenth place among causes of death in most countries of francophone Africa, although it occupies the 59th rank in the Seychelles. For HIV/AIDS, there is substantial variation in disease rank, being among the top five causes of death for Cameroon, the Central African Republic, Congo, Côte d'Ivoire, Djibouti, Equatorial Guinea, and Gabon, while coming 31st in the Seychelles and 103rd in Comoros.

Neonatal disorders were the leading cause of YLLs in francophone Africa in 2017, followed by malaria, lower respiratory infections, diarrhoeal diseases, and congenital birth defects (appendix 2 p 20). HIV/AIDS, malaria, and diarrhoeal diseases were the second, fourth, and fifth leading cause of YLLs in the remaining African countries, respectively, with neonatal disorders and lower respiratory infections being at the same ranks as in francophone Africa (appendix 2 p 21).

In 2017, francophone Africa experienced 53 570 DALYs (95% UI 50 164–57 361) per 100 000 population, distributed between 43 708 YLLs (41 673–45 742) and 9862 YLDs (7331–12 749), and a life expectancy of 64·0 years (62·1–65·7; appendix 2 pp 26–27). The Seychelles was the healthiest country with 29 200 DALYs (26 500–32 100) per 100 000 population and a life expectancy of 73·6 years (73·1–74·1), the highest among the 21 countries. The Seychelles was followed directly by a low-income country (Comoros) and a low-middle-income country (Djibouti; appendix 2 p 22). Meanwhile, the Central African Republic recorded the highest burden of morbidity with 89 700 DALYs (77 900–105 000) per 100 000 population and the lowest life expectancy among the 21 countries at 51·9 years (49·8–54·1; appendix 2 pp 26–27). Males have borne a higher burden of morbidity than females in most countries of the region (appendix 2 p 22). The Central African Republic had the greatest difference between the sexes with, on average, 19 300 more DALYs per 100 000 population among males.

Based on each country's SDI, the observed DALY burden in 2017 was lower than that expected in 12 of the 21 francophone African countries, with a higher burden than expected in the Central African Republic, Congo, and Gabon. SDI-based expected DALYs ranged from 29 077 DALYs per 100 000 population in the Seychelles to 123 594 DALYs in Niger; despite having the highest observed morbidity burden, the Central African Republic had the eighth highest expected burden, with an expected 68 563 DALYs per 100 000 population (appendix 2 pp 26–27).

In 2017, the major risk factors contributing to the total burden of disease in terms of DALYs were behavioural (appendix 2 p 23). The main risks contributing to neonatal disorders were child and maternal malnutrition. Unsafe water, sanitation, and handwashing followed by child and maternal malnutrition were the main risk factors contributing to diarrhoeal diseases. Air pollution, maternal and child malnutrition, tobacco, and alcohol use were the main risk factors of lower respiratory infections, whereas alcohol use, high fasting plasma glucose, and tobacco were the main risk factors of tuberculosis.

The change in burden (ie, DALYs) between 1990 and 2017 for the 30 leading causes of death in francophone Africa varied considerably among the different causes (appendix 2 p 24). When decomposing these changes in burden, the percentage contributions of population growth, population ageing, and age-specific and cause-specific mortality also varied by cause (appendix 2 p 24). For example, age-specific and cause-specific mortality have contributed to a small decrease in DALYs for Alzheimer's disease and other dementias, but population growth and population ageing have driven a large increase. By contrast, in the case of measles, population ageing has had almost no effect and the large decrease in DALYs has been driven by age-specific and cause-specific mortality, with population growth driving a smaller increase. Changes in age-specific and cause-specific mortality and population ageing were responsible for most of the reductions, while population growth was responsible for most of the increases in disease burden (appendix 2 p 24). For most of the 30 leading causes of death, with the exception of diarrhoeal diseases, meningitis, measles, and protein-energy malnutrition, the total percentage variation was positive, indicating an increase in burden (appendix 2 p 24).

**Figure 5:**
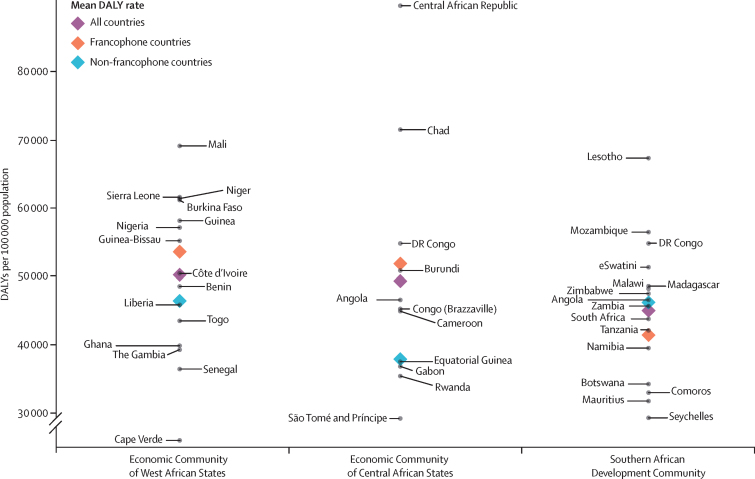
DALYs in francophone and non-francophone African countries, within the three economic communities Francophone countries are labelled to the right and non-francophone countries are labelled to the left. DALY=disability-adjusted life-year.

**Figure 6:**
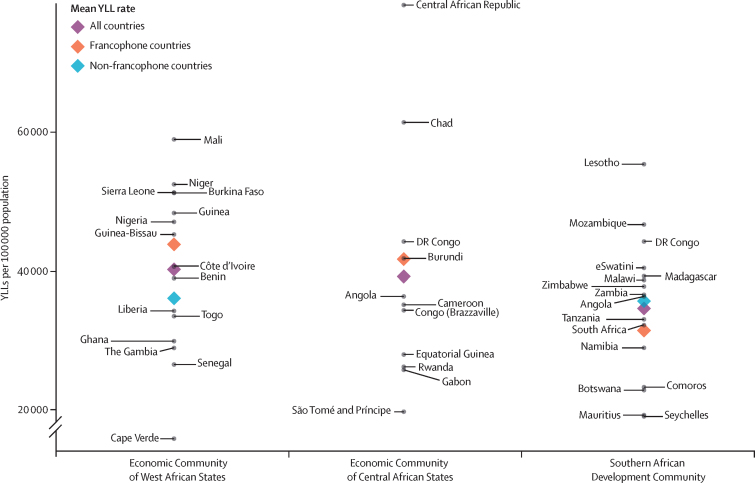
YLLs in francophone and non-francophone African countries, within the three economic communities Francophone countries are labelled to the right and non-francophone countries are labelled to the left. YLL=year of life lost.

Overall, when assessed with DALYs, the burden of disease is higher in francophone African countries than in their non-francophone counterparts within each economic community, except in the Southern African Development Community where Comoros and the Seychelles drive the burden down (figure 5). The same pattern is observed for YLLs (figure 6), but not for YLDs (appendix 2 p 25), where the mean rate is lower for francophone countries in the Economic Community of West African States and in the Southern African Development Community.

## Discussion

Francophone African countries have reduced their mortality by more than 45% since 1990. To our knowledge, this analysis is the first to measure the burden of disease specifically in francophone Africa and to show a decrease in morbidity and mortality, but also a persistent burden of mostly communicable diseases and the beginnings of a rise of non-communicable ones. Our study also shows the higher burden of disease in francophone African countries compared with their non-francophone counterparts within three different economic communities.

While communicable diseases such as diarrhoeal diseases, tuberculosis, and lower respiratory tract infections still weigh on the health of the population, chronic diseases such as ischaemic heart disease and stroke have a growing impact in francophone African countries as their rank as top causes of death increases. Moreover, the burden of disease is dominated by mortality, highlighting the inability of the health systems of francophone Africa to effectively manage morbidity among the population. The Healthcare Access and Quality Index (HAQI), which is measured on a scale of 0 to 100, supports this hypothesis.^25^ To illustrate this, consider the Central African Republic, which has a HAQI of 18·6—the lowest worldwide—and Equatorial Guinea, which has a HAQI of 49·3. While Equatorial Guinea has a lower incidence of neonatal disorders than the Central African Republic (550·8 new cases *vs* 626·6 new cases per 100 000, respectively), the two countries have a very similar prevalence (2275·5 prevalent cases *vs* 2303·8 prevalent cases per 100 000); however, Equatorial Guinea has a much higher rate of YLDs (302·1 *vs* 252·8 in the Central African Republic).^12^ Hence, those affected by neonatal disorders tend to live longer in Equatorial Guinea and die sooner in the Central African Republic. In francophone Africa, only the Seychelles exceeds the halfway point of 50, with a value of 65·6. Thus, an exceptional challenge is awaiting francophone Africa where health systems are far from controlling the burden of communicable and neonatal diseases, and are not yet equipped to cope with the rise of chronic non-communicable diseases. Additionally, the burden of disease in francophone Africa differs from that in its non-francophone counterparts, where HIV/AIDS is the leading cause of death and where most of the leading causes are decreasing at a faster rate than in francophone Africa.

The observed results related to changes in causes of death can also be framed within the theory of health transition.^26^, ^27^ However, there are other factors to keep in mind. First, income determines the quality of health care that a population can afford, as well as its diet, which in turn has a direct influence on mortality through malnutrition. The existing literature reports a decline in mortality as country income level increases.^28^, ^29^ Nevertheless, despite their low income levels, Comoros and Djibouti have some of the best performances, directly below that of the Seychelles, and even better than those of some upper-middle-income countries such as Gabon and Equatorial Guinea. Moreover, apart from these unexpected performances, Cameroon, although a low-middle-income country, has performed well above low-income countries such as Benin, Togo, Madagascar, the Democratic Republic of the Congo, Burundi, and Guinea, as well as the low-income countries mentioned above. Hence, poverty should not equal a death verdict. The Salud Mesoamérica initiative, a maternal and child health initiative in Central America, targets the poorest populations in Central America who are living in conditions that are the same or worse than those in Africa.^30^, ^31^ This results-based funding initiative has proven to be effective in conditions similar to Africa, indicating hope for any population wishing to improve its health situation.^32^ Second, with some exceptions, excess mortality in males is a demographic phenomenon frequently observed in demographic analyses. Our results corroborate this factor as the overall burden, mostly driven by YLLs, is higher for males in most countries.

Despite the existence of effective malaria prevention and treatment strategies; vaccines against rotavirus, pneumonia, and influenza;^33^ and effective antibiotics for the treatment of tuberculosis, we found that malaria, lower respiratory infections, diarrhoeal diseases, and tuberculosis constitute four of the five leading causes of death in francophone Africa. Insecticide-treated mosquito nets can prevent malaria but access to these nets among at-risk populations exceeds 80% only in Senegal, Mali, and Rwanda.^34^ Coverage is less than 50% in countries such as Gabon or Congo. The pneumococcal conjugate vaccine and the rotavirus vaccine still have not been introduced in many countries in francophone Africa.^35^ Otherwise, the coverage of these two vaccines is still not at an optimal level. It has been shown that effective coverage of vaccines is often lower than the reported crude coverage due to problems with health information systems and cold chains in Africa and other parts of the world.^36^ Additionally, the Central African Republic and Congo are among the top ten high-burden countries for tuberculosis based on severity of disease burden (incidence per capita).^37^

Conventionally, and like everywhere in Africa, planning for health in francophone Africa has been focused on communicable diseases. However, with the rise of non-communicable chronic diseases, especially those caused by dietary and behavioural risk factors, this region needs to adopt preventive interventions. Specifically, the region should strengthen its surveillance to follow epidemiological trends and progress achieved, promote health and support healthy behaviours, improve health care and other evidence-based preventive services and their effective utilisation, and link health-care services to community programmes to improve and maintain care for chronic diseases.^38^

As our results on observed versus expected burden show, most countries, including the poorest, fared better than expected. This is mostly true in terms of mortality but not morbidity. In most francophone African countries, the observed burden approached or exceeded the expected burden in terms of YLDs (appendix 2 pp 26–27), pointing out the persistent weakness in disease management. Indeed, with the exception of Comoros, Djibouti, Gabon, Rwanda, and the Seychelles, francophone African countries are all among the lowest quintile, with scores of 22–45, of the universal health coverage index, which ranges from 22 to 86.^39^ In terms of HAQI, 18 (86%) of 21 francophone African countries are in the lowest quintile, with the exceptions of Gabon, Equatorial Guinea and the Seychelles, whereas only 11 (55%) of their 20 non-francophone counterparts in the three economic communities studied are in this same quintile. Since 1990, the share of development assistance for health dedicated to sector-wide approaches and health-system strengthening has constantly decreased in francophone African countries, to the point where it constituted less than 15% in any given country in 2015, except for the Seychelles, Equatorial Guinea, and Benin.^40^ Overall, less than 6% of development assistance for health in 2015 was dedicated to sector-wide approaches and health-system strengthening in the 21 countries combined. Interestingly, HAQI increased with the increase in the ratio of development assistance for health dedicated to sector-wide approaches and health-system strengthening received between 1990 and 2016 to the country population size in 2016.^41^ For instance, this ratio exceeded US$146 per person in the Seychelles but dropped below $7 per person in the Democratic Republic of the Congo.

Our study has several limitations in addition to its methodological advances. A section detailing all the limitations of the GBD study is published elsewhere.^14^ Briefly, and as with any epidemiological analysis, the accuracy of the estimates depends largely on the availability of data sources and the amount of data for a given period. In the absence of specific data, the estimates depend on the covariates and the statistical model used. Furthermore, GBD's modelling process is by cause, not locations. This means that the full location hierarchy (ie, all modelled locations for GBD) is modelled uniquely for each cause. The GBD modelling strategy allows the more data-sparse locations (by cause, age, and sex) to draw strength from more data-rich locations within the location's GBD region and super-region to inform the estimates while—in the same model—the data-rich locations are less reliant on other data inputs. A cause-of-death star rating for each African francophone country is shown in the appendix (p 28). The star rating system is based on vital registration data and produces a rating for each national location from 1 to 5 stars.^42^ Most of the francophone African countries have a rating of 0 while a few have 1. This suggests that all locations must have borrowed a considerable amount of strength from other locations within their region and super-region in the GBD location hierarchy. For these reasons, GBD estimates are produced with UIs to help readers to better weigh and contextualise every estimate produced. In addition, the quality of the available data sometimes varies due to sampling and non-sampling bias for the same country, which increases the uncertainty in comparing the same measure across several countries.

Francophone Africa carries a disproportionate burden of disease, probably due to the weakness of its health-care systems and services. To cope with this burden, francophone Africa should define its priorities and invest more resources in strengthening its health systems and in the quality and quantity of health-care services, especially in rural and remote areas. The three economic communities can set up common goals to decrease intracommunity health disparities through different assistance models within a reflective knowledge environment and knowledge-exchange forums. The region could also be prioritised in terms of technical and financial assistance focused on strengthening health systems and health services, as much as on demographic investments. Several methods of financing can be used—in particular, results-based financing in a solid accounting framework, and a regional approach different from the traditional one followed, which consists of targeting each country alone.^32^, ^43^

## Supplementary Materials

French translation of the article
