# Supplementary material for: Burden of disease in francophone Africa, 1990–2017: a systematic analysis for the Global Burden of Disease Study 2017
Source: Lancet Glob Health. 2020 Feb 19;8(3):e341–51. doi: 10.1016/S2214-109X(20)30024-3 (PMC7034333; doi:10.1016/S2214-109X(20)30024-3)
Supplement: French translation of the article [file mmc1.pdf]

THE LANCET
Global Health

Supplementary appendix 1

This translation in French was submitted by the authors and we reproduce it as supplied. It has not been peer reviewed. *The Lancet Global Health*'s editorial processes have only been applied to the original in English, which should serve as reference for this manuscript.

Supplement to: El Bcheraoui C, Mimche H, Miangotar Y, et al. Burden of disease in francophone Africa, 1990–2017: a systematic analysis for the Global Burden of Disease Study 2017. *Lancet Glob Health* 2020; **8:** e341–51.

Cette traduction en français a été proposée par les auteurs et nous l'avons reproduite telle quelle. Elle n'a pas été examinée par des pairs. Les processus éditoriaux de *Lancet Global Health* n'ont été appliqués qu'à l'original en anglais, ce qui devrait servir de référence à ce manuscrit.

Supplément à: El Bcheraoui C, Mimche H, Miangotar Y, et al. Fardeau de la morbidité en Afrique francophone, 1990–2017 : une analyse systématique de l'étude de la charge mondiale de la morbidité de 2017. *Lancet Glob Health* 2020; **8:** e341–51.

Fardeau de la morbidité en Afrique francophone, 1990–2017 : une analyse systématique de l'étude de la charge mondiale de la morbidité de 2017

Charbel El Bcheraoui, Honoré Mimche, Yodé Miangotar, Varsha Sarah Krish, Faye Ziegeweid, Kris J Krohn, Martin Herbas Ekat, Jobert Richie Nansseu, Zacharie Tsala Dimbuene, Helen Elizabeth Olsen, Roger C K Tine, Christopher M Odell, Christopher E Troeger, Nicholas J Kassebaum, Tamer Farag, Simon I Hay, Ali H Mokdad

Institute for Health Metrics and Evaluation (C El Bcheraoui PhD, V S Krish BA, F Ziegeweid MPH, K J Krohn MPH, C M Odell MPP, C E Troeger MPH, N J Kassebaum MD, T Farag PhD, Prof S I Hay FMedSci, Prof A H Mokdad PhD)**, Department of Health Metrics Sciences, School of Medicine** (C El Bcheraoui, Prof S I Hay, Prof A H Mokdad)**, and Department of Anesthesiology & Pain Medicine** (N J Kassebaum)**, University of Washington, Seattle, WA, USA; Institut de Formation et de Recherche Démographiques, Université de Yaoundé II, Yaoundé, Cameroon** (Prof H Mimche PhD)**; Faculté des Sciences Humaines et Sociales, Université de N'Djaména, N'Djaména, Tchad** (Y Miangotar PhD)**; National AIDS Control Program, Ministry of Health and Population, Brazzaville, Congo** (M H Ekat MD)**; Department for the Control of Disease, Epidemics and Pandemics, Ministry of Public Health, Yaoundé, Cameroon** (J R Nansseu MD)**; Department of Public Heath, Faculty of Medicine and Biomedical Sciences, Université de Yaoundé I, Yaoundé, Cameroun** (J R Nansseu)**; Department of Population Sciences and Development, Faculty of Economics and Management, University of Kinshasa, Kinshasa, République Démocratique du Congo** (Z Tsala Dimbuene PhD)**; Microdata Access Division, Statistics Canada, Ottawa, ON, Canada** (Z Tsala Dimbuene)**; Global Health, Bill & Melinda Gates Foundation, Seattle, USA** (H E Olsen MA)**; and Service de Parasitologie, Faculté de Médecine, de Pharmacie et d'Odontologie, Université Cheikh Anta Diop, Dakar, Sénégal** (Prof R C K Tine PhD)

Correspondance : Dr Charbel El Bcheraoui, Robert Koch Institute, Berlin 13353, Germany **el-bcheraouic@rki.de**

Note : Prière de consulter l'annexe à la fin de l'article

Résumé

Contexte La littérature sur la santé évaluée par des pairs est presque exclusivement publiée en anglais, ce qui limite l'utilisation de la recherche pour la prise de décision dans les pays Africains francophones. Nous avons utilisé les résultats de l'étude de la charge mondiale de la morbidité (CMM) pour évaluer le fardeau de la maladie en Afrique francophone et en informer les professionnels de la santé et leurs partenaires dans la région.

Méthodes Nous avons évalué la charge de morbidité dans les 21 pays francophones d'Afrique et comparé les résultats avec ceux de leurs homologues non-francophones dans trois communautés économiques : la Communauté Economique des États de l'Afrique de l'Ouest, la Communauté Economique des États de l'Afrique Centrale et la Communauté de Développement de l'Afrique Australe. La CMM 2017 a utilisé une variété de modèles statistiques pour déterminer le nombre de décès attribué à chaque cause, par le biais de l'algorithme du modèle d'ensemble des causes de décès (CODEm).[22] Pour s'assurer que le nombre de décès par cause ne dépasse pas le nombre total de décès estimé, une technique de correction appelée CoDCorrect a été utilisée. Après avoir produit des estimations du nombre de décès pour chacune des 282 causes fatales incluses dans la liste des causes de l'étude CMM 2017, les années de vie perdues (AVPs) dues à un décès prématuré ont été calculées. Les années vécues avec incapacité (AVIs) ont été estimées comme le produit de la prévalence et d'un poids d'incapacité pour toutes les séquelles mutuellement exclusives. Les années de vie ajustées de l'incapacité (AVAIs) ont été calculées comme étant la somme des AVPs et des AVIs. Tous les calculs sont présentés avec des intervalles d'incertitude (II) de 95%. Un échantillon de 1000 tirages a été prélevé de la distribution postérieure de chaque étape d'estimation ; l'agrégation de l'incertitude selon l'âge, le sexe et le lieu a été faite pour chaque tirage, en supposant l'indépendance de l'incertitude. Les II inférieur et supérieur représentent les 25ème et 975ème tirages ordinaux de chaque quantité et tentent de décrire la modélisation ainsi que l'erreur d'échantillonnage.

Résultats En 2017, 779 (750–809) décès pour 100 000 habitants sont survenus en Afrique francophone, soit une diminution de 45·3% depuis 1990. Le paludisme, les infections des voies respiratoires inférieures (IRI), les troubles néonatals, les maladies diarrhéiques et la tuberculose étaient les cinq principales causes de décès de niveau 3, respectivement. Ces cinq maladies figuraient parmi les six principales causes de décès dans la plupart des pays francophones. En 2017, l'Afrique francophone comptait 53 569 (50 164–57 361) AVAIs pour 100 000 habitants, réparties entre 43 708 (41 673–45 742) AVPs et 9862 (7331–12 749) AVIs pour 100 000 habitants. En 2017, les AVPs représentent la quasi-totalité des AVAIs dans

les 21 pays d'Afrique francophone. La mortalité par âge et par cause et le vieillissement de la population furent responsables de la plupart des réductions, tandis que la croissance démographique fut responsable de la plupart des augmentations de la charge de morbidité.

Conclusions L'Afrique francophone porte encore un lourd fardeau de maladies transmissibles et néonatales, probablement en raison de la faiblesse des systèmes de santé et services de soins, comme en témoigne l'attribution presque complète des AVAIs aux AVPs. Pour faire face à ce fardeau de la maladie, l'Afrique francophone devrait définir ses priorités et investir davantage de ressources dans le renforcement des systèmes de santé, dans la qualité et la quantité des services de soins de santé, en particulier dans les zones rurales et éloignées. La région pourrait également être prioritaire en termes d'assistance technique et financière axée sur le renforcement des systèmes et des services de santé, tout autant que sur les investissements démographiques, y compris l'éducation et la planification familiale.

Financement la Fondation Bill & Melinda Gates.

La recherche dans son contexte

Preuves avant cette étude

Alors que les précédentes itérations de l'étude de la Charge Mondiale de la Morbidité, les Blessures et les Facteurs de Risque (CMM), y compris la CMM 2016, ont fourni des estimations concernant les maladies et les incapacités dans 195 pays et territoires, elles n'ont pas fourni d'estimations sur la charge en Afrique francophone en tant que région. Les données sur cette région ont souvent été limitées aux rapports épidémiologiques des ministères de santé et des enquêtes périodiques. Il est important de noter que la littérature sur la santé dans la région, y compris la CMM est presque exclusivement publiée en anglais, ce qui limite l'utilisation de la recherche pour la prise de décision dans les pays Africains où le français est une langue officielle.

Valeur ajoutée de cette étude

Cette étude dresse un tableau complet de la santé et de ses tendances depuis 1990 dans tous les pays Africains francophones, dans une langue (le français) plus accessible à ces pays. L'étude souligne le retard de l'Afrique francophone le long de l'axe épidémiologique par rapport à d'autres régions d'Afrique, ainsi que les disparités entre francophones et non-francophones au sein de trois grandes communautés économiques Africaines. Les conclusions de cette étude permettront aux scientifiques et aux décideurs de l'Afrique francophone d'utiliser les meilleurs des preuves pour les programmes de santé locaux.

Implications de toutes les preuves disponibles

Cette analyse permet aux pays Africains francophones d'accéder aux estimations les plus récentes et les plus complètes de la charge de morbidité en termes d'années de vie perdues, d'années vécues avec une incapacité, d'années de vie ajustées de l'incapacité, d'espérance de vie, et de l'évolution de la charge de morbidité depuis 1990. Les résultats mettent en évidence les besoins les plus urgents en termes d'assistance financière pour la région, avec une attention particulière au renforcement des systèmes de santé.

Introduction

L'Afrique Francophone comprend 21 pays répartis entre les parties Ouest, Centre, Est et Nord de l'Afrique, et où la langue française est une langue officielle du pays (figure 1).[1] Alors que la langue Française pourrait être utilisée par les populations des autres pays d'Afrique comme l'Algérie, la Tunisie, ou le Maroc, elle n'y est pas une langue officielle.[2] ainsi, l'Afrique francophone inclut le Bénin, le Burkina Faso, le Burundi, le Cameroun, les Comores, le Congo, la Côte d'Ivoire, le Djibouti, la Guinée, la Guinée Equatoriale, le Gabon, le Madagascar, le Mali, le Niger, la République Centrafricaine, la République Démocratique du Congo, le Rwanda, le Sénégal, les Seychelles, le Tchad, et le Togo. Cette région compte plus de 324 334 000 habitants, représentant 25·2% de la population Africaine.[3]

Au sein de la région, les pays Africains francophones font partie de différents groupes économiques qui promeuvent les collaborations au développement. Les plus importantes d'entre elles sont la Communauté Economique des États de l'Afrique de l'Ouest (15 pays, dont neuf sont francophones), la Communauté Economique des États d'Afrique Centrale (11 pays, dont 9 francophones), et la Communauté de Développement de l'Afrique Australe (16 pays, dont quatre francophones), avec La République Démocratique du Congo étant membre des deux derniers groupes (annexe joint à la fin de l'article p 3). Toutefois, au sein de chacune de ces communautés, les pays francophones montrent de plus faibles indicateurs de développement qui peuvent avoir un effet négatif sur la santé de leur populations. Par exemple, en ce qui concerne l'indice sociodémographique (IDS) —une mesure composite du revenu par habitant, le nombre moyen d'années d'études après l'âge de 15 ans et le taux de fécondité avant l'âge de 25 ans— les pays francophones de ces trois groupes économiques ont une moyenne de 0·33, 0·44 et 0·48 contre 0·45, 0·48, et 0·52 pour les non-francophones respectivement.[4]

La transition épidémiologique est fortement influencée par le développement social et économique, y compris celui des systèmes de la santé.[5] Ainsi, avec leurs inconvénients sociodémographiques et des systèmes de santé plus faibles, les pays Africains francophones pourraient se trouver à un stade différent de leur transition épidémiologique, et faisant face à des profils épidémiologiques, et des défis sanitaires différents de ceux de la région. Alors que la prévalence des maladies non transmissibles augmente en Afrique, les maladies infectieuses, les carences nutritionnelles et les complications de la grossesse et de l'accouchement sont encore fréquentes.[4]

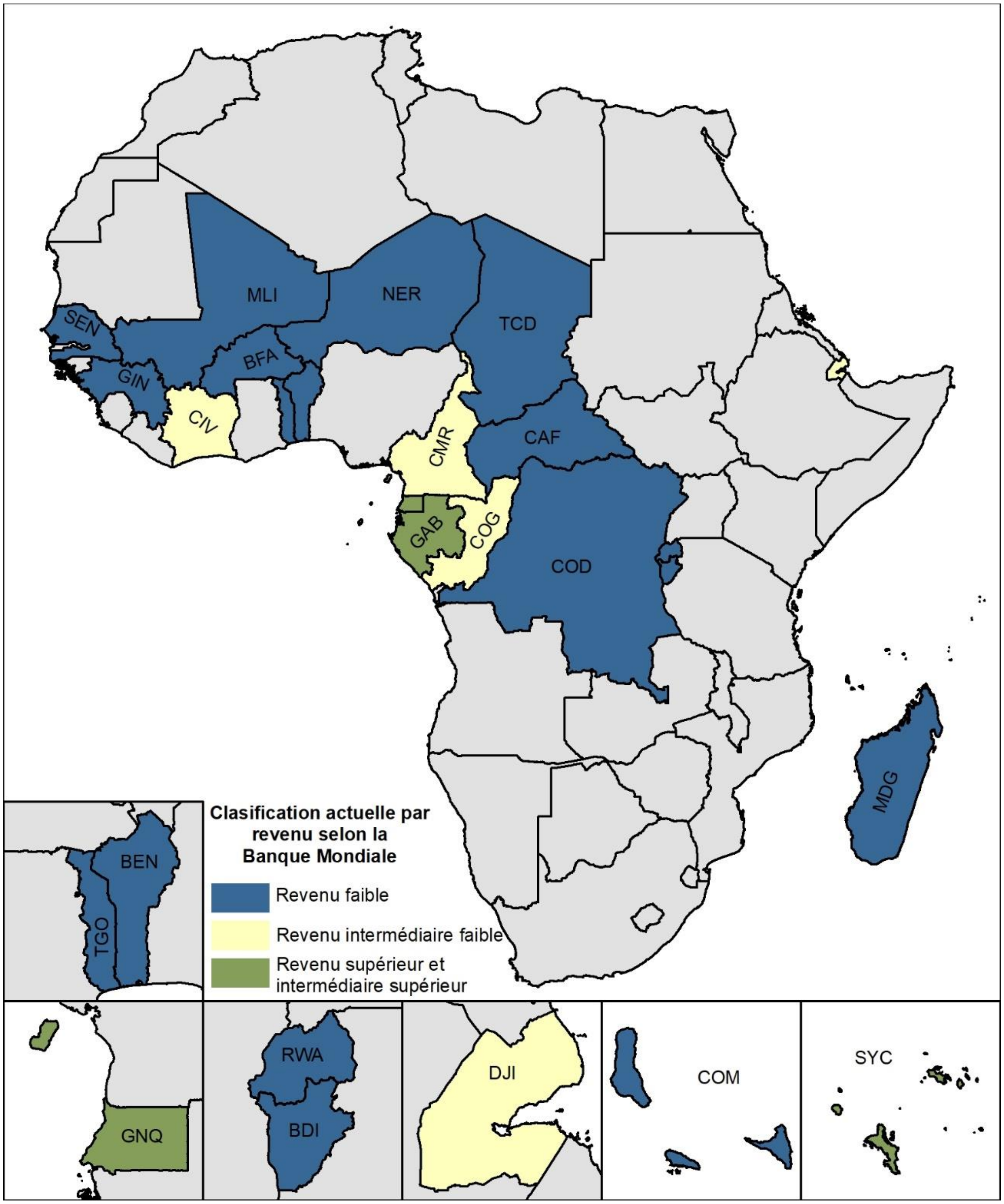

Figure 1: pays de l'Afrique francophone classifiés selon le niveau de revenu

Le fardeau de la maladie est la pierre angulaire de la prise de décisions sur la santé de la population.[6] Les décideurs de la santé l'utilisent pour décrire et tenter de comprendre les problèmes de santé au niveau de la population, en vue de prioriser les questions correctives. Ainsi, la disponibilité de données solides en temps opportun est un élément essentiel de la prise de décisions en matière de santé. Cependant, avec la quasi-domination de la littérature scientifique, et en particulier celle revue par les pairs, par la langue anglaise, la communauté scientifique en Afrique francophone, y compris les décideurs en santé, est isolée de la contribution à, et de l'utilisation de, cette littérature.[7,8] En général, très peu d'écrits sur la santé des populations de ces pays sont disponibles. Par exemple, sur les 246 articles revus par les pairs, publiés entre 1990 et 2015, et originaires d'Afrique de l'Ouest, seuls 37 sont en français.[9] Au niveau des pays Africains francophones, la faiblesse de la production scientifique sur la santé de la population et les systèmes de santé est liée au petit nombre de structures de recherche en santé, au manque de ressources allouées aux universités et à l'absence ou l'insuffisance des systèmes d'information sur la santé.[10]

L'étude de la Charge Mondiale de Morbidité (CMM) est publiée en Anglais depuis 1990, et depuis 2015, l'outil de visualisation de l'étude est publié en Chinois, Espagnol, Japonais, Norvégien, Portugais, Russe, et Suédois permettant l'accès aux résultats de l'étude par une audience plus vaste et ainsi augmentant son utilité autour du monde.[11,12] Malheureusement, il n'existe pas de publications sur la CMM en français, en particulier des publications concernant les pays où le français est la langue principale, ce qui oblige les professionnels de santé locaux à se référer à des publications rédigées en Anglais alors que l'Anglais n'y est parfois pas couramment utilisé. Nous avons utilisé les résultats de l'étude CMM pour évaluer la charge de morbidité en Afrique francophone afin d'informer les professionnels de la santé et leurs partenaires dans la région, et mettre en évidence les disparités sanitaires que connaissent ces pays au sein de leurs réseaux de communautés économiques. Cet article est également publié en français pour assurer l'accessibilité à la communauté francophone dans le monde entier.

Méthodes

Avant que le projet de la CMM ne soit lancé en 1991, aucune évaluation globale de la santé humaine n'avait été effectuée. L'étude de la CMM est un exercice global et comparatif d'estimation des risques, avec les premiers résultats préliminaires (pour l'année de référence 1990) publiés dans le World Development Report 1993.[13] Cet effort collaboratif international est actuellement dirigé par l'Institute for Health Metrics and Evaluation (IHME) à Seattle, Washington, Etats Unis.

L'étude de la CMM utilise des méthodes pour corriger la sous-déclaration des décès et les codes non valides *(garbage codes)*. Ces codes non valides sont des erreurs de classification des décès présentes dans les données. Certains de ces codes représentent des cas où la cause indiquée ne peut logiquement pas avoir provoqué le décès. «Rigidité abdominale», «sénilité» et «syndrome de l'ongle jaune» sont des exemples de codes non valides trouvés dans les dossiers. La correction des codes non valides emploie des preuves de la littérature médicale, les opinions d'experts, et les techniques statistiques pour allouer chaque code aux causes de mortalité les plus probables.[13]

Dans cette étude, nous considérons les 21 pays Africains francophones et comment ils se distinguent de leurs homologues non-francophones dans trois communautés Economiques : la Communauté économique des États de l'Afrique de l'Ouest, la Communauté Economique des États de l'Afrique centrale, et la Communauté de Développement de l'Afrique Australe (annexe p 3). Nous évaluons l'évolution des principales causes de décès en Afrique francophone, par rapport à leur évolution en leurs homologues non-francophones, et classent les principaux causes de décès dans chacun des pays Africains francophones. Nous évaluons la charge de morbidité en fonction du nombre d'années de vie perdues (AVPs) en raison d'un décès prématuré, les années vécues avec incapacité (AVIs), et les années de vie ajustées de l'incapacité (AVAIs). Nous présentons l'évolution des principales causes des AVIs, les totaux des AVAIs par sexe et par pays, la charge prévue sur la base de l'ISD, les principaux facteurs de risque contribuant aux AVAIS, les moteurs de l'évolution de la charge de morbidité dans les pays francophones d'Afrique, et une comparaison de la charge de morbidité entre les pays francophones et non francophones au sein de la trois communautés économiques.

Plus d'informations sur les sources de données, les méthodes d'estimation, les outils de calcul et l'analyse statistique utilisés dans le calcul des estimations de la CMM sont disponibles ailleurs.[14] Les sources de données utilisées pour l'analyse CMM dans les pays d'Afrique francophone sont énumérées à l'annexe 3.

Toutes les sources de données utilisées dans la CMM sont évaluées avant d'être incluses dans l'analyse. Une description détaillée de nos sources de données, leurs limites, et leur utilisation est publiée ailleurs.[15]

Toutes les recherches sur la CMM sont effectuées sur une base de données secondaires du domaine publique, sans identification nominale, conformément avec le décret américain numéro 7724 du 16 mai 2012, et la Résolution numéro 510 du 7 avril 2016 ; il n'y avait donc pas lieu de soumettre cette étude à un comité d'étiques de recherche car aucune approbation éthique n'était requise. Cette analyse est conforme aux lignes directrices Guidelines for Accurate and Transparent Health Estimates Reporting.[16]

Paramètres et désagrégation des causes

Après avoir traité la question de qualité de données, l'étude de la CMM 2017 a utilisé une variété de modèles statistiques pour déterminer le nombre de décès attribuables à chaque cause, par le biais de l'algorithme du modèle des causes de mortalité [*cause of death ensemble model (CODEm)]*. Pour s'assurer que le nombre de décès par cause ne dépassait pas le nombre total de décès estimés, une technique de correction appelée CoCorrect a été utilisée.[17] Cette technique garantit le fait que les estimations du nombre de décès attribuables à chaque cause ne dépassent pas 100% des décès pour une couche donnée d'âge, sexe, et année.[18] Après avoir produit des estimations pour le nombre de décès dus à chacune des 282 causes de décès incluses dans la liste des causes de l'étude CMM 2017, les AVPs à cause de décès prématurés ont été calculées. Pour chaque décès dus à une cause particulière, le nombre d'AVP se fondait sur l'espérance de vie la plus élevée dans la tranche d'âge de la personne décédée.[19,20] Les AVIs ont été estimées en multipliant la prévalence par le coefficient de pondération de l'incapacité pour toutes les séquelles mutuellement exclusives, corrigées pour la comorbidité, et agrégées au niveau des causes.[15] Les AVAIs sont calculées en tant que la somme des AVPs et AVIs. L'étude de la CMM a utilisé une liste de causes désignant 282 causes de décès selon une hiérarchie à quatre niveaux (annexe pp 4–15). Le premier niveau a divisé les causes en trois groupes : 1) les maladies transmissibles, maternelles, néonatales et nutritionnelles, 2) les maladies non transmissibles, et 3) les blessures. Le deuxième niveau est constitué de 22 principales causes de maladies comme les troubles néonataux, les maladies cardiovasculaires et les accidents de la route. Le troisième niveau subdivise le niveau 2 en catégories telles que les complications néonatales de naissance prématurée, les maladies cérébro-vasculaires, et les accidents de la route. Le quatrième niveau subdivise ces types dans certains cas, par exemple : accident vasculaire cérébral ischémique et accident vasculaire cérébral hémorragique ; accidents de la route pour les piétons, accidents de la route pour les cyclistes, accidents de la route pour les motocyclistes, accidents de la route causés par un véhicule à moteur, et autres accidents de la route.

Les principales causes de décès ont été analysées à l'aide de l'agrégation des causes de décès du niveau 3 de l'étude de la CMM 2017 (annexe pp 4–15).

Facteurs de risque

Les renseignements sur les facteurs de risque et les AVAIs qui leur sont attribuables se trouvent ailleurs.[21] Bref, l'étude de la CMM utilise le cadre comparatif d'estimation des risques mis au point pour les itérations précédentes pour estimer les niveaux et les tendances en exposition, décès attribuables, et AVAIs attribuables, par groupe d'âge, sexe, année, et lieu pour 84 facteurs de risque ou groupes de facteurs de risque comportementaux, environnementaux, liés au travail, et métaboliques de 1990 jusqu'en 2017. L'étude de la CMM a inclus 476 paires de risque-résultats qui remplissaient les critères d'étude de la CMM de preuve convaincante ou probable de causalité. Les estimations de risque relatif et d'exposition ont été extraits de 46 749 essais contrôlés randomisés, cohortes, cohortes groupées, enquêtes auprès des ménages, recensements, données satellitaires, et autres sources, selon la méthode de comptage de sources de l'étude de la CMM 2017. En utilisant le scénario contrefactuel du niveau théorique de risque minimal d'exposition, les portions de décès et d'AVAIs qui pourraient être attribuées à un risque donné ont été estimées.

ISD et décomposition de l'évolution

Depuis 2015, l'étude de la CMM a estimé la charge prévue pour chacune des trois mesures (mortalité, AVP et AVI, avec l'AVAIs comme la somme des deux derniers paramètres) en fonction de l'Indice Sociodémographique (ISD) de chaque pays.[22] L'ISD a été développé d'abord pour l'étude CMM 2015 afin d'offrir une synthèse interprétable du développement global tel que mesuré par le revenu par habitant décalé, la scolarité moyenne dans la population âgée de plus de 15 ans, et l'indice synthétique de fécondité. Dans l'étude CMM 2017, l'IDS a été calculé en rééchelonnant chaque composant à une échelle de zéro à un, avec zéro étant le moins d'années de scolarité, le plus bas revenu par habitant, et l'indice synthétique de fécondité le plus haut, et un étant le plus d'années de scolarité, le revenu par habitant le plus élevé, et l'indice synthétique de fécondité le plus bas, et puis en prenant la moyenne géométrique de ces valeurs pour chaque année par pays.[17] À partir de la CMM 2016, certaines modifications ont été apportées pour mieux utiliser chacune des échelles. Le minimum et le maximum ont été fixés en examinant les relations que chacune des entrées avait avec l'espérance de vie à la naissance, et avec la mortalité des moins de 5 ans, et en identifiant des points de limitation des rendements, à la fois aux valeurs élevées et faibles, s'ils se sont produits avant les limites théoriques.[14] En outre, pour la CMM

2017, l'indice synthétique de fécondité fut remplacé par le taux de fécondité des moins de 25 ans. Cet indice permet de mieux mesurer le statut des femmes dans la société car il se concentre sur les âges où la procréation perturbe la poursuite des études et l'entrée dans la vie active. Le revenu a été remplacé par le revenu distribué avec retard par habitant.[23] La relation moyenne entre l'ISD et la charge de morbidité a été évaluée pour chaque groupe d'âge-sexe-cause en utilisant une courbe de régression de lissage sur l'ISD pour chaque cause dans la hiérarchie des causes de la CMM. Les estimations ont été échelonnées à partir des causes les plus détaillées jusqu'aux plus agrégées pour garantir que la charge totale prévue au niveau le plus élevé soit égale à la somme des niveaux inférieurs.[22]

Pour analyser les moteurs de l'évolution de la charge de morbidité, la CMM décompose les tendances des maladies et de la charge imputable en contributions de la croissance démographique, des changements dans la structure d'âge de la population, des changements dans l'exposition aux risques environnementaux et professionnels, des changements dans l'exposition aux risques comportementaux, des changements dans l'exposition aux risques métaboliques, et des changements dus à tous les autres facteurs, approximés comme les taux de décès à risques supprimés et d'AVAI. Ces méthodes sont détaillées ailleurs.[21]

Analyse de l'incertitude

Les niveaux d'incertitude ont été propagés à plusieurs étapes à travers le processus de modélisation de la CMM. L'incertitude pour la mortalité et les AVPs reflète l'incertitude au niveau de la mortalité de toutes causes, ainsi que dans l'estimation de chaque cause de mortalité, dans chaque groupe d'âge, de sexe et d'année. L'incertitude du coefficient de pondération pour chaque séquelle a été propagée dans l'estimation des AVIs pour chaque maladie et blessure. Un échantillon de 1000 tirages est extrait de la distribution postérieure de chaque étape de l'estimation ; l'agrégation de l'incertitude à travers l'âge, le sexe et la géographie a été faite sur chaque tirage, supposant l'indépendance de l'incertitude. Les II inférieure et supérieure représentent le 25ème et le 975ème tirage ordinal de chaque quantité et tentent de décrire l'erreur de la modélisation ainsi que celle de l'échantillonnage.[24] Les 95% II prennent en compte l'incertitude dans les paramètres épidémiologiques utilisés pour estimer les AVPs, AVIs et AVAIs.

Rôle de la source de financement

Le financeur de l'étude n'a joué aucun rôle dans la conception de l'étude, la collecte des données, l'analyse des données, l'interprétation des données ou la rédaction du rapport. L'auteur correspondant avait un accès complet à toutes les données de l'étude et était responsable de la décision de soumettre l'étude pour publication.

Résultats

En 2017, 779 (750–809) décès pour 100 000 personnes ont eu lieu en Afrique francophone, une réduction de 45·3% depuis 1990 (annexe pp 28–29). Le paludisme, les infections respiratoires inférieures (IRIs), les troubles néonataux, les maladies diarrhéiques et la tuberculose furent les cinq premières causes de décès du 3ème niveau, dans les pays francophone (figure 2). Les cinq catégories de causes de décès ont toutes diminué au cours de la période 1990–2017 (figure 2). Les causes principales sont légèrement différentes pour les pays non-francophones des trois communautés économiques, où le VIH/sida fut la principale cause de décès, suivi par les IRIs, les troubles néonatals, le paludisme et les maladies diarrhéiques, avec la tuberculose occupant la sixième place (figure 3).

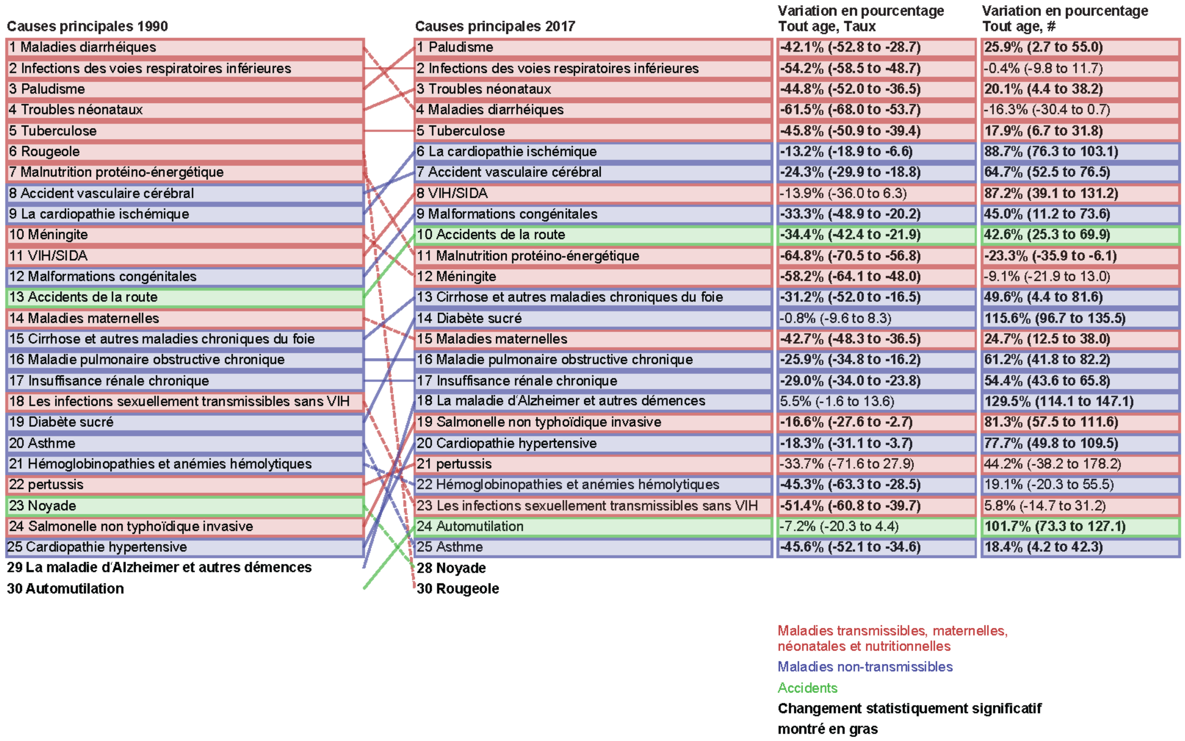

Figure 2: 25 causes principales de décès du niveau 3 et leur évolution, tout âge, Afrique francophone, 1990 - 2017

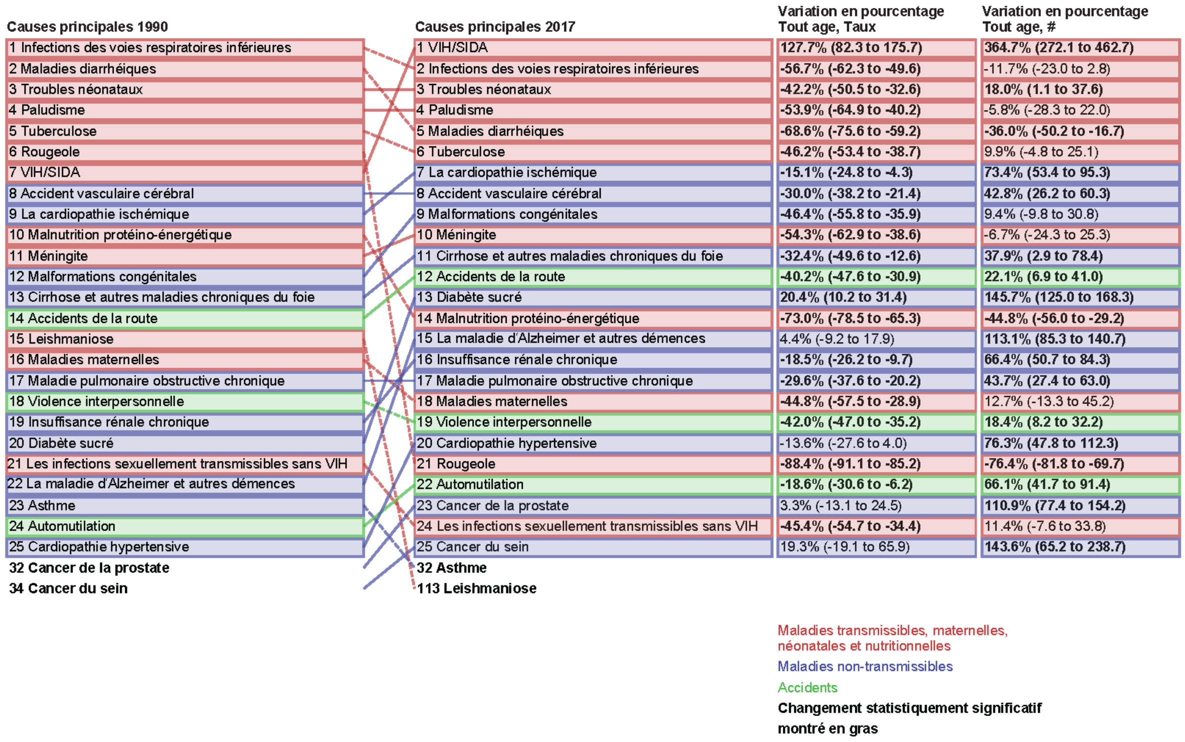

Figure 3: 25 causes principales de décès du niveau 3 et leur évolution, tout âge, Afrique non-francophone, 1990 - 2017

Les taux de mortalité des cinq premières causes en Afrique francophone ont également diminué dans l'Afrique non francophone. Les taux de mortalité dû aux cinq premières causes de décès a diminué dans une moindre mesure dans les pays francophones que dans les pays non-francophones, entre 1990 et 2017, lorsque chaque communauté économique est considérée séparément (annexe p 16–21). Dans les pays Africains francophones, Le VIH/sida a été classé huitième cause de décès en 2017 alors que les maladies cardiaques ischémiques arrivaient en sixième position (figure 2).

Bien que le Niger ait eu le taux de mortalité le plus élevé en 1990 avec 2080 décès (95% II 1960–2200) pour 100 000 habitants, La République Centrafricaine est en tête de ce taux en 2017 avec 1380 décès (1210–1570) pour 100 000 habitants (annexe p 28–29). Le taux de mortalité le plus bas a été observé en 2017 en Guinée Equatoriale à 540 décès (413–702) pour 100 000 habitants, soit une diminution de 69·5% depuis 1990.

Si l'on considère les principales causes de décès de niveau 3 en fonction de la charge de mortalité propre à chaque pays, le paludisme, les IRIs, les troubles néonatals, les maladies diarrhéiques et les maladies cardiaques ischémiques se retrouvent parmi les cinq premières causes de décès dans la plupart des pays, à quelques exceptions près (figure 4). Par exemple, le paludisme se classe loin en bas de la liste aux Comores, à Djibouti, et les Seychelles. La tuberculose est classée entre la première et la dixième place parmi les causes de décès dans la plupart des pays de l'Afrique francophone, bien qu'elle occupe le 59ème rang aux Seychelles. En ce qui concerne le VIH/sida, il y a une variation importante dans le rang de la maladie qui figure parmi les cinq premières causes de décès pour le Cameroun, la République Centrafricaine, le Congo, la Côte d'Ivoire, le Djibouti, la Guinée équatoriale, et le Gabon, tout en étant 31ème aux Seychelles et 103ème aux Comores.

**Afrique Francophone**	Le Benin	Le Burkina Faso	Le Burundi	Le Cameroun	La République Centrafricaine	Le Tchad	Les Comores	Le Congo	La Côte d'Ivoire	La République Démocratique du Congo	Le Djibouti	La Guinée Equatoriale	Le Gabon	La Guinée	Madagascar	Le Mali	Le Niger	Le Rouanda	Le Sénégal	Les Seychelles	Le Togo	
Paludisme	1	2	1	2	107	2	4	6	5	1	4	109	2	2	3	7	2	1	6	8	123	1
Infections des voies respiratoires inférieures	2	3	2	4	2	3	2	4	3	2	3	3	1	4	2	2	4	3	1	2	2	4
Troubles néonataux	3	1	3	3	5	4	3	5	8	3	1	6	3	3	6	3	1	4	2	1	21	2
Maladies diarrhéiques	4	4	4	5	6	5	1	2	6	6	6	7	7	9	11	1	3	2	4	3	49	6
Tuberculose	5	8	6	1	4	8	5	1	4	4	8	4	6	8	7	9	12	6	3	6	59	8
Cardiopathie ischémique	6	5	5	6	1	6	6	8	2	5	5	2	4	5	1	6	6	8	11	4	1	5
Accident vasculaire cérébral	7	6	8	8	3	7	7	9	7	7	7	5	5	7	4	4	5	7	5	5	4	7
VIH/SIDA	8	10	16	9	103	1	8	3	1	10	2	1	8	1	5	18	9	16	7	13	31	3
Malformations congénitales	9	9	7	7	11	11	11	11	14	8	9	11	9	10	14	8	7	9	10	10	30	10
Accidents de transport	10	7	12	11	8	9	13	7	9	9	10	9	13	6	9	12	13	12	9	14	13	9
Malnutrition protéino-énergétique	11	12	11	10	21	19	10	12	24	12	16	15	11	22	29	5	8	14	13	24	66	21
Meningites	12	11	9	13	15	14	9	13	20	16	12	14	10	19	21	13	10	5	15	9	39	16
Cirrhose et autres maladies chroniques du foie	13	14	18	12	13	10	14	19	11	11	11	10	16	12	10	15	21	21	8	16	7	14
Diabète sucré	14	15	14	14	7	12	18	14	10	13	14	8	17	11	8	14	20	18	12	7	11	11
Maladies maternelles	15	18	19	16	31	16	12	16	16	15	15	13	14	18	26	17	17	11	21	17	67	20
Maladie pulmonaire obstructive chronique	16	16	23	15	12	15	19	18	12	14	19	16	15	16	16	10	15	20	14	15	9	12
Insuffisance rénale chronique	17	13	15	19	10	13	16	21	13	18	13	12	18	13	12	20	19	17	17	12	5	13
Maladie d'Alzheimer	18	17	20	23	9	17	21	32	17	23	21	17	19	15	13	25	22	22	18	11	6	18
Salmonelle non typhoïdique invasive	19	19	10	49	75	25	20	42	64	28	18	76	20	49	95	120	11	10	70	48	109	15
Rougeole	30	20	36	17	49	86	15	28	40	38	50	97	24	24	47	21	24	13	67	64	108	53

Figure 4: Classement des causes principales de décès du niveau 3, tout âge, par pays, Afrique Francophone, 2017

Les troubles néonataux furent la principale cause d'AVPs en Afrique francophone, suivis par le paludisme, les IRIs, les maladies diarrhéiques et des malformations congénitales, respectivement (annexe p 22). Le VIH/sida, le paludisme et les maladies diarrhéiques furent la 2ème, 4ème et la 5ème cause principales d'AVPs dans les autres pays Africains respectivement, avec les troubles néonatals et les IRIs aux mêmes rangs qu'en Afrique francophone (annexe p 23).

La charge totale observée et prévue de la morbidité

En 2016, l'Afrique francophone a souffert de 53 570 (50 164–57 361) AVAIs pour 100 000 personnes, distribuées entre 43 708 (41 673–45 742) d'AVPs et 9862 (7331–12 749) d'AVIs pour 100 000 personnes, avec une espérance de vie de 64·0 ans (62·1–65·7 ; annexe pp 28–29). Les Seychelles furent le pays le plus sain avec 29 200 (26 500–32 100) AVAIs pour 100 000 personnes et une espérance de vie de 73·6 (73·1–74·1) ans, la plus élevée parmi les 21 pays. Les Seychelles furent suivies directement par un pays à revenu faible et un autres à revenu intermédiaire faible: Les Comores, et le Djibouti (annexe p 24). Par contre, la République Centrafricaine a enregistré la charge de morbidité la plus élevée avec 89 700 (77 900–105 000) AVAIs pour 100 000 personnes et l'espérance de vie la moins élevée parmi les 21 pays : 51·9 (49·8–54·1) ans (annexe pp 28–29). Les hommes ont dû porter une charge de morbidité plus élevée que les femmes dans la majorité des pays de la région (annexe p 24). La République Centrafricaine présente la différence la plus élevée entre les deux sexes avec, en moyenne, 19 300 AVAIs de plus pour 100 000 personnes chez les hommes.

Selon l'ISD, la charge observée en termes d'AVAIs serait inférieure à celle prévue en 2017 dans 12 des 21 pays de l'Afrique francophone, avec une charge plus élevée que prévu dans la République Centrafricaine, le Congo et le Gabon. Le taux d'AVAIs prévus selon l'ISD varierait entre 29 077 pour 100 000 personnes aux Seychelles et 123 594 au Niger ; bien qu'ayant le plus haut taux observé en termes d'AVAIs, la République Centrafricaine avait la huitième charge prévue la plus élevée, avec 68 563 AVAIs pour 100 000 habitants (annexe pp 28–29).

En 2017, les principaux facteurs de risque contribuant à la totalité de la charge de morbidité, en termes d'AVAIs, étaient comportementaux (annexe p 25). Le risque principal des troubles néonataux était la malnutrition infantile et maternelle. L'eau insalubre, l'assainissement et le lavage des mains, suivis de la malnutrition infantile et maternelle, étaient les facteurs de risque contribuant aux maladies diarrhéiques. La pollution de l'air, la malnutrition maternelle et infantile, le tabac et la consommation d'alcool étaient les principaux facteurs de risque d'infections des voies respiratoires inférieures. La consommation

d'alcool, la glycémie plasmatique élevée à jeun et le tabac étaient les principaux facteurs de risque de la tuberculose.

En décomposant la variation de la charge de la morbidité en termes d'AVAIs entre 1990 et 2017 pour les 30 principales causes de décès en Afrique francophone, les pourcentages contribués par la croissance démographique, le vieillissement de la population et la mortalité par âge et par cause à la variation de la charge varient selon la cause (annexe p 26). L'évolution de l'ensemble des décès a varié considérablement selon les différentes causes. Par exemple, la mortalité par âge et par cause a contribué à une légère diminution des AVAIs pour la maladie d'Alzheimer et d'autres démences, mais la croissance démographique et le vieillissement de la population ont entraîné une forte augmentation. En revanche, dans le cas de la rougeole, le vieillissement de la population n'a eu pratiquement aucun effet et la forte diminution des AVAIs est due à la mortalité par âge et par cause, la croissance de la population entraînant une augmentation plus faible. La mortalité par âge et par cause et le vieillissement de la population sont responsables de la plupart des réductions, tandis que la croissance démographique est responsable de la plupart des augmentations de la charge de morbidité (annexe p 26). Pour la plupart des 30 principales causes de décès, à l'exception de la malnutrition protéino-énergétique, de la méningite, de la rougeole et des maladies diarrhéiques, le total du pourcentage de la variation est positif (annexe p 26).

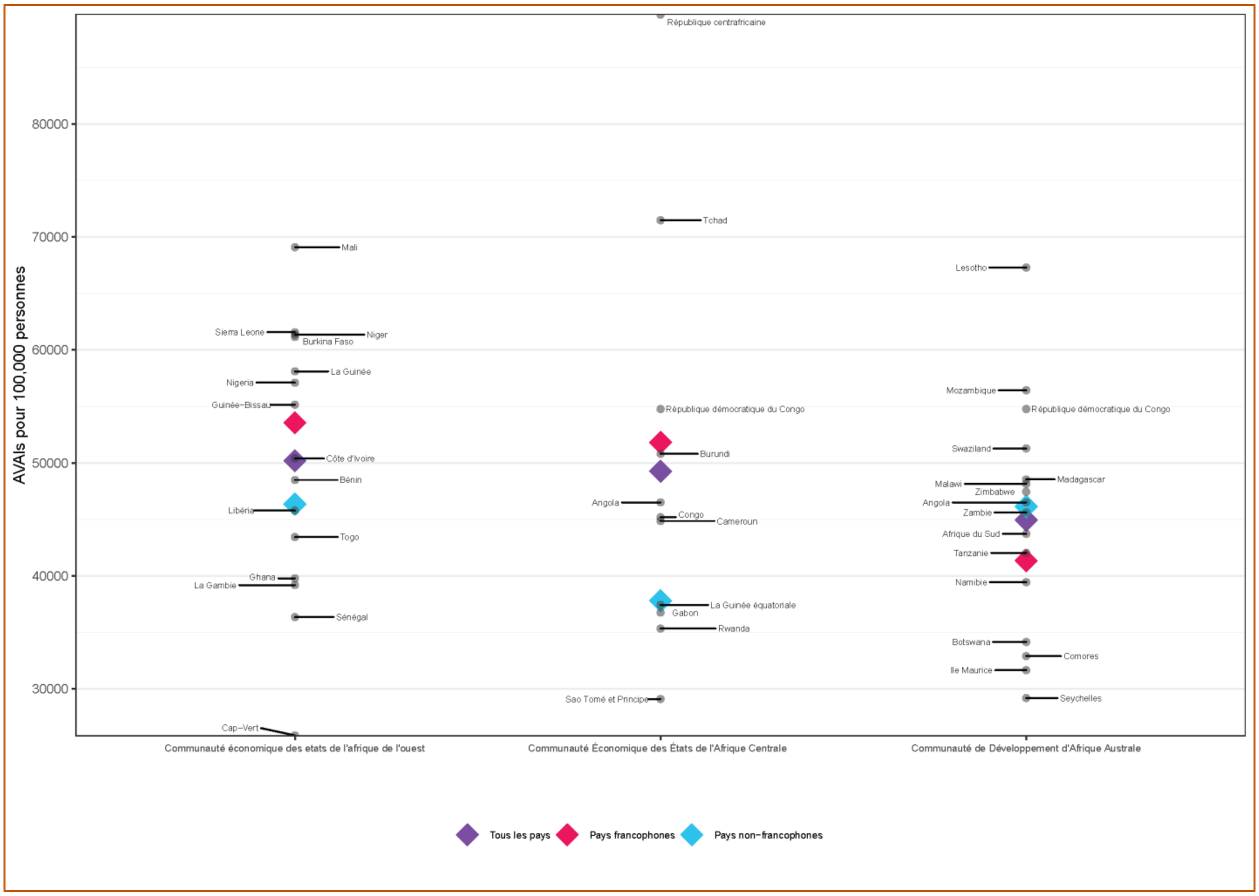

Figure 5: AVAIs dans les pays africains francophones et non francophones, au sein des trois communautés économiques Les pays francophones sont étiquetés à droite et les pays non-francophones sont étiquetés à gauche. AVAIs = années de vie ajustées de l'incapacité.

Globalement, lorsqu'elle est évaluée à l'aide des AVAIs, la charge la morbidité est plus élevée dans les pays africains francophones par rapport à leurs homologues non-francophones au sein de chaque communauté économique, sauf dans les pays de la Communauté de Développement de l'Afrique Australe où les Comores et les Seychelles font baisser le fardeau (figure 5). On observe le même schéma pour les AVPs (figure 6), mais pas pour les AVIs (annexe p 27), où le taux moyen est plus faible pour les pays francophones de la Communauté Économique des États de l'Afrique de l'Ouest et dans les pays de la Communauté de Développement de l'Afrique Australe.

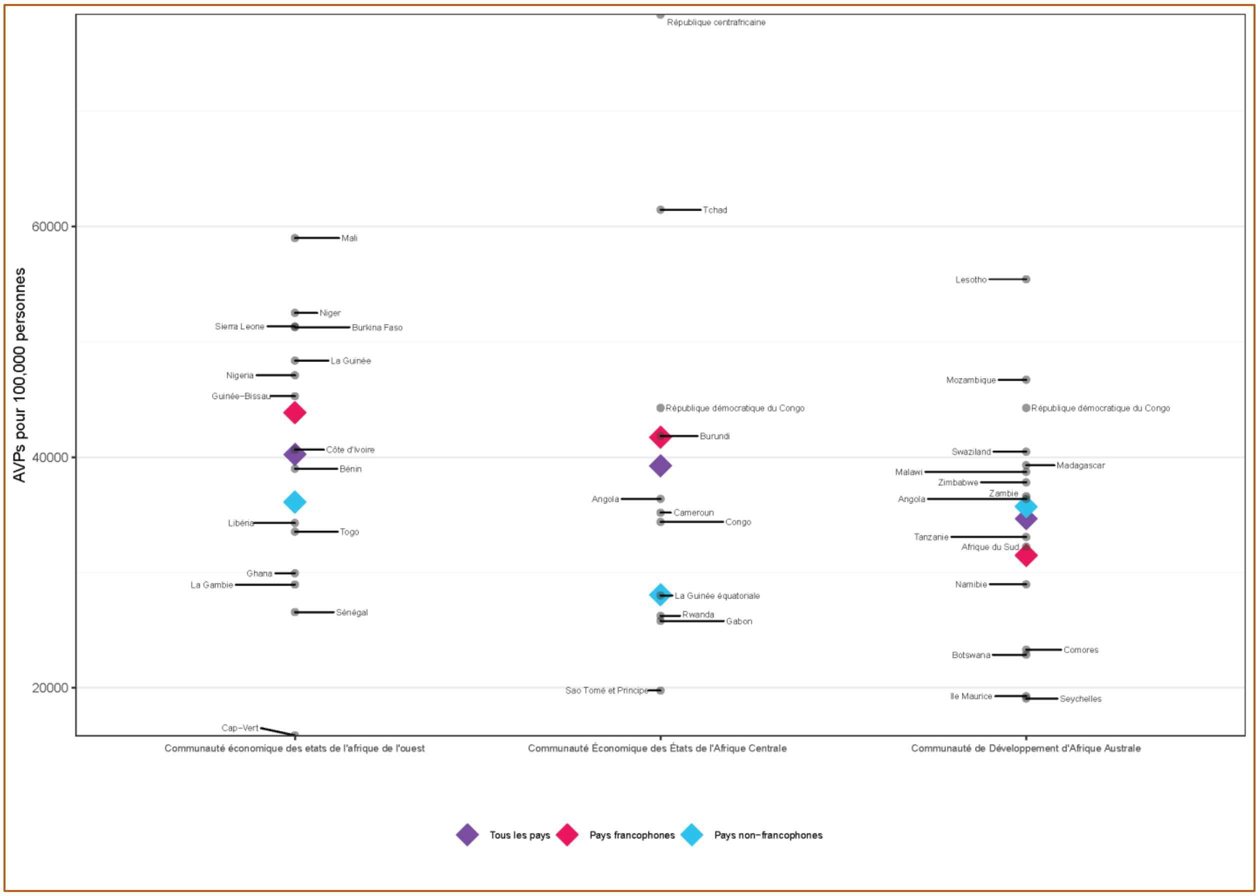

Figure 6: AVPs dans les pays africains francophones et non francophones, au sein des trois communautés économiques

Les pays francophones sont étiquetés à droite et les pays non-francophones sont étiquetés à gauche. AVPs = années de vie perdues

Discussion

Les pays de l'Afrique francophone ont réduit leur mortalité de plus de 45·3% depuis 1990. A notre connaissance, notre analyse est la première à mesurer la charge de morbidité en Afrique francophone et démontrer en même temps une décroissance de morbidité et de mortalité mais aussi un fardeau permanent des maladies transmissibles et un début de montée des maladies chroniques non-transmissibles. Notre étude montre également la charge de morbidité plus élevée dans les pays Africains francophones par rapport à leurs homologues non-francophones au sein de trois communautés économiques différentes.

Effectivement, et alors que les maladies transmissibles telles que les maladies diarrhéiques, la tuberculose et les infections des voies respiratoires inferieures pèsent toujours sur la santé de la population, viennent aussi s'ajouter les maladies chroniques non-transmissibles telles que la cardiopathie ischémique et les maladies cérebrovasculaires dont le rang en termes de cause de décès principales s'accroit. Toutefois, la charge de morbidité est dominée par la mortalité, mettant en lumière l'incapacité des systèmes de santé de l'Afrique francophone à gérer effectivement la morbidité de la population. L'index de la qualité et l'accès aux soins de santé [HAQI : Healthcare Access and Quality Index], index mesuré sur une échelle de 0 à 100, favorise cette hypothèse.[25] Pour illustrer cela, considérons la République Centrafricaine, dont le HAQI est de 18·6 —le plus bas du monde—et la Guinée Equatoriale, qui a un HAQI de 49·3. Alors que la Guinée équatoriale présente une moindre incidence de troubles néonatals que la République Centrafricaine —550·8 contre 626·6 nouveaux cas pour 100 000, respectivement—, les deux pays en ont la même prévalence —2275·5 contre 2303·8 cas prévalent pour 100 000. Cependant, la Guinée Equatoriale a un taux plus élevé d'AVIs —302·1 contre 252·8— que la République Centrafricaine.[12] Ainsi, les personnes atteintes de troubles néonatals ont tendance à vivre plus longtemps en Guinée Équatoriale et à mourir plus tôt en la République Centrafricaine. En Afrique francophone, seules les Seychelles dépassent le point médian de 50 en termes du HAQI, avec une valeur de 65·6.[25] Ainsi, un défi exceptionnel est à l'attente de l'Afrique francophone où les systèmes de santé sont loin du contrôle des maladies transmissibles et des troubles néonataux, et de faire face à la montée des maladies chroniques non-transmissibles. En outre, l'Afrique francophone présente une charge de maladie différente que celle dans les autres pays africains où le VIH/sida est la principales cause de décès, et où la majorité des causes principales de décès diminuent à un rythme plus rapide qu'en Afrique francophone.

La théorie de la transition sanitaire peut être mobilisée elle encore pour expliquer les résultats observés, relatifs aux changements dans les causes de décès.[26,27] Cependant il existe d´autres facteurs à garder en tête. D'abord, le revenu détermine la qualité des soins de santé qu´une population peut se procurer ainsi que son alimentation qui, à son tour, a une influence directe sur la mortalité par le biais de la malnutrition. La littérature existante fait état d'une baisse de la mortalité à mesure que le niveau de revenu du pays augmente.[28,29] Néanmoins, en dépit de leur niveau de revenu faible, les Comores et le Djibouti ont une des meilleures performances directement en-dessous des Seychelles, et même meilleures que certains pays à revenu intermédiaire supérieur tels que le Gabon et la Guinée Equatoriale. De plus, à côté de ces performances inattendues, on observe que le Cameroun, pourtant pays à revenu intermédiaire faible, présente des AVAIs bien supérieures à celles de pays à revenu faible tels que le Bénin, le Togo, le Madagascar, la République Démocratique du Congo, le Burundi, la Guinée et des pays à faible revenu cités plus haut. Ainsi, la pauvreté ne devrait-elle pas imposer un verdict incontestable. L'initiative *Salud Mesamerica*, initiative de la santé de la mère et de l'enfant en Amérique Centrale, cible les populations les plus pauvres de ces pays, et qui vivent dans des conditions pareilles ou pires que celles de l'Afrique.[30,31] Cette initiative de financement basé sur les résultats s'est montrée efficace dans des conditions ressemblant celles de l'Afrique, signalant de l'espoir pour toute population désirant améliorer sa santé.[32]

Deuxièmement, à quelques exceptions près, la surmortalité masculine est un phénomène démographique fréquemment observé dans les analyses démographiques. Nos résultats corroborent ce facteur car la charge globale, principalement due aux AVPs, est plus élevée pour les hommes dans la plupart des pays de cette étude.

Aussi, et malgré l'existence de stratégies efficaces pour le contrôle et le traitement du paludisme, de vaccins efficaces contre le rotavirus, la pneumonie, et la grippe,[33] et de traitements antibiotiques efficaces contre la tuberculose, le paludisme, les maladies diarrhéiques et IRIs demeurent parmi les cinq causes principales de décès en Afrique francophone. Les moustiquaires imprégnés d'insecticide peuvent prévenir le paludisme, mais l'accès à ces moustiquaires entre les populations à risque n'est supérieur à 80% en Afrique francophone qu'au Sénégal, au Mali et au Rwanda.[34] Cette couverture est même inférieure à 50% dans des pays comme le Gabon ou le Congo. Le vaccin conjugué contre le pneumocoque et le vaccin contre le rotavirus ne sont toujours pas introduits dans plusieurs des pays de l'Afrique francophone.[35] Le cas échéant, la couverture de ces deux vaccins n'est toujours pas optimale. En outre, il a été démontré que la couverture vaccinale effective est souvent inférieure à la couverture brute déclarée en raison de

nombreux problèmes avec les systèmes d'information sur la santé et les chaînes froides en Afrique et dans d'autres parties du monde.[36] En outre, la République Centrafricaine et le Congo sont parmi les 10 principaux pays de forte charge de tuberculose en fonction de la sévérité de la charge de morbidité (incidence par habitant).[37]

Classiquement, et comme partout en Afrique, la planification pour la santé en Afrique francophone s'est centralisée sur les maladies transmissibles. Or, avec le début de la montée des maladies chroniques non-transmissibles, surtout celles entraînées par les facteurs de risques comportementaux et diététiques, cette région devrait se rattraper en adoptant des interventions préventives. Précisément, la région devrait renforcer sa surveillance afin de suivre les tendances épidémiologiques et les progrès atteints, promouvoir la santé et supporter les comportements sains, améliorer la prestation et l'utilisation efficaces des services de soin et autres services préventifs basés sur des preuves, et lier les services de soins à des programmes communautaires pour améliorer et maintenir la prise en charge des maladies chroniques.[38]

Tel que nos résultats sur la charge observée et prévue le montrent, la plupart des pays, y compris les plus pauvres, ont performé mieux que prévu. Cela est surtout vrai en termes de mortalité, mais pas de morbidité. Dans la plupart des pays africains francophones, la charge observée approchait ou dépassait celle attendue en termes d'AVIs (annexe pp 28–29), soulignant la faiblesse persistante dans la gestion de la maladie. En effet, à l'exception des Comores, Djibouti, Gabon, Rwanda et les Seychelles, les pays francophones d'Afrique sont parmi ceux du plus bas quintile, 22 à 45, de l'indice de la couverture universelle en terme de santé qui varie entre 22 et 86.[39] En termes du HAQI, 18 (86%) des 21 pays africains francophones se situent dans le quintile inférieur, à l'exception du Gabon, de la Guinée Equatoriale et des Seychelles, alors que seulement 11 (55%) de leurs 20 homologues non-francophones dans les trois communautés économiques étudiées se situent dans ce même quintile. Depuis 1990, la part de l'aide publique au développement pour la santé consacrée aux approches sectorielles et le renforcement des systèmes de santé a constamment diminué en Afrique francophone, au point où elle constituait moins que 15% en 2015, dans un pays donné, sauf pour les Seychelles, la Guinée équatoriale et le Bénin.[40] Dans l'ensemble, moins de 6% de l'aide publique au développement pour la santé a été consacrée en 2015 aux approches sectorielles et au renforcement des systèmes de santé dans les 21 pays combinés. Le HAQI a augmenté avec la hausse du ratio de l'aide au développement pour la santé consacrée aux approches sectorielles et au renforcement des systèmes de santé reçue entre 1990 et 2016 par rapport à la taille de la population du pays en 2016·[41] Par exemple, ce ratio a dépassé 146 dollars

Américains par personne aux Seychelles mais est tombé en dessous de 7 dollars par personne pour la République Démocratique du Congo.

Notre étude présente plusieurs limites malgré ses avancées méthodologiques. Une section détaillant toutes les limites de l'étude CMM est publiée ailleurs.[14] Brièvement, et comme toute analyse épidémiologique, la précision des estimations dépend largement de la disponibilité des sources de données et de la quantité de données pour une période donnée. En l'absence de données spécifiques, les estimations dépendent des covariables et du modèle statistique utilisé. En outre, le processus de modélisation de la CMM est fondé sur les causes et non sur les lieux. Cela signifie que la hiérarchie complète des lieux (c'est-à-dire tous les lieux modélisés pour la CMM) est modélisée de manière unique pour chaque cause. La stratégie de modélisation de la CMM permet aux endroits où les données sont plus rares (par cause, âge et sexe) de tirer parti des endroits plus riches en données dans la région et la super-région CMM afin d'éclairer les estimations alors que, dans le même modèle, les endroits riches en données sont moins dépendants d'autres données. Un classement par étoiles des causes de décès pour chaque pays africain francophone est présenté dans l'annexe (p 30). Le système de classement par étoiles est sur la base des données de l'état civil et produit un classement pour chaque pays de 1 à 5 étoiles.[42] La plupart des pays Africains francophones ont une note de 0 alors qu'un peu d'entre eux en ont 1. Cela suggère que tous les lieux ont dû tirer parti considérablement d'autres lieux de leur région et de leur super-région dans la hiérarchie des lieux de la CMM. Pour ces raisons, les estimations de la CMM sont produites avec des intervalles d'incertitude pour aider les lecteurs à mieux peser et contextualiser chaque estimation produite. En outre, la qualité des données disponibles varie parfois en raison de biais d'échantillonnage et de non-échantillonnage pour le même pays, ce qui augmente l'incertitude pour comparer la même mesure dans plusieurs pays.

L'Afrique francophone porte une charge disproportionnée de morbidité, un fait probablement dû à la faiblesse des systèmes de santé et services de soins. Pour faire face à cette charge de morbidité, l'Afrique francophone devrait définir ses priorités et investir plus de ressources dans le renforcement des systèmes de santé, l'offre de soins en qualité et en quantité, particulièrement dans les zones rurales et reculées. Les trois communautés économiques peuvent se fixer des objectifs communs pour réduire les disparités intracommunautaires en matière de santé grâce à différents modèles d'assistance dans un environnement réfléchi de connaissances et à des forums d'échange de connaissances. Cette région pourrait aussi être priorisée en termes d'assistances technique et financière ciblées autant sur le renforcement des systèmes de santé et des services des soins que sur des investissements démographiques. Plusieurs modes de

financement peuvent être employés, en particulier le financement basé sur les résultats dans un cadre de redevabilité rigide, et selon une approche régionale différente de celle suivie classiquement et qui consiste à cibler chaque pays seul.[30,40]

Liste bibliographique

1 Organisation internationale de la Francophonie. Welcome to the International Organisation of La Francophonie's official website. http://mediatheque.francophonie.org/English.html (accessed July 9, 2019).

2 World Atlas. French speaking countries in Africa. June 28, 2018. https://www.worldatlas.com/articles/french-speaking-countries-inafrica. html (accessed Nov 7, 2019).

3 Worldometer. World population clock: 7·6 billion people. http://www.worldometers.info/world-population (accessed June 8, 2018).

4 Kyu HH, Abate D, Abate KH, et al. Global, regional, and national disability-adjusted life-years (DALYs) for 359 diseases and injuries and healthy life expectancy (HALE) for 195 countries and territories, 1990–2017: a systematic analysis for the Global Burden of Disease Study 2017. *Lancet* 2018; **392:** 1859–922.

5 Santosa A, Wall S, Fottrell E, Högberg U, Byass P. The development and experience of epidemiological transition theory over four decades: a systematic review. *Glob Health Action* 2014; **7:** 23574.

6 Rabarison KM, Bish CL, Massoudi MS, Giles WH. Economic evaluation enhances public health decision making. *Front Public Health* 2015; **3:** 164.

7 The Lancet Public Health. Two days in Abidjan: finding the voice of francophone Africa. *Lancet Public Health* 2017; **2:** e56.

8 Baussano I, Brzoska P, Fedeli U, Larouche C, Razum O, Fung IC-H. Does language matter? A case study of epidemiological and public health journals, databases and professional education in French, German and Italian. *Emerg Themes Epidemiol* 2008; **5:** 16.

9 Defor S, Kwamie A, Agyepong IA. Understanding the state of health policy and systems research in West Africa and capacity strengthening needs: scoping of peer-reviewed publications trends and patterns 1990–2015. *Health Res Policy Syst* 2017; **15** (suppl 1)**:** 55.

10 Benie-Bi J, Cambon L, Grimaud O, Kivits J, Alla F. Health needs and public health functions addressed in scientific publications in Francophone sub-Saharan Africa. *Public Health* 2013; **127:** 860–66.

11 Murray CJL, Lopez AD. Measuring global health: motivation and evolution of the Global Burden of Disease Study. *Lancet* 2017; **390:** 1460–64.

12 Institute for Health Metrics and Evaluation. GBD Compare. IHME Viz Hub. http://vizhub.healthdata.org/gbd-compare (accessed June 8, 2018).

13 World Bank. World development report 1993: investing in health. New York, NY: Oxford University Press, 1993.

14 GBD 2016 Mortality Collaborators. Global, regional, and national under-5 mortality, adult mortality, age-specific mortality, and life expectancy, 1970–2016: a systematic analysis for the Global Burden of Disease Study 2016. *Lancet* 2017; **390:** 1084–150.

15 Vos T, Abajobir AA, Abate KH, et al. Global, regional, and national incidence, prevalence, and years lived with disability for 328 diseases and injuries for 195 countries, 1990–2016: a systematic analysis for the Global Burden of Disease Study 2016. *Lancet* 2017; **390:** 1211–59.

16 Stevens GA, Alkema L, Black RE, et al. Guidelines for Accurate and Transparent Health Estimates Reporting: the GATHER statement. *Lancet* 2016; **388:** e19–23.

17 GBD 2017 DALYs and HALE Collaborators. Global, regional, and national disability-adjusted life-years (DALYs) for 359 diseases and injuries and healthy life expectancy (HALE) for 195 countries and territories, 1990–2017: a systematic analysis for the Global Burden of Disease Study 2017. *Lancet* 2018; **392:** 1859–922.

18 Murray CJL, Ezzati M, Flaxman AD, et al. GBD 2010: design, definitions, and metrics. *Lancet* 2012; **380:** 2063–66.

19 GBD 2016 DALYs and HALE Collaborators. Global, regional, and national disability-adjusted life-years (DALYs) for 333 diseases and injuries and healthy life expectancy (HALE) for 195 countries and territories, 1990–2016: a systematic analysis for the Global Burden of Disease Study 2016. *Lancet* 2017; **390:** 1260–344.

20 GBD 2016 Disease and Injury Incidence and Prevalence Collaborators. Global, regional, and national incidence, prevalence, and years lived with disability for 328 diseases and injuries for 195 countries, 1990–2016: a systematic analysis for the Global Burden of Disease Study 2016. *Lancet* 2017; **390:** 1211–59.

21 GBD 2016 Risk Factors Collaborators. Global, regional, and national comparative risk assessment of 84 behavioural, environmental and occupational, and metabolic risks or clusters of risks, 1990–2016: a systematic analysis for the Global Burden of Disease Study 2016. *Lancet* 2017; **390:** 1345–422.

22 Wang H, Naghavi M, Allen C, et al. Global, regional, and national life expectancy, all-cause mortality, and cause-specific mortality for 249 causes of death, 1980–2015: a systematic analysis for the Global Burden of Disease Study 2015. *Lancet* 2016; **388:** 1459–544.

23 Dicker D, Nguyen G, Abate D, et al. Global, regional, and national age-sex-specific mortality and life expectancy, 1950–2017: a systematic analysis for the Global Burden of Disease Study 2017. *Lancet* 2018; **392:** 1684–735.

24 GBD 2016 Causes of Death Collaborators. Global, regional, and national age-sex specific mortality for 264 causes of death, 1980–2016: a systematic analysis for the Global Burden of Disease Study 2016. *Lancet* 2017; **390:** 1151–210.

25 Fullman N, Yearwood J, Abay SM, et al. Measuring performance on the Healthcare Access and Quality Index for 195 countries and territories and selected subnational locations: a systematic analysis from the Global Burden of Disease Study 2016. *Lancet* 2018; **391:** 2236–71.

26 Vallin J, Meslé F. Will life expectancy increase indefinitely by three months every year? *Popul Soc Paris* 2010; **473:** 1–4.

27 Frenk J, Bobadilla JL, Stern C, Frejka T, Lozano R. Elements for a theory of the health transition. *Health Transit Rev* 1991; **1:** 21–38.

28 O'Hare B, Makuta I, Chiwaula L, Bar-Zeev N. Income and child mortality in developing countries: a systematic review and metaanalysis. *J R Soc Med* 2013; **106:** 408–14.

29 Buor D, Bream K. An analysis of the determinants of maternal mortality in sub-Saharan Africa. *J Womens Health (Larchmt)* 2004; **13:** 926–38.

30 Gutiérrez JP, Téllez-Rojo MM, Torres P, Romero M, Bertozzi SM, Subgrupo de Evaluación de Impacto. Mixed design for the evaluation of the Mesoamerica Health 2015 initiative. *Salud Publica Mex* 2011; **53** (suppl 3)**:** S386–95 (in Spanish).

31 Mokdad AH, Colson KE, Zúñiga-Brenes P, et al. Salud Mesoamérica 2015 Initiative: design, implementation, and baseline findings. *Popul Health Metr* 2015; **13:** 3.

32 Mokdad AH, Palmisano EB, Zúñiga-Brenes P, et al. Supply-side interventions to improve health: findings from the Salud Mesoamérica Initiative. *PLoS One* 2018; **13:** e0195292.

33 Whitney CG, Harper SA. Lower respiratory tract infections: prevention using vaccines. *Infect Dis Clin North Am* 2004; **18:** 899–917.

34 WHO. World malaria report 2017. Geneva: World Health Organization, 2017.

35 WHO. Data, statistics and graphics. http://www.who.int/ immunization/monitoring_surveillance/data/en (accessed June 8, 2018).

36 Mokdad AH, Gagnier MC, Colson KE, et al. Missed opportunities for measles, mumps, and rubella (MMR) immunization in Mesoamerica: potential impact on coverage and days at risk. *PLoS One* 2015; **10:** e0139680.

37 WHO. Global tuberculosis report 2018. Geneva: World Health Organization, 2018.

38 National Center for Chronic Disease Prevention and Health Promotion, Centers for Disease Control and Prevention. The four domains of chronic disease Prevention. 2015. https://stacks.cdc.gov/view/cdc/27508 (accessed Jan 30, 2020).

39 WHO, International Bank for Reconstruction and Development/ World Bank. Tracking universal health coverage: 2017 global monitoring report. Geneva: World Health Organization and International Bank for Reconstruction and Development/World Bank, 2017.

40 Dieleman JL, Haakenstad A, Micah A, et al. Spending on health and HIV/AIDS: domestic health spending and development assistance in 188 countries, 1995–2015. *Lancet* 2018; **391:** 1799–829.

41 Chang AY, Cowling K, Micah AE, et al. Past, present, and future of global health financing: a review of development assistance, government, out-of-pocket, and other private spending on health for 195 countries, 1995–2050. *Lancet* 2019; **393:** 2233–60.

42 Roth GA, Abate D, Abate KH, et al. Global, regional, and national age-sex-specific mortality for 282 causes of death in 195 countries and territories, 1980–2017: a systematic analysis for the Global Burden of Disease Study 2017. *Lancet* 2018; **392:** 1736–88.

43 El Bcheraoui C, Palmisano EB, Dansereau E, et al. Healthy competition drives success in results-based aid: Lessons from the Salud Mesoamérica Initiative. *PLoS One* 2017; **12:** e0187107.

Annexe

Tableau 1: Répartition des pays africains francophones (marqués d'un astérisque), avec leurs homologues non-francophones, par groupes économiques, et indice sociodémographique (ISD) 3

Tableau 2: Niveau, causes, et ordre des causes de décès........ 4

Figure 1: Principales causes de décès et leur évolution, tous âges, Communauté Economique des États de l'Afrique de l'Ouest, pays francophones, 1990-2017........ 16

Figure 2: Principales causes de décès et leur évolution, tous âges, Communauté Economique des États de l'Afrique de l'Ouest, pays non-francophones, 1990-2017........ 17

Figure 3: Principales causes de décès et leur évolution, tous âges, Communauté Economique des États d'Afrique Centrale, pays francophones, 1990-2017 18

Figure 4: Principales causes de décès et leur évolution, tous âges, Communauté Economique des États d'Afrique Centrale, pays non-francophones, 1990-2017........ 19

Figure 5: Principales causes de décès et leur évolution, tous âges, Communauté de Développement de l'Afrique Australe, pays francophones, 1990-2017 20

Figure 6: Principales causes de décès et leur évolution, tous âges, Communauté de Développement de l'Afrique Australe, pays non-francophones, 1990-2017........ 21

Figure 7: Principales causes des années de vie perdues et leur évolution, tous âges, Afrique francophone, 1990-2017........ 22

Figure 8: Principales causes des années de vie perdues et leur évolution, tous âges, Afrique non-francophone, 1990-2017........ 23

Figure 9: Années de vie ajustées de l'incapacité (AVAIs) pour 100 000 habitants, et intervalles d'incertitude (segments noirs), tous âges, par sexe et par pays, Afrique francophone, 2017 24

Figure 10: Facteurs de risque par années de vie ajustées de l'incapacité, Afrique francophone, 2017........ 25

Figure 11: Décomposition du changement parmi les 30 principales causes de décès dues à la croissance démographique, au vieillissement de la population et au taux de mortalité par âge et par cause, Afrique francophone, 1990-2017. Par exemple, dans le cas de la maladie d'Alzheimer et d'autres démences, la mortalité par âge et par cause a contribué à une légère diminution des AVAIs, mais la croissance démographique et le vieillissement de la population ont entraîné une forte augmentation, ce qui fait que le pourcentage total de changement est d'environ 130 %........ 26

Figure 12: Années vécues avec une incapacité dans les pays francophones (étiquetés à droite) et non-francophones (étiquetés à gauche), au sein des trois communautés économiques....27

Tableau 3: Décès, années de vie perdues (AVPs), années vécues avec une incapacité (AVIs) et années de vie ajustées de l'incapacité (AVAIs) en 1990 et 2017, et espérance de vie, AVAIs prévues, AVIs prévues, indice sociodémographique (ISD) en 2017 et évolution en pourcentage de l'ISD entre 1990 et 2017....28

Tableau 4: Classement par étoiles des causes de décès pour les systèmes d'enregistrement de l'état civil, Afrique francophone....30

Tableau 1: Répartition des pays africains francophones (marqués d'un astérisque), avec leurs homologues non-francophones, par groupes économiques, et indice sociodémographique (ISD)

**Communauté économique des États de l'Afrique de l'Ouest (ISD)**	**Communauté économique des États de l'Afrique centrale (ISD)**	**Communauté de développement de l'Afrique australe (ISD)**
Le Benin* (0.37)	Le Burundi* (0.31)	Les Comores* (0.43)
Le Burkina Faso* (0.28)	La République Centrafricaine* (0.33)	La République Démocratique du Congo * (0.36)
La Cote d'Ivoire* (0.41)	Le Cameroun* (0.48)	Le Madagascar* (0.33)
La Guinée* (0.32)	Le Tchad* (0.25)	Les Seychelles* (0.69)
Guinée Bissau* (0.35)	Le Congo* (0.57)	Angola (0.46)
Le Mali* (0.27)	La République Démocratique du Congo* (0.36)	Le Botswana (0.66)
Le Niger* (0.19)	La Guinée Equatoriale* (0.63)	Le Lesotho (0.49)
Le Sénégal* (0.37)	Le Gabon* (0.65)	Malawi (0.35)
Le Togo* (0.41)	Le Rwanda* (0.41)	L'île Maurice (0.72)
Le Cap Vert (0.55)	Angola (0.46)	Le Mozambique (0.34)
Le Ghana (0.54)	Sao Tomé et Principe (0.49)	La Namibie (0.62)
Le Libéria (0.33)		L'Afrique du Sud (0.68)
Le Nigéria (0.49)		Le Swaziland (0.58)
La Sierra Leone (0.36)		La Tanzanie (0.41)
La Gambie (0.40)		La Xambie (0.47)
		Le Zimbabwe (0.46)

Tableau 2**:** Niveau, causes, et ordre des causes de décès

**Niveau**	**Causes de décès**	**Ordre des causes**
0	Toutes causes	Total
1	Maladies transmissibles, maternelles, néonatales et nutritionnelles	A
3	Tuberculose	A.2.1
3	VIH/SIDA	A.1.1
4	VIH/SIDA résultant en d'autres maladies	A.1.1.4
3	Maladies diarrhéiques	A.3.1
4	Fièvre typhoïde	A.3.2.1
4	Fièvre paratyphoïde	A.3.2.2
3	Autres maladies infectieuses intestinales	A.3.5
3	Infections des voies respiratoires inférieures	A.2.2
3	Infections des voies respiratoires supérieures	A.2.3
3	Otite moyenne	A.2.4
3	Méningite	A.5.1
4	Méningite à pneumocoque	A.5.1.1
4	Méningite de type B à H influenzae	A.5.1.2
4	Méningite à méningocoques	A.5.1.3
4	Autres méningites	A.5.1.4
3	Encéphalite	A.5.2
3	Diphtérie	A.5.3
3	Coqueluche	A.5.4
3	Tétanos	A.5.5
3	Rougeole	A.5.6
3	Varicelle et Zona	A.5.7
2	Paludisme et maladies tropicales négligées	A.4
3	Paludisme	A.4.1
3	Maladie de Chagas	A.4.2
3	Leishmaniose	A.4.3
4	Leishmaniose viscérale	A.4.3.1
4	Leishmaniose cutanée et cutanéo-muqueuse	A.4.3.2

3	Trypanosomiase africaine	A.4.4
3	Schistosomiase	A.4.5
3	Cysticercose	A.4.6
3	Échinococcose kystique	A.4.7
3	Filariose lymphatique	A.4.8
3	Onchocercose	A.4.9
3	Trachome	A.4.10
3	Dengue	A.4.11
3	Fièvre jaune	A.4.12
3	Rage	A.4.13
3	Nématodoses intestinales	A.4.14
4	Ascaridiose	A.4.14.1
4	Trichocéphalose	A.4.14.2
4	Ankylostomiase	A.4.14.3
3	Trematodiases toxi-infections alimentaires	A.4.15
3	Autres maladies tropicales négligées	A.4.20
3	Troubles maternels	A.6.1
4	Hémorragie maternelle	A.6.1.1
4	Septicémie maternelle et autres infections maternelles	A.6.1.2
4	Troubles hypertensifs maternelle	A.6.1.3
4	Accouchement obstrué et rupture utérine	A.6.1.4
4	Grossesse extra-utérine	A.6.1.6
4	Décès maternels indirects	A.6.1.7
4	Décès maternels tardifs	A.6.1.8
4	Autres troubles maternels	A.6.1.10
3	Troubles néonatals	A.6.2
4	Prématurité néonatale	A.6.2.1
4	Encéphalopathie néonatale due à l'asphyxie à la naissance et au traumatisme	A.6.2.2
4	Septicémie néonatale et autres infections néonatales	A.6.2.3
4	Maladie hémolytique et autre ictère néonatal	A.6.2.4
4	Autres troubles néonatals	A.6.2.5

2	Carences nutritionnelles	A.7
3	Malnutrition protéino-énergétique	A.7.1
3	Carence en iode	A.7.2
3	Carence en vitamine A	A.7.3
3	Carence en fer alimentaire	A.7.4
3	Autres carences nutritionnelles	A.7.5
3	Maladies sexuellement transmissibles, à l'exclusion du VIH	A.1.2
4	Syphilis	A.1.2.1
4	Infection à Chlamydia trachomatis	A.1.2.2
4	Infection gonococcique	A.1.2.3
4	Trichomonase	A.1.2.4
4	Herpès génital	A.1.2.5
4	Autres infections sexuellement transmissibles	A.1.2.6
3	Hépatite aiguë	A.5.8
4	Hépatite A aiguë	A.5.8.1
4	Hépatite B aiguë	A.5.8.2
4	Hépatite C aiguë	A.5.8.3
4	Hépatite E aiguë	A.5.8.4
3	Lèpre	A.4.16
3	Autres maladies infectieuses non préciséEs	A.5.9
1	Maladies non transmissibles	B
2	Néoplasmes	B.1
3	Cancer de l'oesophage	B.1.4
3	Cancer de l'estomac	B.1.5
3	Cancer du foie	B.1.7
4	Cancer du foie dû à l'hépatite B	B.1.7.1
4	Cancer du foie dû à l'hépatite C	B.1.7.2
4	Cancer du foie dû à la consommation d'alcool	B.1.7.3
4	Cancer du foie dû à d'autres causes	B.1.7.5
3	Cancer du larynx	B.1.10
3	Cancer de la trachée, des bronches et des poumons	B.1.11

3	Cancer du sein	B.1.14
3	Cancer du col utérin	B.1.15
3	Cancer de l’utérus	B.1.16
3	Cancer de la prostate	B.1.18
3	Cancer du côlon et du rectum	B.1.6
3	Cancer de la lèvre et de la cavité buccale	B.1.1
3	Cancer du nasopharynx	B.1.2
3	Autres cancers du pharynx	B.1.3
3	Vésicule biliaire et le cancer des voies biliaires	B.1.8
3	Cancer du pancréas	B.1.9
3	Tumeur maligne de la peau	B.1.12
3	Cancer de la peau non mélanique	B.1.13
3	Cancer de l’ovaire	B.1.17
3	Cancer du testicule	B.1.19
3	Cancer du rein	B.1.20
3	Cancer de la vessie	B.1.21
3	Cancer du cerveau et du système nerveux	B.1.22
3	Cancer de la thyroïde	B.1.23
3	Mésothéliome	B.1.24
3	Lymphome de Hodgkin	B.1.25
3	Lymphome non hodgkinien	B.1.26
3	Myélome multiple	B.1.27
3	Leucémie	B.1.28
3	Autres tumeurs malignes	B.1.29
3	Autres néoplasmes	B.1.30
2	Maladies cardiovasculaires	B.2
3	Cardiopathie rhumatismale	B.2.1
3	La cardiopathie ischémique	B.2.2
3	Accident vasculaire cérébral	B.2.3
4	Accident vasculaire cérébral ischémique	B.2.3.1
4	Hémorragie intracérébrale	B.2.3.2

4	Hémorragie sous-arachnoïdienne	B.2.3.3
3	Cardiopathie hypertensive	B.2.4
3	Myocardite et cardiomyopathie	B.2.6
3	Fibrillation et flutter auriculaires	B.2.7
3	Anévrisme de l’aorte	B.2.8
3	Maladie artérielle périphérique	B.2.9
3	Endocardite	B.2.10
3	Cardiopathie valvulaire non-rhumatismale	B.2.5
3	Autres maladies cardiovasculaires et circulatoires	B.2.11
2	Maladies respiratoires chroniques	B.3
3	Maladie pulmonaire obstructive chronique	B.3.1
3	Pneumoconiose	B.3.2
4	Silicose	B.3.2.1
4	Asbestose	B.3.2.2
4	Pneumoconiose des mineurs de charbon	B.3.2.3
4	Autres pneumoconioses	B.3.2.4
3	Asthme	B.3.3
3	Pneumopathie interstitielle et Sarcoïdose pulmonaire	B.3.4
3	Autres maladies respiratoires chroniques	B.3.5
3	Cirrhose et autres maladies chroniques du foie	B.4.1
4	Cirrhose et autres maladies chroniques du foie dues à l’hépatite B	B.4.1.1
4	Cirrhose et autres maladies chroniques du foie dues à l’hépatite C	B.4.1.2
4	Cirrhose et autres maladies chroniques du foie dues à l'utilisation de l’alcool	B.4.1.3
4	Cirrhose et autres maladies chroniques du foie dues à d’autres causes	B.4.1.5
2	Maladies digestives	B.4
4	Ulcère gastro-duodénal	B.4.2.1
4	Gastrite et duodénite	B.4.2.2
3	Appendicite	B.4.3
3	Iléus paralytique et occlusion intestinale	B.4.4
3	Hernie inguinale, fémorale et abdominale	B.4.5
3	Maladies inflammatoires de l’intestin	B.4.6

3	Troubles intestinaux vasculaires	B.4.7
3	Maladies de la vésicule biliaire et des voies biliaires	B.4.8
3	Pancréatite	B.4.9
4	Le reflux gastro-œsophagien	B.4.2.3
3	Autres maladies digestives	B.4.10
2	Troubles neurologiques	B.5
3	La maladie d'Alzheimer et autres démences	B.5.1
3	Maladie de Parkinson	B.5.2
3	Épilepsie	B.5.3
3	Sclérose en plaques	B.5.4
4	Migraine	B.5.6.1
4	Céphalée de tension	B.5.6.2
3	Maladies du motoneurone	B.5.5
3	Autres troubles neurologiques	B.5.7
2	Troubles mentaux	B.6
3	Schizophrénie	B.6.1
3	Troubles liés à l'utilisation d'alcool	B.7.1
3	Troubles de la consommation de drogue	B.7.2
4	Troubles liés à l'utilisation des opiacés	B.7.2.1
4	Troubles liés à l'utilisation de cocaïne	B.7.2.2
4	Troubles liés à l'utilisation d'amphétamine	B.7.2.3
4	Troubles liés à l'utilisation de cannabis	B.7.2.4
4	Autres troubles liés à l'utilisation de drogues	B.7.2.5
3	Troubles dépressifs	B.6.2
4	Trouble dépressif majeur	B.6.2.1
4	Dysthymie	B.6.2.2
3	Trouble bipolaire	B.6.3
3	Troubles anxieux	B.6.4
3	Troubles de l'alimentation	B.6.5
4	Anorexie mentale	B.6.5.1
4	Boulimie nerveuse	B.6.5.2

3	Troubles du spectre autistique	B.6.6
3	Trouble du déficit de l'attention/hyperactivité	B.6.7
3	Trouble des conduites	B.6.8
3	Déficience intellectuelle du développement idiopathique	B.6.9
3	Autres troubles mentaux	B.6.10
3	Diabète sucré	B.8.1
3	Glomérulonéphrite aiguë	B.8.3
3	Insuffisance rénale chronique	B.8.2
4	Maladie rénale chronique dûe au l'hypertension	B.8.2.3
4	Maladie rénale chronique dûe à la glomérulonéphrite	B.8.2.4
4	Maladie rénale chronique dûe à d'causes autres et à des causes non précisées	B.8.2.5
3	Maladies urinaires et infertilité masculine	B.12.2
4	Infections des voies urinaires	B.12.2.1
4	Lithiase urinaire	B.12.2.2
4	Hyperplasie bénigne de la prostate	B.12.2.3
4	Infertilité masculine	B.12.2.4
4	Autres maladies urinaires	B.12.2.5
3	Maladies gynécologiques	B.12.3
4	Fibromes utérins	B.12.3.1
4	Syndrome des ovaires polykystiques	B.12.3.2
4	Infertilité féminine	B.12.3.3
4	Endométriose	B.12.3.4
4	Prolapsus génital	B.12.3.5
4	Syndrome prémenstruel	B.12.3.6
4	Autres maladies gynécologiques	B.12.3.7
3	Hémoglobinopathies et anémies hémolytiques	B.12.4
4	Thalassémies	B.12.4.1
4	Troubles de la drépanocytose	B.12.4.3
4	Déficit en G6PD	B.12.4.5
4	Autres hémoglobinopathies et les anémies hémolytiques	B.12.4.7
3	Troubles endocriniens, métaboliques, sanguins, et immunitaires	B.12.5

2	Troubles musculo-squelettiques	B.11
3	La polyarthrite rhumatoïde	B.11.1
3	Arthrose	B.11.2
3	Lombalgie	B.11.3
3	Douleur au cou	B.11.4
3	Goutte	B.11.5
3	Autres troubles musculo-squelettiques	B.11.6
2	Autres maladies non transmissibles	B.12
3	Malformations congénitales	B.12.1
4	Anomalies du tube neural	B.12.1.1
4	Anomalies cardiaques congénitales	B.12.1.2
4	Fentes orofaciale	B.12.1.3
4	Syndrome de Down	B.12.1.4
4	Syndrome de Turner	B.12.1.5
4	Syndrome de Klinefelter	B.12.1.6
4	Autres anomalies chromosomiques	B.12.1.7
4	Anomalies congénitales musculo-squelettiques et des membres	B.12.1.8
4	Anomalies urogénitales congénitales	B.12.1.9
4	Anomalies digestives congénitales	B.12.1.10
4	Autres malformations congénitales	B.12.1.11
2	Maladies cutanées et sous-cutanées	B.9
3	Dermatite	B.9.1
3	Psoriasis	B.9.2
4	Cellulite	B.9.3.1
4	Pyodermite	B.9.3.2
3	Gale	B.9.4
3	Maladies fongiques de la peau	B.9.5
3	Dermatoses virales	B.9.6
3	L'acné vulgaire	B.9.7
3	La pelade	B.9.8
3	Prurit	B.9.9

3	Urticaire	B.9.10
3	Ulcère de decubitus	B.9.11
3	Autres maladies cutanées et sous-cutanées	B.9.12
2	Maladies des organes des sens	B.10
4	Glaucome	B.10.1.1
4	Cataracte	B.10.1.2
4	Dégénérescence maculaire liée à l'âge	B.10.1.3
3	Perte d'audition liée à l'âge et à d'autres causes	B.10.2
4	Autres pertes de vision	B.10.1.6
3	Autres maladies des organes des sens	B.10.3
3	Troubles de l'oralité	B.12.6
4	Carie des dents de lait	B.12.6.1
4	Carie des dents permanentes	B.12.6.2
4	Maladies parodontales	B.12.6.3
4	Édentation et perte sévère des dents	B.12.6.4
4	Autres troubles oraux	B.12.6.5
3	Mort subite du nourrisson	B.12.7
1	Blessures	C
2	Accidents de transport	C.1
3	Accidents de la route	C.1.1
4	Accidents de piétons	C.1.1.1
4	Accidents de cyclistes	C.1.1.2
4	Accidents de motocycliste	C.1.1.3
4	Accidents de véhicules automobiles	C.1.1.4
4	Autres accidents de la route	C.1.1.5
3	Autres accidents de transport	C.1.2
2	Blessures accidentelles	C.2
3	Chutes	C.2.1
3	Noyade	C.2.2
3	Feu, chaleur et substances chaudes	C.2.3
3	Empoisonnements	C.2.4

4	Empoisonnement par le monoxyde de carbone	C.2.4.1
4	Empoisonnement par d'autres moyens	C.2.4.2
3	Exposition à des forces mécaniques	C.2.5
4	Blessures accidentelles causées par une arme à feu	C.2.5.1
4	Autres expositions à des forces mécaniques	C.2.5.2
3	Effets indésirables du traitement médical	C.2.6
3	Contact avec des animaux	C.2.7
4	Contact avec des animaux venimeux	C.2.7.1
4	Contact avec des animaux non venimeux	C.2.7.2
3	Corps étranger	C.2.8
4	Aspiration pulmonaire et corps étranger dans les voies respiratoires	C.2.8.1
4	Corps étranger dans les yeux	C.2.8.2
4	Corps étranger dans d'autres parties du corps	C.2.8.3
3	Autres blessures accidentelles	C.2.11
2	Violence interpersonnelle et automutilation	C.3
3	Automutilation	C.3.1
4	Automutilation par arme à feu	C.3.1.1
4	Automutilation autrement spécifiée	C.3.1.2
3	Violence interpersonnelle	C.3.2
4	Violence physique par arme à feu	C.3.2.1
4	Violence physique par objet tranchant	C.3.2.2
4	Violence physique par d'autres moyens	C.3.2.4
3	Exposition aux forces de la nature	C.2.10
4	Décès maternels aggravée par le VIH/SIDA	A.6.1.9
4	Trait thalassémique	B.12.4.2
4	Trait drépanocytaire	B.12.4.4
4	Trait de G6PD	B.12.4.6
3	Exposition à la chaleur et au froid environnementaux	C.2.9
3	Ebola	A.4.17
4	Leucémie lymphoïde aiguë	B.1.28.1
4	Leucémie lymphoïde chronique	B.1.28.2

4	Leucémie myéloïde aiguë	B.1.28.3
4	Leucémie myéloïde chronique	B.1.28.4
4	Cancer de la peau non mélanome (carcinome épidermoïde)	B.1.13.1
4	Cancer de la peau non mélanome (carcinome à cellules basales)	B.1.13.2
3	Exécutions et conflit de la police	C.3.4
4	Tuberculose sensible aux médicaments	A.2.1.2
3	Virus zika	A.4.18
3	Ver de Guinée	A.4.19
4	Cardiomyopathie alcoolique	B.2.6.2
4	Violence sexuelle	C.3.2.3
4	Myocardite	B.2.6.1
4	Autres leucémies	B.1.28.5
4	Autre cardiomyopathie	B.2.6.3
3	Conflits et terrorisme	C.3.3
4	Tuberculose multirésistante sans grande résistance aux médicaments	A.2.1.3
4	Tuberculose ultra-résistante	A.2.1.4
4	VIH/SIDA - tuberculose sensible aux médicaments	A.1.1.1
4	VIH/SIDA - tuberculose multirésistante sans grande résistance aux médicaments	A.1.1.2
4	VIH/SIDA - tuberculose ultra-résistante	A.1.1.3
4	Infection tuberculeuse latente	A.2.1.1
2	VIH/SIDA et infections sexuellement transmissibles	A.1
2	Infections respiratoires et tuberculose	A.2
2	Infections entériques	A.3
3	Typhoïde et paratyphoïde	A.3.2
3	Salmonelle non typhoïdique invasive	A.3.3
2	Autres maladies infectieuses	A.5
2	Troubles maternels et néonatals	A.6
4	Syndromes myélodysplasiques, syndromes myéloprolifératifs, et autres tumeurs hématopoïétiques	B.1.30.1
4	Tumeurs intestinales bénignes et in situ	B.1.30.2
4	Tumeurs de col de l’utérus et du l’utérus, bénins et in situ	B.1.30.3
4	Autres tumeurs bénignes et in situs	B.1.30.4

4	Maladie de la valve aortique calcique non rhumatismale	B.2.5.1
4	Maladie de la valve mitrale dégénérative non rhumatismale	B.2.5.2
4	Autres valvulopathies non-rhumatismales	B.2.5.3
4	Cirrhose due à la téatohépatite non alcoolique	B.4.1.4
3	Céphalées	B.5.6
2	Troubles liés à l'utilisation de substances	B.7
2	Diabète et maladies rénales	B.8
4	Diabète sucré de type 1	B.8.1.1
4	Diabète sucré de type 2	B.8.1.2
4	Dermatite atopique	B.9.1.1
4	Dermatite de contact	B.9.1.2
4	Dermatite séborrhéique	B.9.1.3
3	Maladies bactériennes de la peau	B.9.3
3	Cécité et atteinte de la vision	B.10.1
3	Maladies du système digestif supérieur	B.4.2
4	Fausse couche et avortement maternelle	A.6.1.5
4	Cancer du foie dû à la téatohépatite non alcoolique	B.1.7.4
4	Maladie rénale chronique dû au diabète sucré de type 1	B.8.2.1
4	Maladie rénale chronique dûe au diabète sucré de type 2	B.8.2.2
4	Troubles de la réfraction	B.10.1.4
4	Presbytie	B.10.1.5

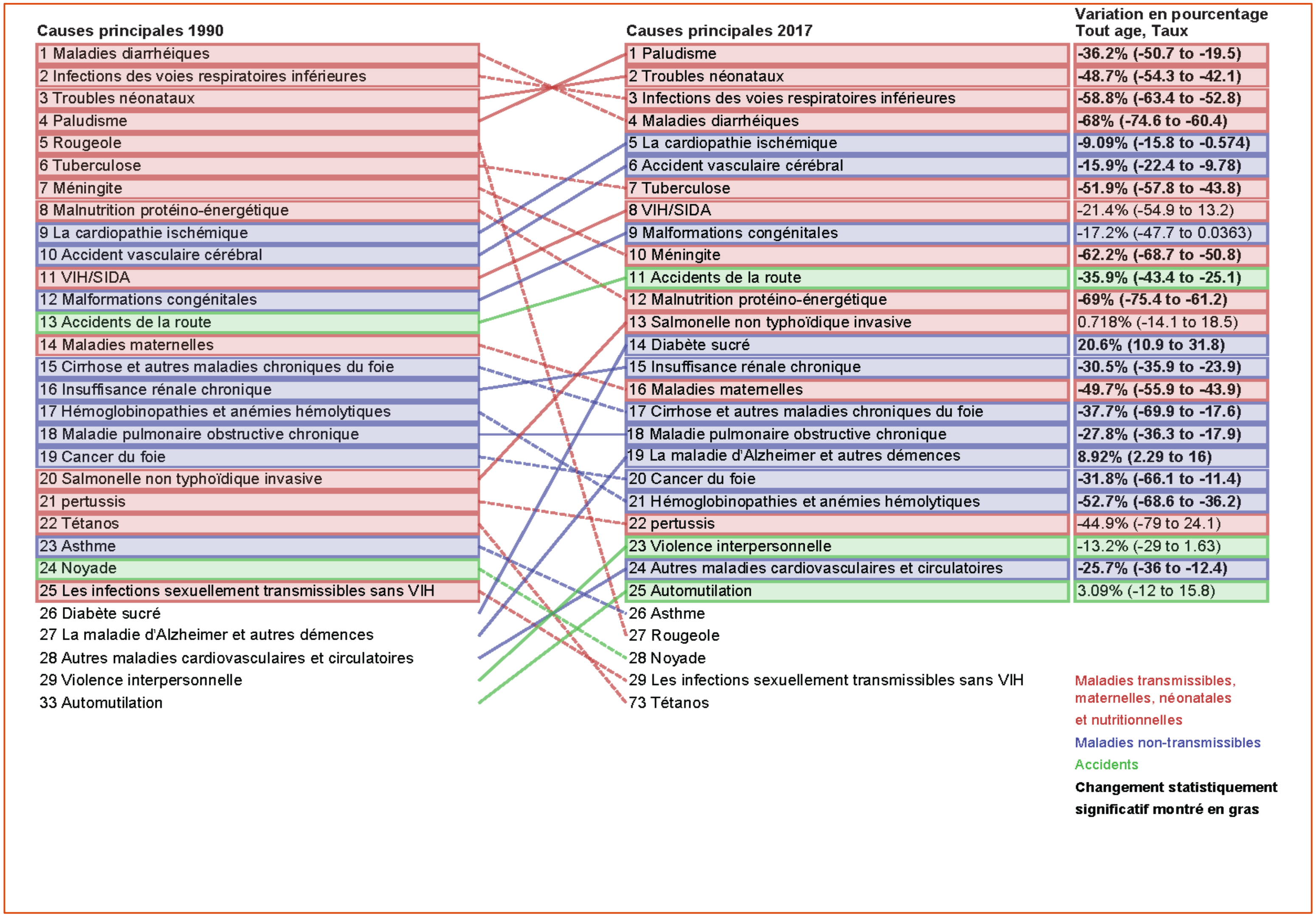

Figure 1: Principales causes de décès et leur évolution, tous âges, Communauté Economique des États de l'Afrique de l'Ouest, pays francophones, 1990-2017

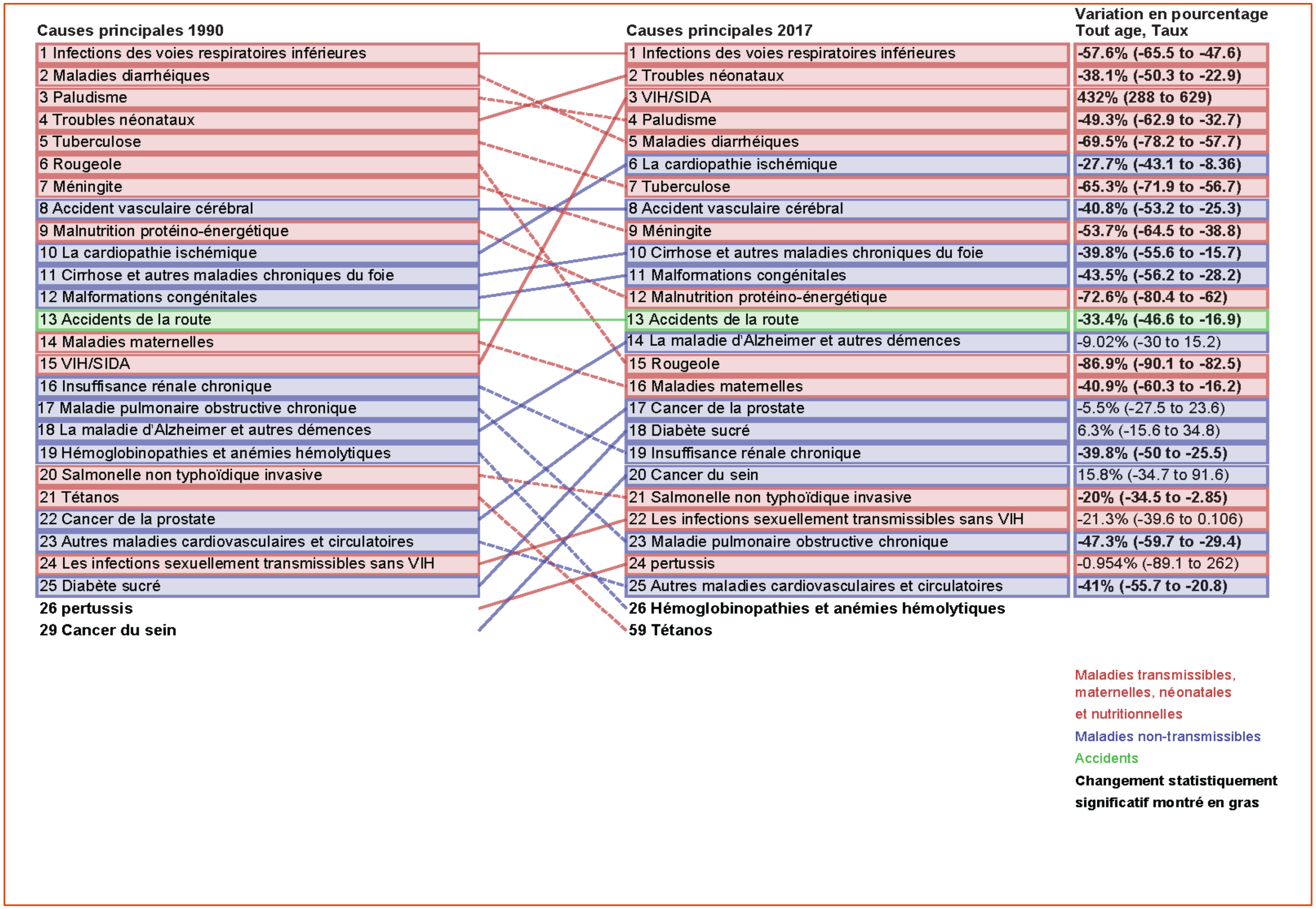

Figure 2: Principales causes de décès et leur évolution, tous âges, Communauté Economique des États de l'Afrique de l'Ouest, pays non-francophones, 1990-2017

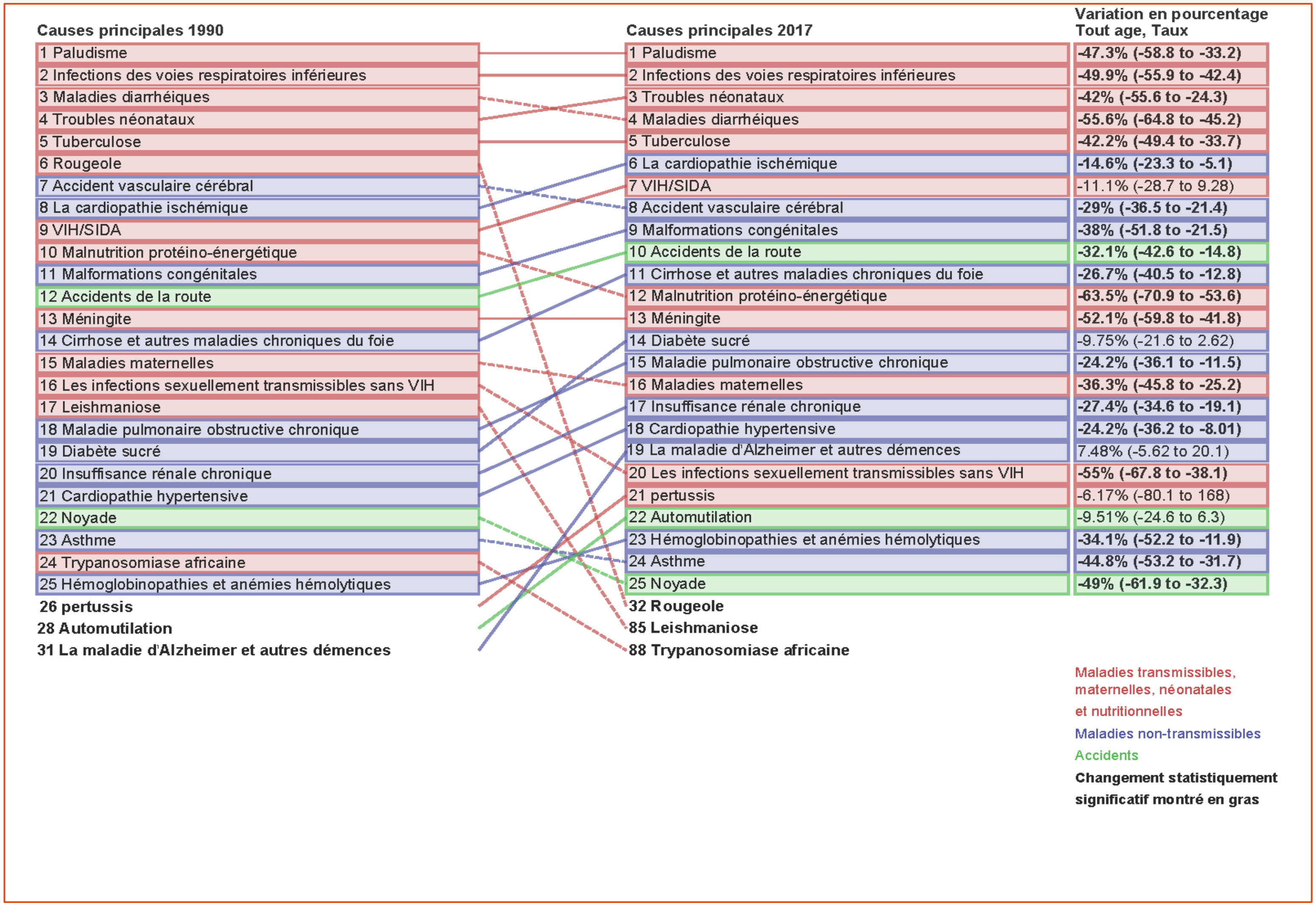

Figure 3**:** Principales causes de décès et leur évolution, tous âges, Communauté Economique des États d'Afrique Centrale, pays francophones, 1990-2017

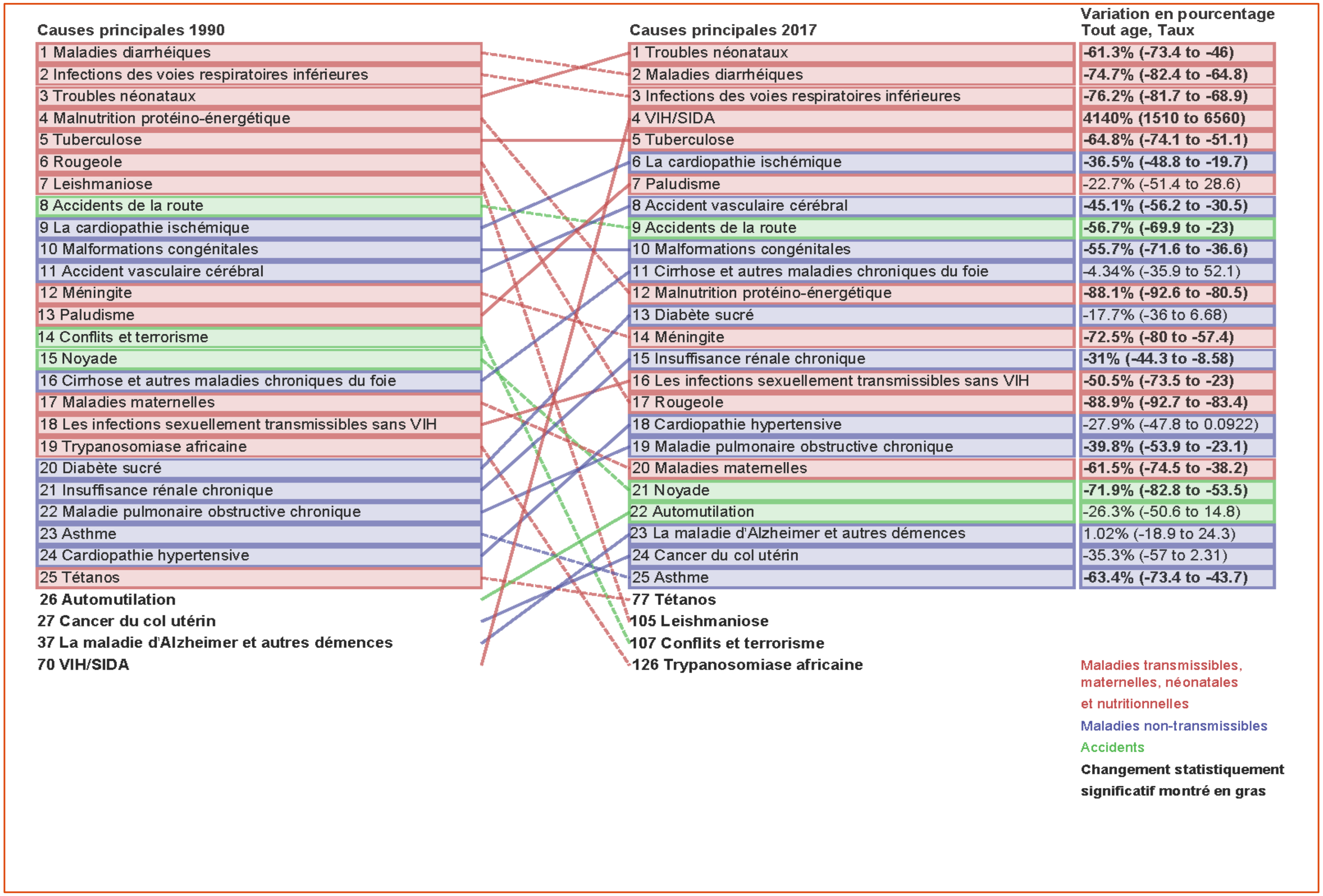

Figure 4: Principales causes de décès et leur évolution, tous âges, Communauté Economique des États d'Afrique Centrale, pays non-francophones, 1990-2017

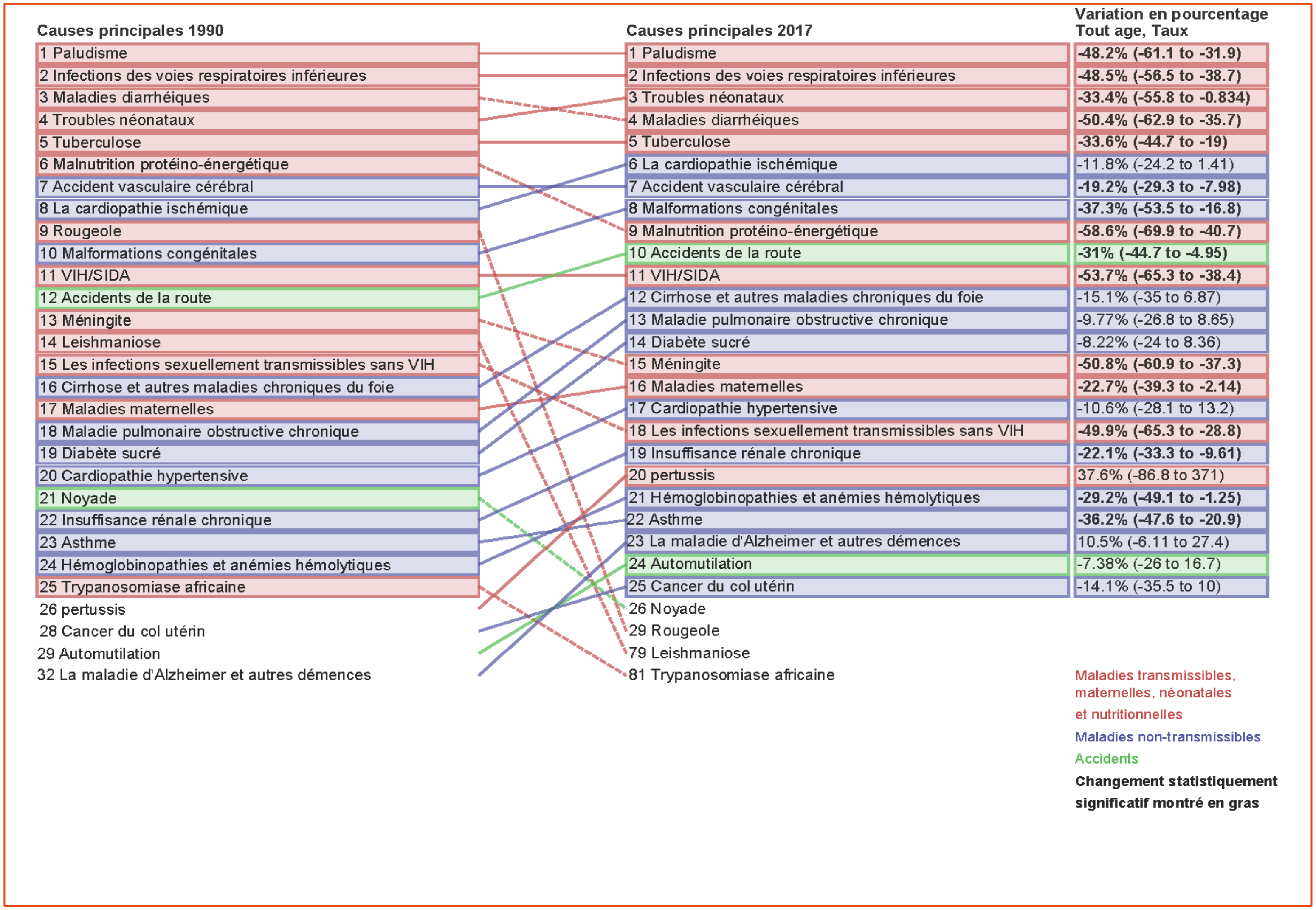

Figure 5: Principales causes de décès et leur évolution, tous âges, Communauté de Développement de l'Afrique Australe, pays francophones, 1990-2017

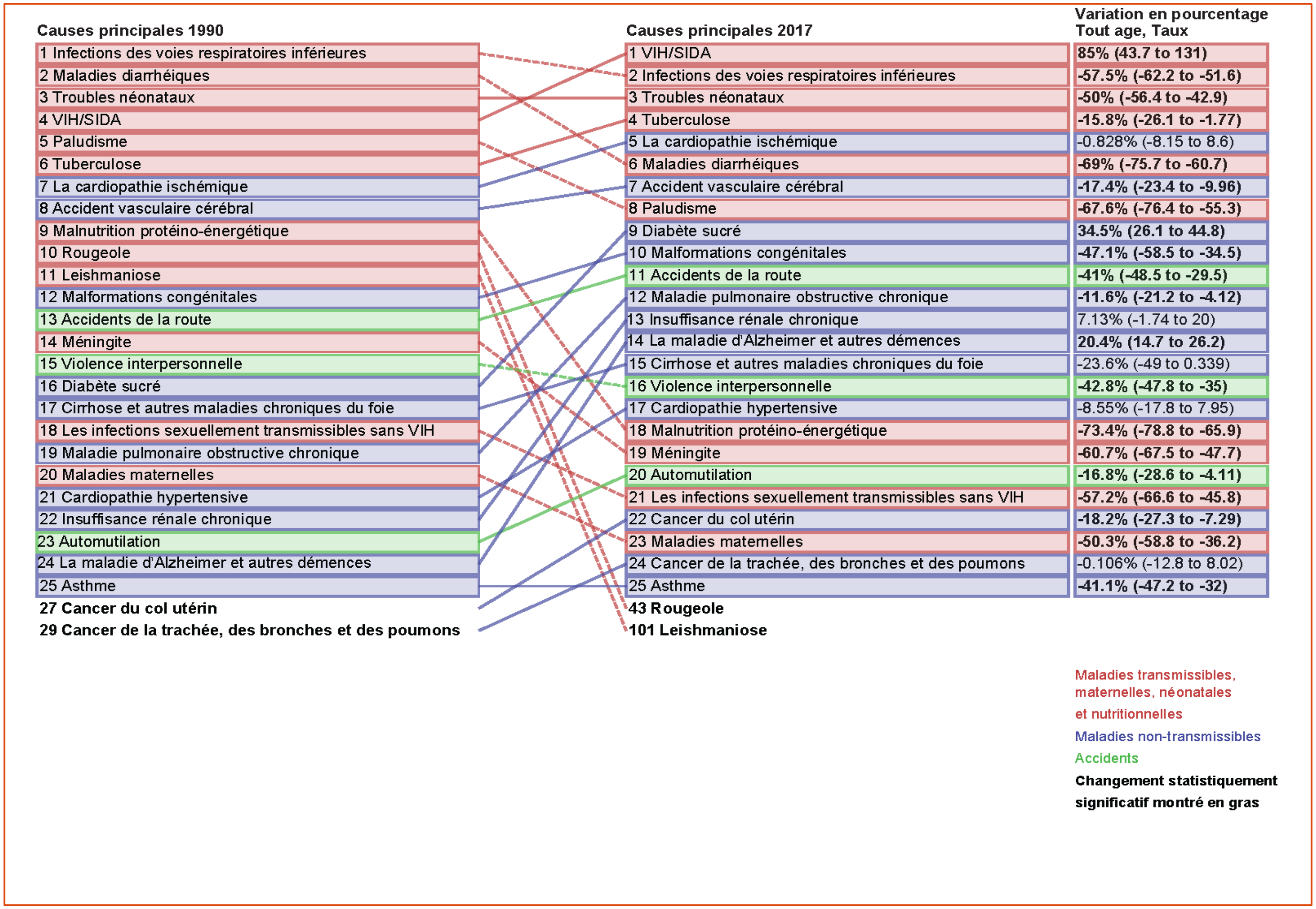

Figure 6**:** Principales causes de décès et leur évolution, tous âges, Communauté de Développement de l'Afrique Australe, pays non-francophones, 1990-2017

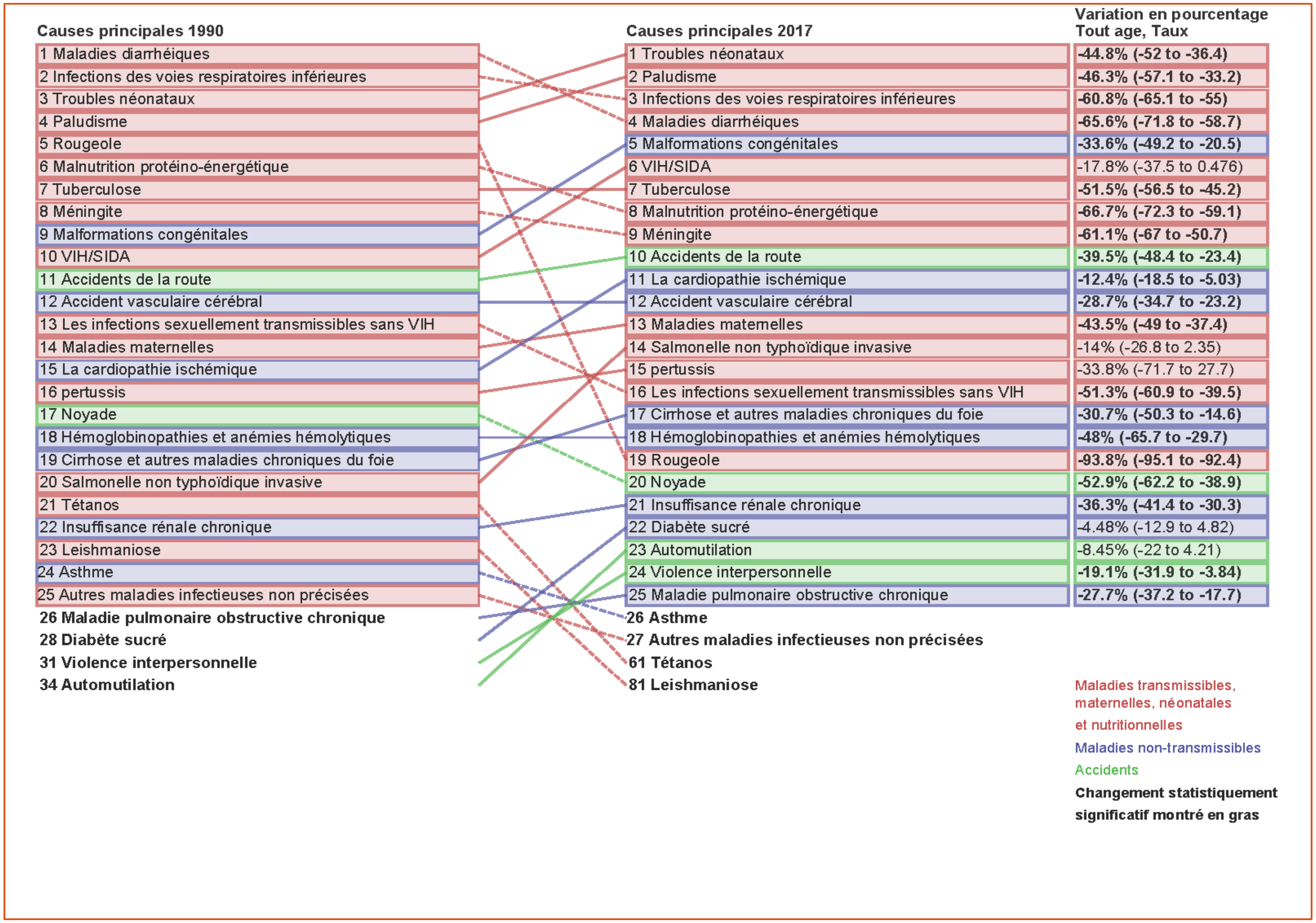

Figure 7**:** Principales causes des années de vie perdues et leur évolution, tous âges, Afrique francophone, 1990-2017

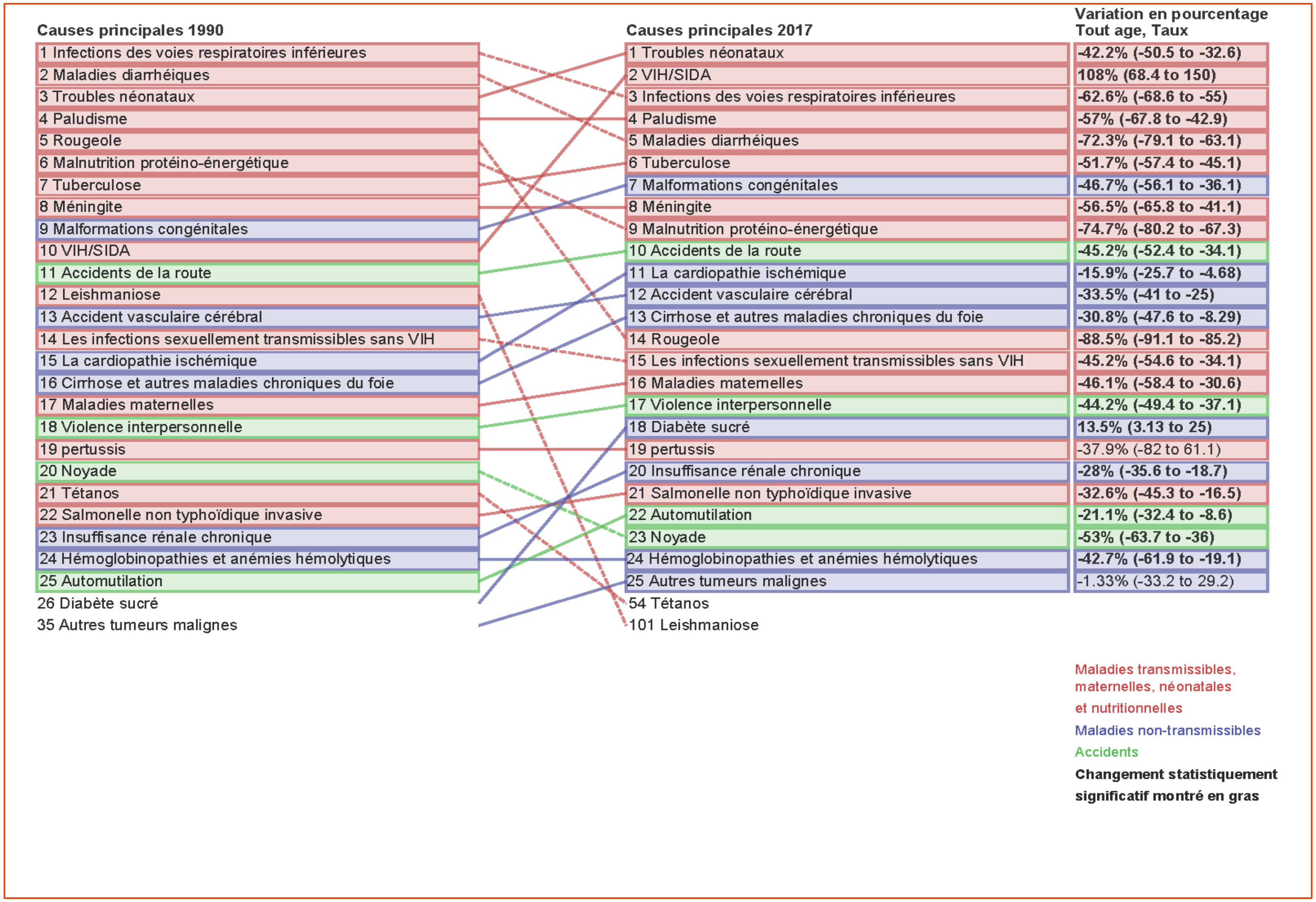

Figure 8: Principales causes des années de vie perdues et leur évolution, tous âges, Afrique non-francophone, 1990-2017

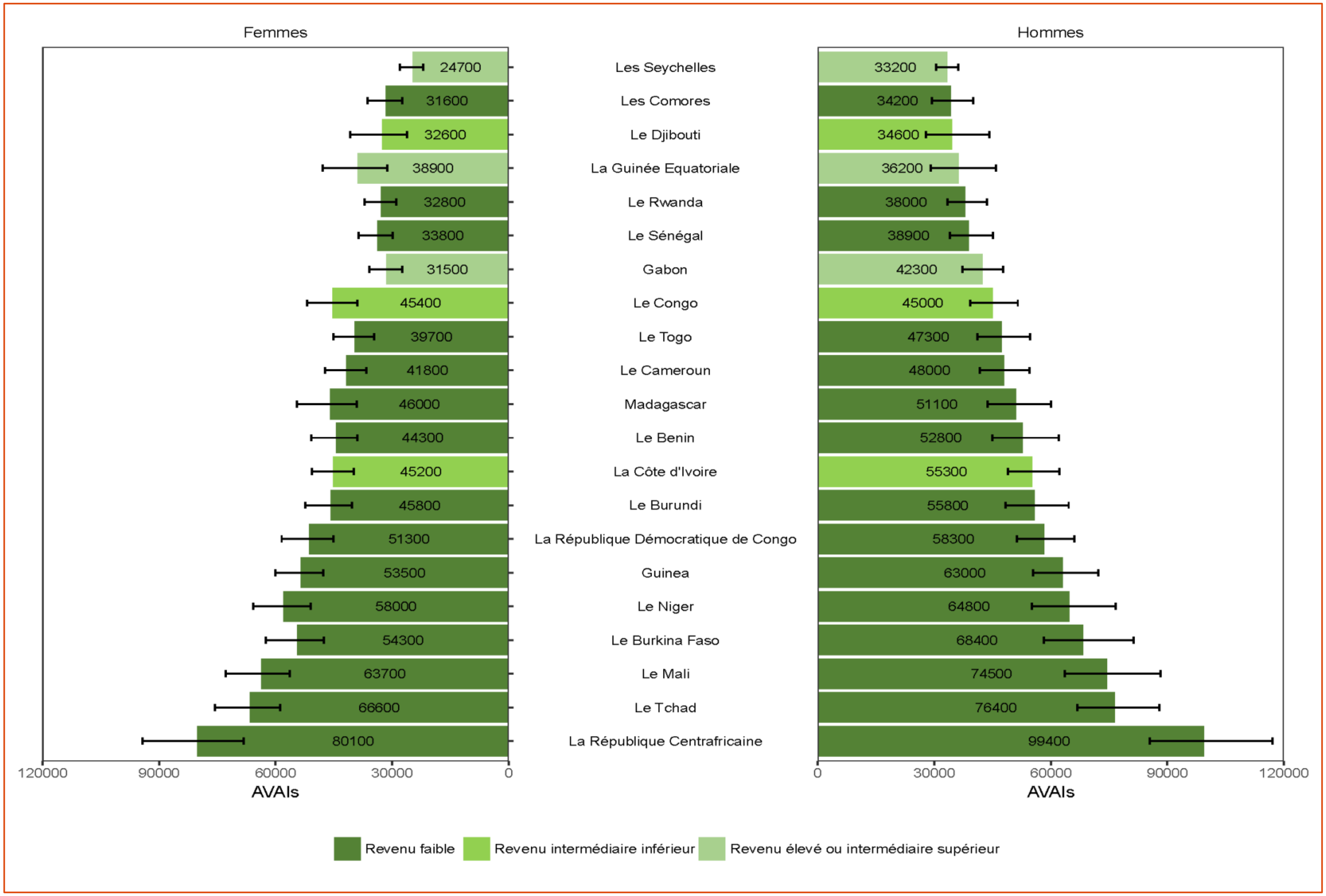

Figure 9**:** Années de vie ajustées de l'incapacité (AVAIs) pour 100 000 habitants, et intervalles d'incertitude (segments noirs), tous âges, par sexe et par pays, Afrique francophone, 2017

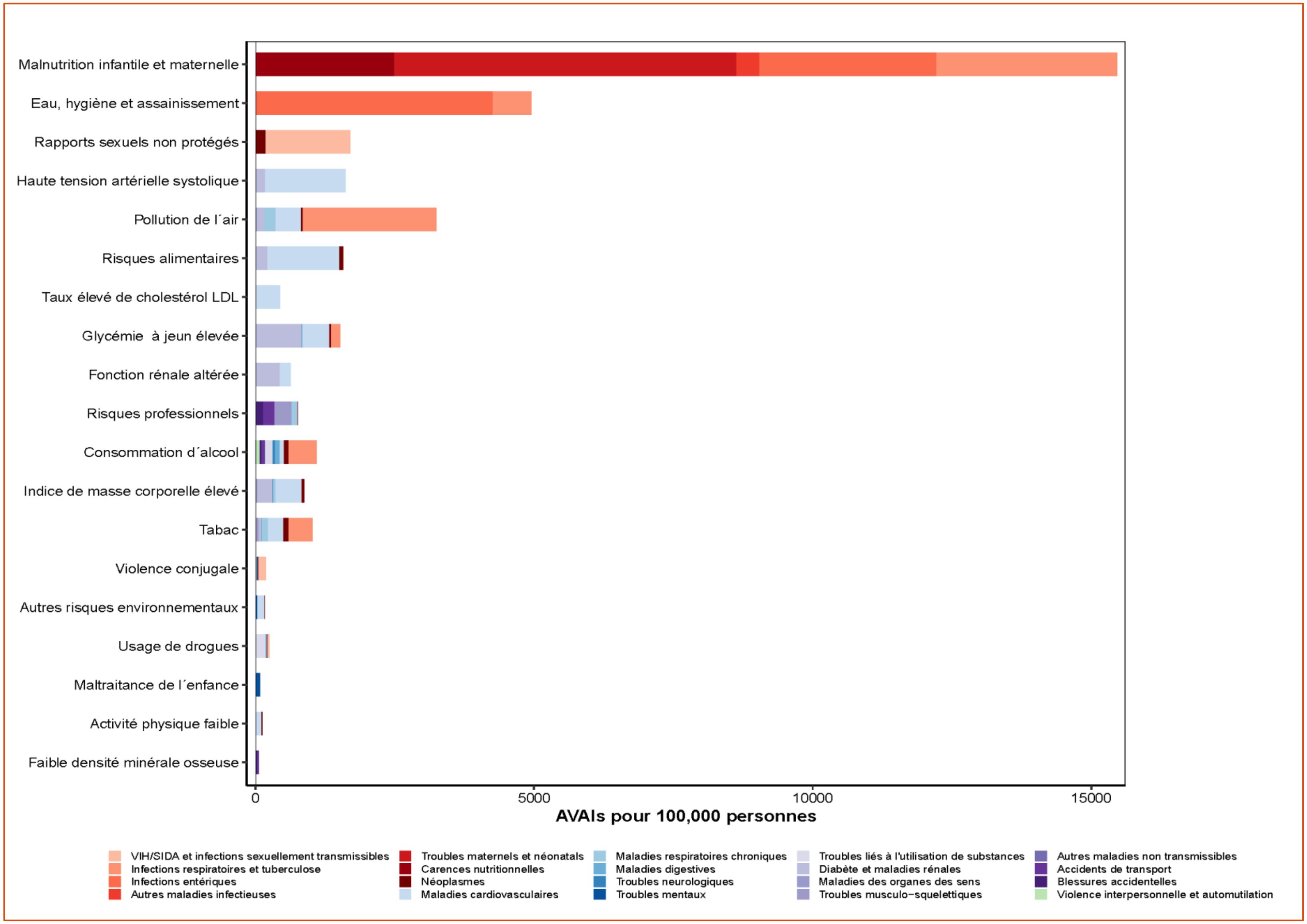

Figure 10: Facteurs de risque par années de vie ajustées de l'incapacité, Afrique francophone, 2017

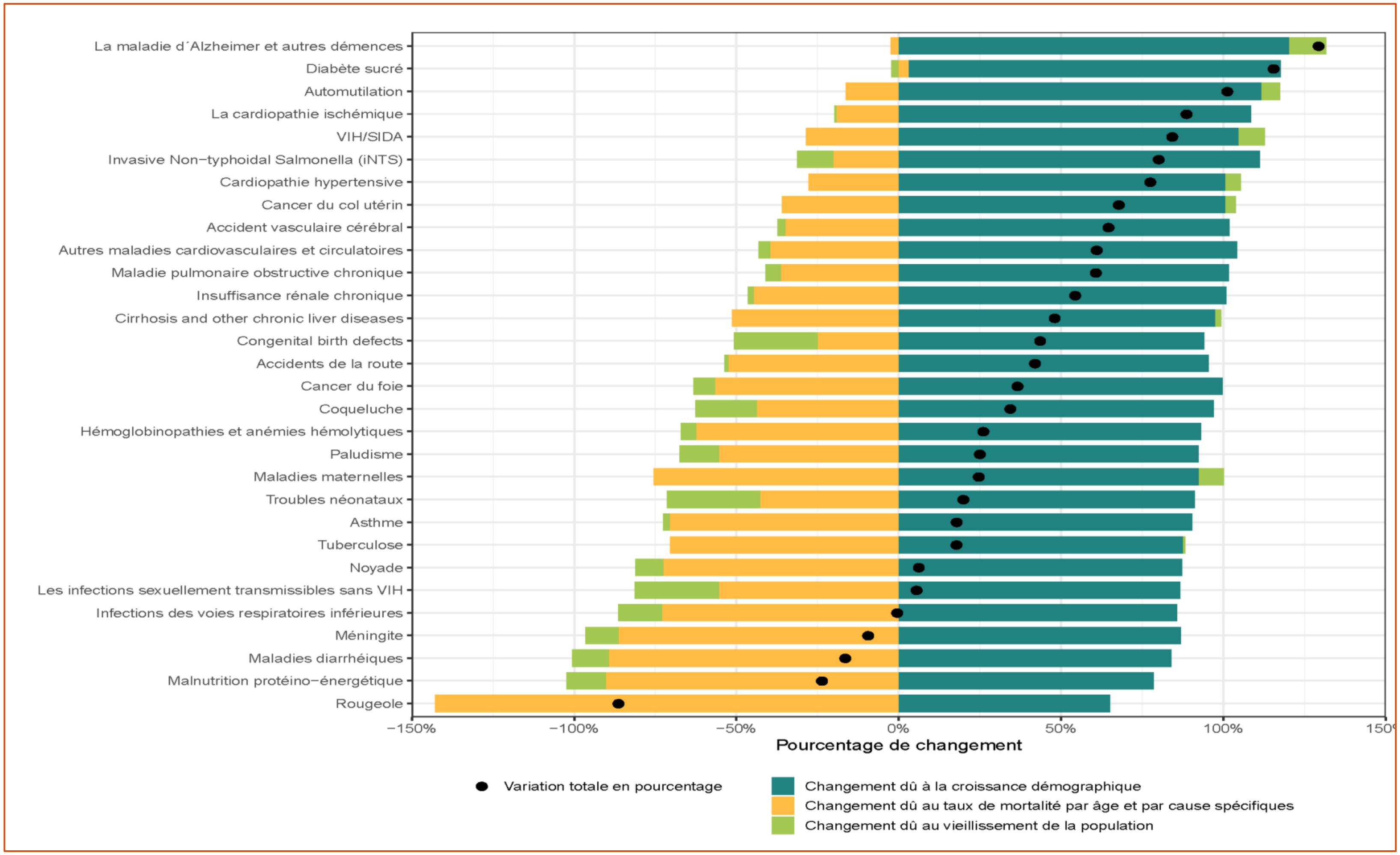

Figure 11**:** Décomposition du changement parmi les 30 principales causes de décès dues à la croissance démographique, au vieillissement de la population et au taux de mortalité par âge et par cause, Afrique francophone, 1990-2017. Par exemple, dans le cas de la maladie d'Alzheimer et d'autres démences, la mortalité par âge et par cause a contribué à une légère diminution des AVAIs, mais la croissance démographique et le vieillissement de la population ont entraîné une forte augmentation, ce qui fait que le pourcentage total de changement est d'environ 130 %.

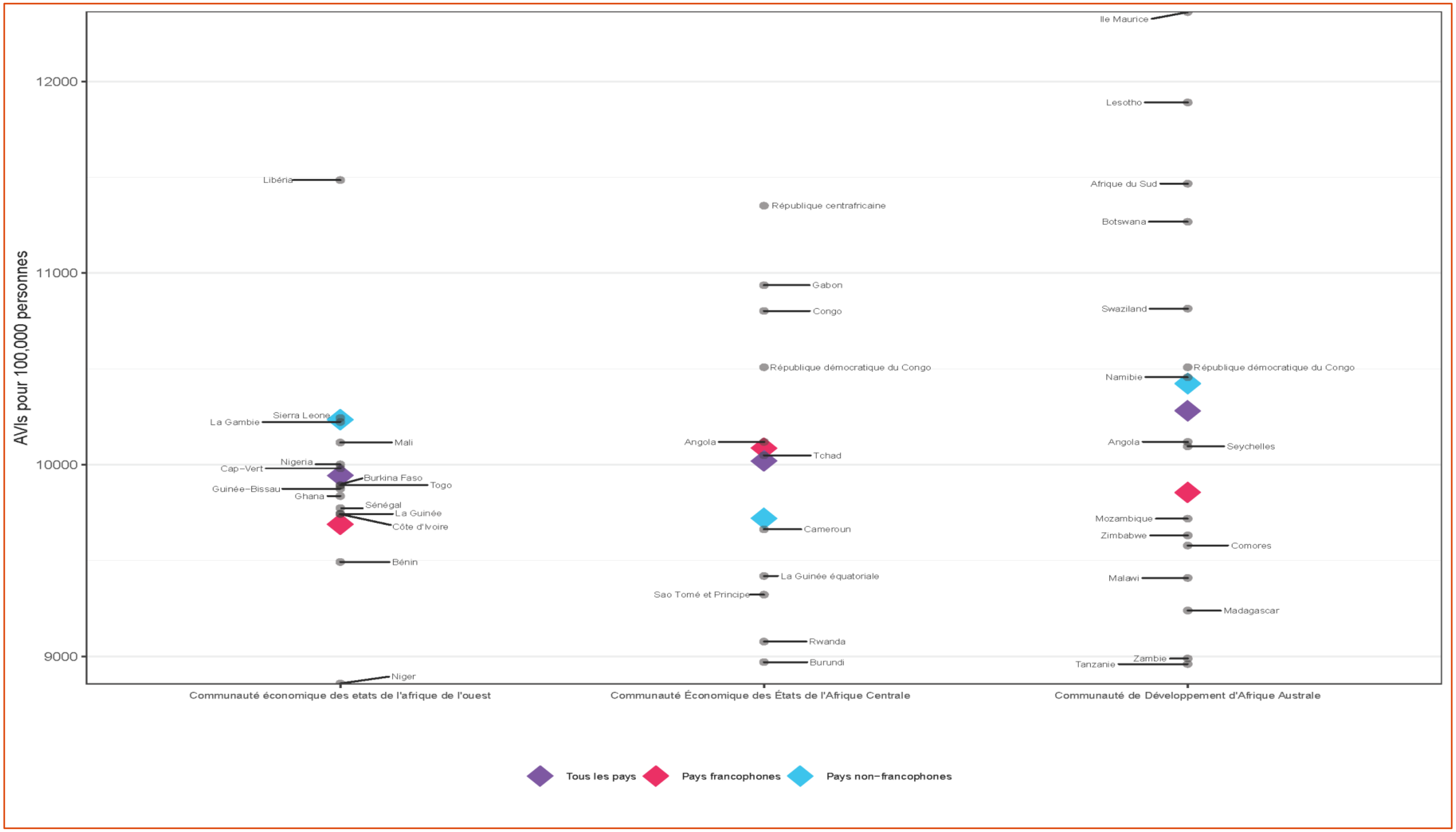

Figure 12**:** Années vécues avec une incapacité dans les pays francophones (étiquetés à droite) et non-francophones (étiquetés à gauche), au sein des trois communautés économiques

Tableau 3**:** Décès, années de vie perdues (AVPs), années vécues avec une incapacité (AVIs) et années de vie ajustées de l'incapacité (AVAIs) en 1990 et 2017, et espérance de vie, AVAIs prévues, AVIs prévues, indice sociodémographique (ISD) en 2017 et évolution en pourcentage de l'ISD entre 1990 et 2017

**Pays**	**1990**				**2017**								
	**Décès (pour 100,000)**	**AVPs (per 100,000)**	**AVIs (pour 100,000)**	**AVAIs (pour 100,000)**	**Décès (pour 100,000)**	**AVPs (per 100,000)**	**AVIs (pour 100,000)**	**AVAIs (pour 100,000)**	**Esperance de vie**	**AVAIs prévues (pour 100,000)**	**AVIs prévues (pour 100,000)**	**ISD**	**Evolution en pourcentage de l'ISD 1990–2010**
Le Bénin (BEN)	1320 (1250 à 1390)	85400 (80400 à 91000)	11300 (8270 à 14800)	96600 (90400 à 103000)	697 (615 à 793)	39000 (33400 à 46300)	9490 (7040 à 12500)	48500 (42200 à 56200)	64.6 (62.7 à 66.5)	58257.08	9762.98	0.37	40.5%
Le Burkina Faso (BFA)	1590 (1510 à 1680)	104000 (98600 à 111000)	12000 (8800 à 15800)	117000 (110000 à 124000)	866 (770 à 986)	51300 (43500 à 61100)	9890 (7320 à 13000)	61100 (53000 à 71700)	61.7 (59.9 à 63.2)	84844.96	10197.89	0.28	53.6%
Le Burundi (BDI)	1580 (1480 à 1690)	97500 (91000 à 104000)	9190 (6780 à 11900)	107000 (99400 à 114000)	751 (670 à 843)	41800 (36300 à 48400)	8970 (6710 à 11600)	50800 (44600 à 58000)	61.6 (59.7 à 63.4)	76297.43	10050.75	0.31	38.7%
Le Cameroun (CMR)	1150 (1090 à 1210)	71200 (66900 à 75900)	11000 (8190 à 14400)	82100 (77100 à 87600)	689 (612 à 767)	35200 (30700 à 39800)	9660 (7210 à 12600)	44800 (39700 à 50600)	63 (61.1 à 64.9)	40458.35	9613.75	0.48	29.2%
République Centrafricaine (CAF)	1640 (1520 à 1760)	98100 (90500 à 107000)	11900 (8830 à 15500)	110000 (101000 à 120000)	1380 (1210 à 1570)	78300 (67200 à 92900)	11400 (8490 à 14700)	89700 (77900 à 105000)	51.9 (49.8 à 54.1)	68562.77	9925.1	0.33	24.2%
Le Tchad (TCD)	1670 (1570 à 1770)	112000 (105000 à 119000)	11200 (8240 à 14600)	123000 (115000 à 131000)	963 (860 à 1080)	61400 (53700 à 71000)	10000 (7330 à 13100)	71500 (62900 à 81400)	60 (58.2 à 61.8)	95945.32	10401.52	0.25	28.0%
Les Comores (COM)	1050 (966 à 1150)	62200 (56000 à 68100)	9800 (7170 à 12900)	72000 (65500 à 78600)	615 (549 à 683)	23300 (20200 à 27300)	9580 (7120 à 12400)	32900 (28900 à 37700)	68.6 (67.1 à 70.2)	46806.31	9620.36	0.43	37.2%
Le Congo (COG)	1240 (1140 à 1340)	65300 (60000 à 70800)	10900 (8170 à 14300)	76200 (70400 à 82400)	772 (686 à 872)	34400 (30100 à 39500)	10800 (8030 à 14000)	45200 (39800 à 51100)	62.7 (60.8 à 64.6)	32604.6	10003.77	0.57	33.3%
La Cote d'Ivoire (CIV)	1150 (1060 à 1280)	75500 (69600 à 83000)	10600 (7750 à 13800)	86100 (79100 à 94800)	741 (663 à 819)	40600 (35900 à 46000)	9740 (7220 à 12800)	50400 (44900 à 56400)	62.5 (60.6 à 64.3)	51105.01	9663.38	0.41	31.7%
République Démocratique du Congo	1330 (1240 à	85600 (79700 à	11600 (8440 à	97200 (90300 à	795 (714 à	44300 (38500 à	10500 (7810 à	54800 (48500 à	62.4 (60.7 à	60612.28	9799.76	0.36	22.2%

(COD)	1410)	91500)	15100)	104000)	884)	50700)	13700)	62000)	64.1)				
Le Djibouti (DJI)	770 (657 à 896)	49600 (42600 à 57000)	8350 (6130 à 10900)	57900 (50700 à 65700)	559 (431 à 734)	24700 (18600 à 33300)	8940 (6650 à 11500)	33600 (27100 à 42700)	67.4 (64.1 à 70.2)	39860.25	9619.01	0.48	58.3%
La Guinée Equtoriale (GNQ)	1770 (1550 à 2010)	109000 (95200 à 125000)	11700 (8560 à 15500)	121000 (107000 à 137000)	540 (413 à 702)	28000 (21300 à 36900)	9420 (7070 à 12200)	37400 (30400 à 46700)	65.5 (62 à 68.8)	30856.14	10418.34	0.63	54.0%
Le eGabon (GAB)	1030 (961 à 1100)	47800 (44200 à 51500)	11300 (8280 à 14600)	59100 (54000 à 64000)	654 (593 à 716)	25800 (22800 à 28900)	10900 (8210 à 14100)	36700 (32500 à 41200)	68.4 (66.9 à 69.7)	30193.24	10627.78	0.65	29.2%
La Guinée (GIN)	1800 (1700 à 1890)	119000 (110000 à 126000)	10700 (7870 à 13900)	129000 (121000 à 137000)	918 (834 à 1020)	48400 (42300 à 55900)	9740 (7220 à 12600)	58100 (51600 à 65700)	60.7 (59.1 à 62.3)	71532.98	9972.47	0.32	34.4%
Le Madagascar (MDG)	1250 (1190 à 1310)	77800 (73400 à 82700)	10200 (7470 à 13300)	87900 (82300 à 93800)	708 (617 à 816)	39300 (33100 à 47500)	9240 (6950 à 12100)	48500 (41600 à 57000)	63.5 (61.6 à 65.4)	70026.43	9948.33	0.33	24.2%
Le Mali (MLI)	1920 (1830 à 2010)	133000 (126000 à 141000)	11400 (8440 à 15200)	144000 (137000 à 153000)	920 (813 à 1050)	59000 (50500 à 69800)	10100 (7450 à 13300)	69100 (59900 à 80300)	62 (60.2 à 63.5)	92215.71	103059.81	0.27	48.1%
Le Niger (NER)	2080 (1960 à 2200)	156000 (146000 à 167000)	9990 (7340 à 13200)	166000 (156000 à 177000)	809 (709 à 929)	52500 (44900 à 62200)	8860 (6600 à 11600)	61400 (53200 à 71100)	62.4 (60.5 à 64.1)	123593.81	10934.33	0.19	26.3%
Le Rwanda (RWA)	1550 (1480 à 1620)	93600 (89100 à 98200)	9090 (6740 à 11800)	103000 (97500 à 108000)	543 (493 à 598)	26200 (23200 à 30200)	9080 (6770 à 11700)	35300 (31300 à 40000)	68.5 (67.1 à 69.9)	52028.61	9674.84	0.41	56.1%
Le Sénégal (SEN)	1140 (1070 à 1200)	71000 (66100 à 75800)	10800 (7840 à 14200)	81700 (76200 à 87900)	596 (542 à 658)	26600 (23200 à 30900)	9770 (7280 à 12600)	36300 (32000 à 41700)	68 (66.7 à 69.3)	58257.08	9762.98	0.37	35.1%
Les Seychelles (SYC)	740 (724 à 758)	20600 (20000 à 21300)	8650 (6460 à 11100)	29300 (27000 à 31900)	756 (727 à 785)	19100 (18200 à 19900)	10100 (7560 à 13100)	29200 (26500 à 32100)	73.6 (73.1 à 74.1)	29076.79	10924.45	0.69	13.0%
Angola (TGO)	1060 (986 à 1140)	70000 (64600 à 75800)	10100 (7460 à 13200)	80100 (74200 à 86900)	671 (599 à 756)	33500 (29300 à 39100)	9890 (7320 à 12900)	43400 (38200 à 49300)	64.4 (62.6 à 66.2)	50212.42	9652.85	0.41	39.0%
Total	1423 (1395 à 1451)	92542 (90648 à 94501)	10887 (8034 à 14089)	103429 (100053 à 107349)	779 (750 à 809)	43708 (41673 à 45742)	9862 (7331 à 12749)	53570 (50164 à 57361)	64 (62.1 à 65.74)	67796.81	9963.50	0.35	31.4%

Tableau 4: Classement par étoiles des causes de décès pour les systèmes d'enregistrement de l'état civil, Afrique francophone

**Pays**	**Classement (0 – 5)**	**Etoiles**
République Centrafricaine	0	☆ ☆ ☆ ☆ ☆
Le Congo	0	☆ ☆ ☆ ☆ ☆
République Démocratique du Congo	1	★ ☆ ☆ ☆ ☆
La Guinée Equatoriale	0	☆ ☆ ☆ ☆ ☆
Le Gabon	0	☆ ☆ ☆ ☆ ☆
Le Burundi	1	★ ☆ ☆ ☆ ☆
Les Comores	0	☆ ☆ ☆ ☆ ☆
Le Djibouti	0	☆ ☆ ☆ ☆ ☆
Le Madagascar	1	★ ☆ ☆ ☆ ☆
Le Rwanda	1	★ ☆ ☆ ☆ ☆
Les Seychelles	3	★ ★ ★ ☆ ☆
Le Bénin	1	★ ☆ ☆ ☆ ☆
Burkina Faso	1	★ ☆ ☆ ☆ ☆
Le Cameroun	0	☆ ☆ ☆ ☆ ☆
Le Tchad	0	☆ ☆ ☆ ☆ ☆
La Cote d'Ivoire	1	★ ☆ ☆ ☆ ☆
Le Guinée	1	★ ☆ ☆ ☆ ☆
Le Mali	1	★ ☆ ☆ ☆ ☆
Le Niger	1	★ ☆ ☆ ☆ ☆
Le Sénégal	1	★ ☆ ☆ ☆ ☆
Angola	0	☆ ☆ ☆ ☆ ☆
