# Supplementary material for: Burden of disease in francophone Africa, 1990–2017: a systematic analysis for the Global Burden of Disease Study 2017
Source: Lancet Glob Health. 2020 Feb 19;8(3):e341–51. doi: 10.1016/S2214-109X(20)30024-3 (PMC7034333; doi:10.1016/S2214-109X(20)30024-3)
Supplement: Supplementary appendix 2 [file mmc2.pdf]

THE LANCET
Global Health

Supplementary appendix 2

This appendix formed part of the original submission and has been peer reviewed. We post it as supplied by the authors.

Supplement to: El Bcheraoui C, Mimche H, Miangotar Y, et al. Burden of disease in francophone Africa, 1990–2017: a systematic analysis for the Global Burden of Disease Study 2017. *Lancet Glob Health* 2020; **8:** e341–51.

Contents

Table 1: Distribution of francophone African countries (marked by an asterisk), along with their non-francophone counterparts, by economic groups, and sociodemographic index (SDI) 3

Table 2: Causes of death levels of cause hierarchy 4

Figure 1: Leading causes of death and their evolution, all ages, Economic Community of West African States, francophone countries, 1990–2017 14

Figure 2: Leading causes of death and their evolution, all ages, Economic Community of West African States, non-francophone countries, 1990–2017 15

Figure 3: Leading causes of death and their evolution, all ages, Economic Community of Central African States, francophone countries, 1990–2017. 16

Figure 4: Leading causes of death and their evolution, all ages, Economic Community of Central African States, non-francophone countries, 1990–2017 17

Figure 5: Leading causes of death and their evolution, all ages, Southern African Development Community, francophone countries, 1990–2017 18

Figure 6: Leading causes of death and their evolution, all ages, Southern African Development Community, non-francophone countries, 1990–2017 19

Figure 7: main causes of years of life lost and their evolution, all ages, francophone Africa, 1990–2017 20

Figure 8: main causes of years of life lost and their evolution, all ages, non-francophone Africa, 1990–2017 21

Figure 9: Disability-adjusted life-years (DALYs) per 100,000 population, and uncertainty intervals (black segments), all ages, by sex and country, francophone Africa, 2017 22

Figure 10: Risk factors by disability-adjusted life years, francophone Africa, 2017 23

Figure 11: Decomposition of change among the 30 main causes of death due to population growth, population ageing, and age- and cause-specific mortality rate, francophone Africa, 1990–2017. For example, in the case on Alzheimer's disease and other dementias, age- and cause-specific mortality have contributed to a small decrease in DALYs, but population growth and population ageing drove a large increase making the total percent change about 130%. 24

Figure 12: Years lived with disability (YLDs) in francophone (labeled to the right) and non-francophone (labelled to the left) countries, within the three economic communities 25

Table 3: Deaths, years of life lost (YLLs), years lived with disability (YLDs), and disability-adjusted life-years (DALYs) in 1990 and 2017, and life expectancy, expected DALYs, sociodemographic index (SDI) in 2017, and percent change in SDI between 1990 and 2017 26

Table 4 : Cause of death star rating for vital registration systems, francophone Africa....28

Table 1: Distribution of francophone African countries (marked by an asterisk), along with their non-francophone counterparts, by economic groups, and sociodemographic index (SDI)

**Economic Community of West African States (SDI)**	**Economic Community of Central African States (SDI)**	**Southern African Development Community (SDI)**
Benin* (0.37)	Burundi* (0.31)	Comoros* (0.43)
Burkina Faso* (0.28)	Central African Republic* (0.33)	Democratic Republic of the Congo* (0.36)
Cote d'Ivoire* (0.41)	Cameroon* (0.48)	Madagascar* (0.33)
Guinea* (0.32)	Chad* (0.25)	Seychelles* (0.69)
Guinea Bissau* (0.35)	Congo* (0.57)	Angola (0.46)
Mali* (0.27)	Democratic Republic of the Congo* (0.36)	Botswana (0.66)
Niger* (0.19)	Equatorial Guinea* (0.63)	Lesotho (0.49)
Senegal* (0.37)	Gabon* (0.65)	Malawi (0.35)
Togo* (0.41)	Rwanda* (0.41)	Mauritius (0.72)
Cape Verde (0.55)	Angola (0.46)	Mozambique (0.34)
Ghana (0.54)	Sao Tome and Principe (0.49)	Namibia (0.62)
Liberia (0.33)		South Africa (0.68)
Nigeria (0.49)		Swaziland (0.58)
Sierra Leone (0.36)		Tanzania (0.41)
The Gambia (0.40)		Zambia (0.47)
		Zimbabwe (0.46)

Table 2**:** Causes of death levels of cause hierarchy

**Level**	**Cause name**	**Cause outline**
0	All causes	Total
1	Communicable, maternal, neonatal, and nutritional disorders	A
2	HIV/AIDS and sexually transmitted infections	A.1
3	HIV/AIDS	A.1.1
4	HIV/AIDS - Drug-susceptible Tuberculosis	A.1.1.1
4	HIV/AIDS - Multidrug-resistant Tuberculosis without extensive drug resistance	A.1.1.2
4	HIV/AIDS - Extensively drug-resistant Tuberculosis	A.1.1.3
4	HIV/AIDS resulting in other diseases	A.1.1.4
3	Sexually transmitted infections excluding HIV	A.1.2
4	Syphilis	A.1.2.1
4	Chlamydial infection	A.1.2.2
4	Gonococcal infection	A.1.2.3
4	Other sexually transmitted infections	A.1.2.6
2	Respiratory infections and tuberculosis	A.2
3	Tuberculosis	A.2.1
4	Drug-susceptible tuberculosis	A.2.1.2
4	Multidrug-resistant tuberculosis without extensive drug resistance	A.2.1.3
4	Extensively drug-resistant tuberculosis	A.2.1.4
3	Lower respiratory infections	A.2.2
3	Upper respiratory infections	A.2.3
3	Otitis media	A.2.4
2	Enteric infections	A.3
3	Diarrhoeal diseases	A.3.1
3	Typhoid and paratyphoid	A.3.2
4	Typhoid fever	A.3.2.1
4	Paratyphoid fever	A.3.2.2
3	iNTS	A.3.3
3	Other intestinal infectious diseases	A.3.5

2	Neglected tropical diseases and malaria	A.4
3	Malaria	A.4.1
3	Chagas disease	A.4.2
3	Leishmaniasis	A.4.3
4	Visceral leishmaniasis	A.4.3.1
3	African trypanosomiasis	A.4.4
3	Schistosomiasis	A.4.5
3	Cysticercosis	A.4.6
3	Cystic echinococcosis	A.4.7
3	Dengue	A.4.11
3	Yellow fever	A.4.12
3	Rabies	A.4.13
3	Intestinal nematode infections	A.4.14
4	Ascariasis	A.4.14.1
3	Ebola virus disease	A.4.17
3	Zika virus disease	A.4.18
3	Other neglected tropical diseases	A.4.20
2	Other infectious diseases	A.5
3	Meningitis	A.5.1
4	Pneumococcal meningitis	A.5.1.1
4	H influenzae type B meningitis	A.5.1.2
4	Meningococcal infection	A.5.1.3
4	Other meningitis	A.5.1.4
3	Encephalitis	A.5.2
3	Diphtheria	A.5.3
3	Whooping cough	A.5.4
3	Tetanus	A.5.5
3	Measles	A.5.6
3	Varicella and herpes zoster	A.5.7
3	Acute hepatitis	A.5.8
4	Acute hepatitis A	A.5.8.1

4	Acute hepatitis B	A.5.8.2
4	Acute hepatitis C	A.5.8.3
4	Acute hepatitis E	A.5.8.4
3	Other unspecified infectious diseases	A.5.9
2	Maternal and neonatal disorders	A.6
3	Maternal disorders	A.6.1
4	Maternal haemorrhage	A.6.1.1
4	Maternal sepsis and other pregnancy related infections	A.6.1.2
4	Maternal hypertensive disorders	A.6.1.3
4	Maternal obstructed labour and uterine rupture	A.6.1.4
4	Maternal abortive outcome	A.6.1.5
4	Ectopic pregnancy	A.6.1.6
4	Indirect maternal deaths	A.6.1.7
4	Late maternal deaths	A.6.1.8
4	Maternal deaths aggravated by HIV/AIDS	A.6.1.9
4	Other maternal disorders	A.6.1.10
3	Neonatal disorders	A.6.2
4	Neonatal preterm birth	A.6.2.1
4	Neonatal encephalopathy due to birth asphyxia and trauma	A.6.2.2
4	Neonatal sepsis and other neonatal infections	A.6.2.3
4	Hemolytic disease and other neonatal jaundice	A.6.2.4
4	Other neonatal disorders	A.6.2.5
2	Nutritional deficiencies	A.7
3	Protein-energy malnutrition	A.7.1
3	Other nutritional deficiencies	A.7.5
1	Non-communicable diseases	B
2	Neoplasms	B.1
3	Lip and oral cavity cancer	B.1.1
3	Nasopharynx cancer	B.1.2
3	Other pharynx cancer	B.1.3
3	Oesophageal cancer	B.1.4

3	Stomach cancer	B.1.5
3	Colon and rectum cancer	B.1.6
3	Liver cancer	B.1.7
4	Liver cancer due to hepatitis B	B.1.7.1
4	Liver cancer due to hepatitis C	B.1.7.2
4	Liver cancer due to alcohol use	B.1.7.3
4	Liver cancer due to NASH	B.1.7.4
4	Liver cancer due to other causes	B.1.7.5
3	Gallbladder and biliary tract cancer	B.1.8
3	Pancreatic cancer	B.1.9
3	Larynx cancer	B.1.10
3	Tracheal, bronchus, and lung cancer	B.1.11
3	Malignant skin melanoma	B.1.12
3	Non-melanoma skin cancer	B.1.13
4	Non-melanoma skin cancer (squamous-cell carcinoma)	B.1.13.1
3	Breast cancer	B.1.14
3	Cervical cancer	B.1.15
3	Uterine cancer	B.1.16
3	Ovarian cancer	B.1.17
3	Prostate cancer	B.1.18
3	Testicular cancer	B.1.19
3	Kidney cancer	B.1.20
3	Bladder cancer	B.1.21
3	Brain and nervous system cancer	B.1.22
3	Thyroid cancer	B.1.23
3	Mesothelioma	B.1.24
3	Hodgkin lymphoma	B.1.25
3	Non-Hodgkin's lymphoma	B.1.26
3	Multiple myeloma	B.1.27
3	Leukaemia	B.1.28
4	Acute lymphoid leukaemia	B.1.28.1

4	Chronic lymphoid leukaemia	B.1.28.2
4	Acute myeloid leukaemia	B.1.28.3
4	Chronic myeloid leukaemia	B.1.28.4
4	Other leukaemia	B.1.28.5
3	Other malignant cancers	B.1.29
3	Other neoplasms	B.1.30
4	Myelodysplastic, myeloproliferative, and other hematopoietic neoplasms	B.1.30.1
4	Other benign and in situ neoplasms	B.1.30.4
2	Cardiovascular diseases	B.2
3	Rheumatic heart disease	B.2.1
3	Ischaemic heart disease	B.2.2
3	Stroke	B.2.3
4	Ischaemic stroke	B.2.3.1
4	Intracerebral hemorrhage	B.2.3.2
4	Subarachnoid hemorrhage	B.2.3.3
3	Hypertensive heart disease	B.2.4
3	Non-rheumatic valvular heart disease	B.2.5
4	Non-rheumatic calcific aortic valve disease	B.2.5.1
4	Non-rheumatic degenerative mitral valve disease	B.2.5.2
4	Other non-rheumatic valve diseases	B.2.5.3
3	Cardiomyopathy and myocarditis	B.2.6
4	Myocarditis	B.2.6.1
4	Alcoholic cardiomyopathy	B.2.6.2
4	Other cardiomyopathy	B.2.6.3
3	Atrial fibrillation and flutter	B.2.7
3	Aortic aneurysm	B.2.8
3	Peripheral vascular disease	B.2.9
3	Endocarditis	B.2.10
3	Other cardiovascular and circulatory diseases	B.2.11
2	Chronic respiratory diseases	B.3
3	Chronic obstructive pulmonary disease	B.3.1

3	Pneumoconiosis	B.3.2
4	Silicosis	B.3.2.1
4	Asbestosis	B.3.2.2
4	Coal workers pneumoconiosis	B.3.2.3
4	Other pneumoconiosis	B.3.2.4
3	Asthma	B.3.3
3	Interstitial lung disease and pulmonary sarcoidosis	B.3.4
3	Other chronic respiratory diseases	B.3.5
2	Digestive diseases	B.4
3	Cirrhosis and other chronic liver diseases	B.4.1
4	Cirrhosis and other chronic liver diseases due to hepatitis B	B.4.1.1
4	Cirrhosis and other chronic liver diseases due to hepatitis C	B.4.1.2
4	Cirrhosis and other chronic liver diseases due to alcohol use	B.4.1.3
4	Cirrhosis due to NASH	B.4.1.4
4	Cirrhosis and other chronic liver diseases due to other causes	B.4.1.5
3	Upper digestive system diseases	B.4.2
4	Peptic ulcer disease	B.4.2.1
4	Gastritis and duodenitis	B.4.2.2
3	Appendicitis	B.4.3
3	Paralytic ileus and intestinal obstruction	B.4.4
3	Inguinal, femoral, and abdominal hernia	B.4.5
3	Inflammatory bowel disease	B.4.6
3	Vascular intestinal disorders	B.4.7
3	Gallbladder and biliary diseases	B.4.8
3	Pancreatitis	B.4.9
3	Other digestive diseases	B.4.10
2	Neurological disorders	B.5
3	Alzheimer's disease and other dementias	B.5.1
3	Parkinson's disease	B.5.2
3	Epilepsy	B.5.3
3	Multiple sclerosis	B.5.4
3	Motor neuron disease	B.5.5

3	Other neurological disorders	B.5.7
2	Mental disorders	B.6
3	Eating disorders	B.6.5
4	Anorexia nervosa	B.6.5.1
4	Bulimia nervosa	B.6.5.2
2	Substance use disorders	B.7
3	Alcohol use disorders	B.7.1
3	Drug use disorders	B.7.2
4	Opioid use disorders	B.7.2.1
4	Cocaine use disorders	B.7.2.2
4	Amphetamine use disorders	B.7.2.3
4	Other drug use disorders	B.7.2.5
2	Diabetes and kidney diseases	B.8
3	Diabetes mellitus	B.8.1
4	Diabetes mellitus type 1	B.8.1.1
4	Diabetes mellitus type 2	B.8.1.2
3	Chronic kidney disease	B.8.2
4	Chronic kidney disease due to diabetes mellitus type 1	B.8.2.1
4	Chronic kidney disease due to diabetes mellitus type 2	B.8.2.2
4	Chronic kidney disease due to hypertension	B.8.2.3
4	Chronic kidney disease due to glomerulonephritis	B.8.2.4
4	Chronic kidney disease due to other and unspecified causes	B.8.2.5
3	Acute glomerulonephritis	B.8.3
2	Skin and subcutaneous diseases	B.9
3	Bacterial skin diseases	B.9.3
4	Cellulitis	B.9.3.1
4	Pyoderma	B.9.3.2
3	Decubitus ulcer	B.9.11
3	Other skin and subcutaneous diseases	B.9.12
2	Musculoskeletal disorders	B.11
3	Rheumatoid arthritis	B.11.1

3	Other musculoskeletal disorders	B.11.6
2	Other non-communicable diseases	B.12
3	Congenital anomalies	B.12.1
4	Neural tube defects	B.12.1.1
4	Congenital heart anomalies	B.12.1.2
4	Orofacial clefts	B.12.1.3
4	Down's syndrome	B.12.1.4
4	Other chromosomal abnormalities	B.12.1.7
4	Congenital musculoskeletal and limb anomalies	B.12.1.8
4	Urogenital congenital anomalies	B.12.1.9
4	Digestive congenital anomalies	B.12.1.10
4	Other congenital anomalies	B.12.1.11
3	Urinary diseases and male infertility	B.12.2
4	Urinary tract infections	B.12.2.1
4	Urolithiasis	B.12.2.2
4	Other urinary diseases	B.12.2.5
3	Gynecological diseases	B.12.3
4	Uterine fibroids	B.12.3.1
4	Polycystic ovarian syndrome	B.12.3.2
4	Endometriosis	B.12.3.4
4	Genital prolapse	B.12.3.5
4	Other gynecological diseases	B.12.3.7
3	Hemoglobinopathies and hemolytic anaemias	B.12.4
4	Thalassemias	B.12.4.1
4	Sickle cell disorders	B.12.4.3
4	G6PD deficiency	B.12.4.5
4	Other hemoglobinopathies and hemolytic anaemias	B.12.4.7
3	Endocrine, metabolic, blood, and immune disorders	B.12.5
3	Sudden infant death syndrome	B.12.7
1	Injuries	C
2	Transport injuries	C.1

3	Road injuries	C.1.1
4	Pedestrian road injuries	C.1.1.1
4	Cyclist road injuries	C.1.1.2
4	Motorcyclist road injuries	C.1.1.3
4	Motor vehicle road injuries	C.1.1.4
4	Other road injuries	C.1.1.5
3	Other transport injuries	C.1.2
2	Unintentional injuries	C.2
3	Falls	C.2.1
3	Drowning	C.2.2
3	Fire, heat, and hot substances	C.2.3
3	Poisonings	C.2.4
4	Poisoning by carbon monoxide	C.2.4.1
4	Poisoning by other means	C.2.4.2
3	Exposure to mechanical forces	C.2.5
4	Unintentional firearm injuries	C.2.5.1
4	Other exposure to mechanical forces	C.2.5.2
3	Adverse effects of medical treatment	C.2.6
3	Animal contact	C.2.7
4	Venomous animal contact	C.2.7.1
4	Non-venomous animal contact	C.2.7.2
3	Foreign body	C.2.8
4	Pulmonary aspiration and foreign body in airway	C.2.8.1
4	Foreign body in other body part	C.2.8.3
3	Environmental heat and cold exposure	C.2.9
3	Exposure to forces of nature	C.2.10
3	Other unintentional injuries	C.2.11
2	Self-harm and interpersonal violence	C.3
3	Self-harm	C.3.1
4	Self-harm by firearm	C.3.1.1
4	Self-harm by other specified means	C.3.1.2

3	Interpersonal violence	C.3.2
4	Assault by firearm	C.3.2.1
4	Assault by sharp object	C.3.2.2
4	Assault by other means	C.3.2.4
3	Conflict and terrorism	C.3.3
3	Executions and police conflict	C.3.4

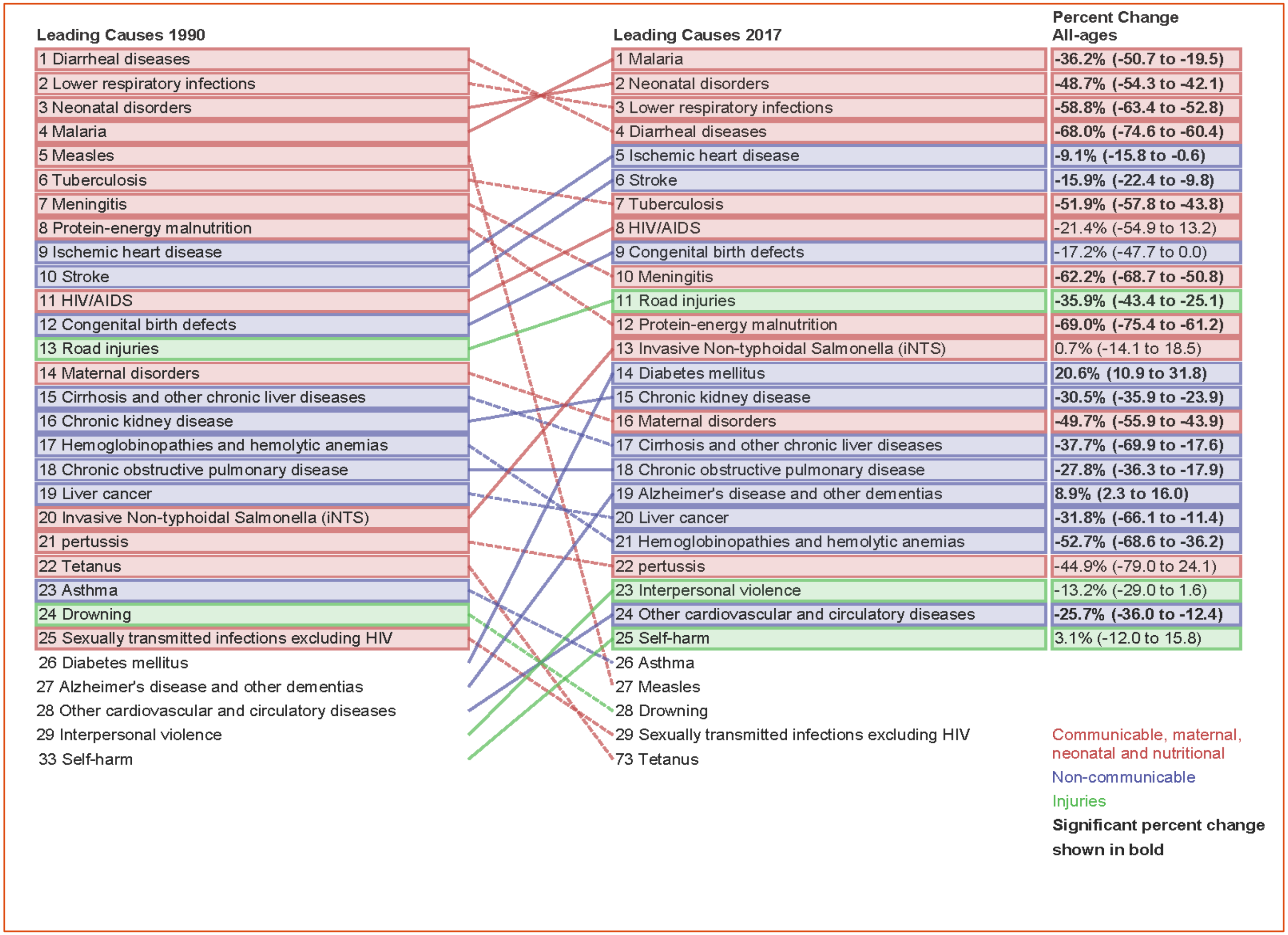

Figure 1: Leading causes of death and their evolution, all ages, Economic Community of West African States, francophone countries, 1990–2017

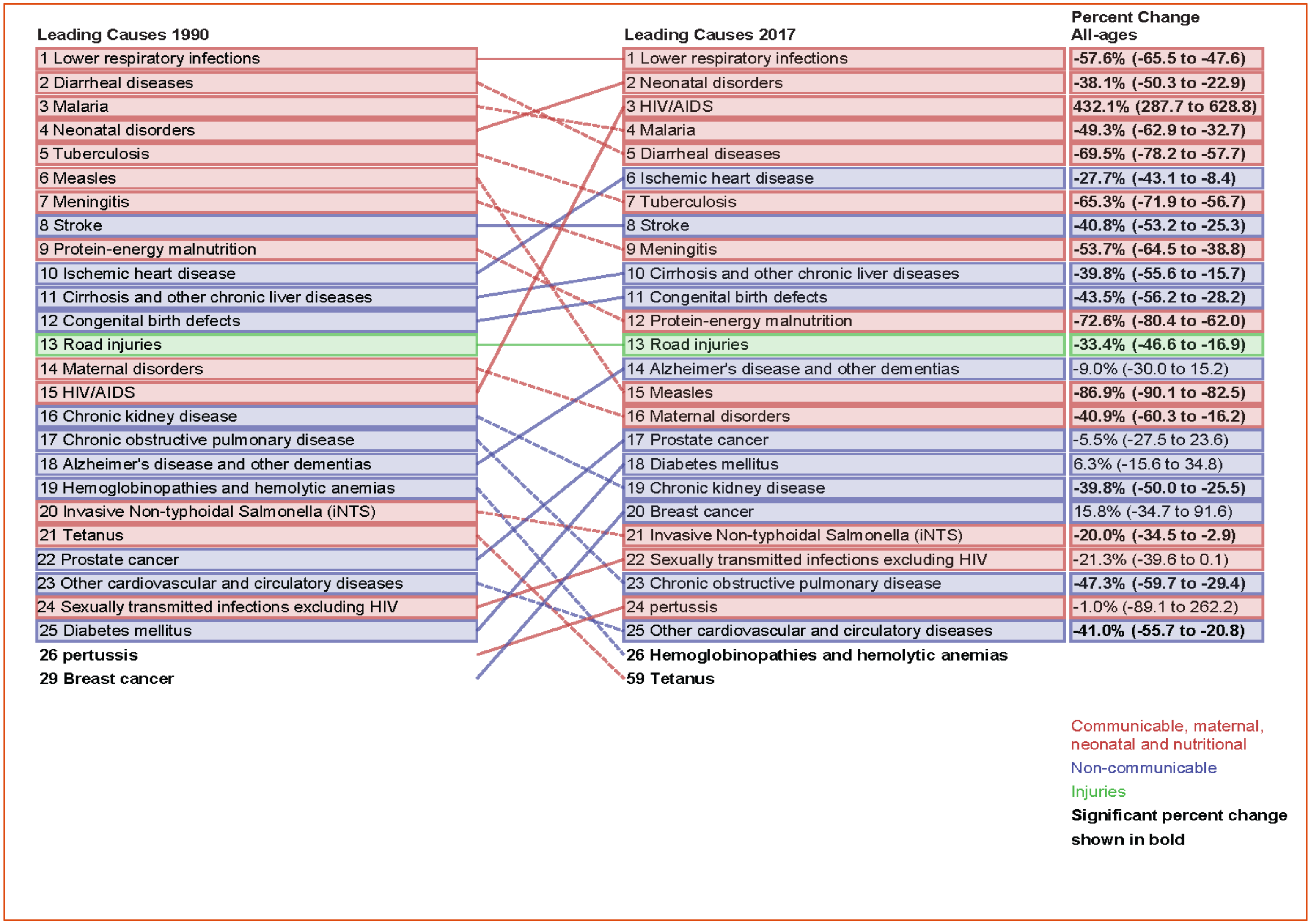

Figure 2: Leading causes of death and their evolution, all ages, Economic Community of West African States, non-francophone countries, 1990–2017

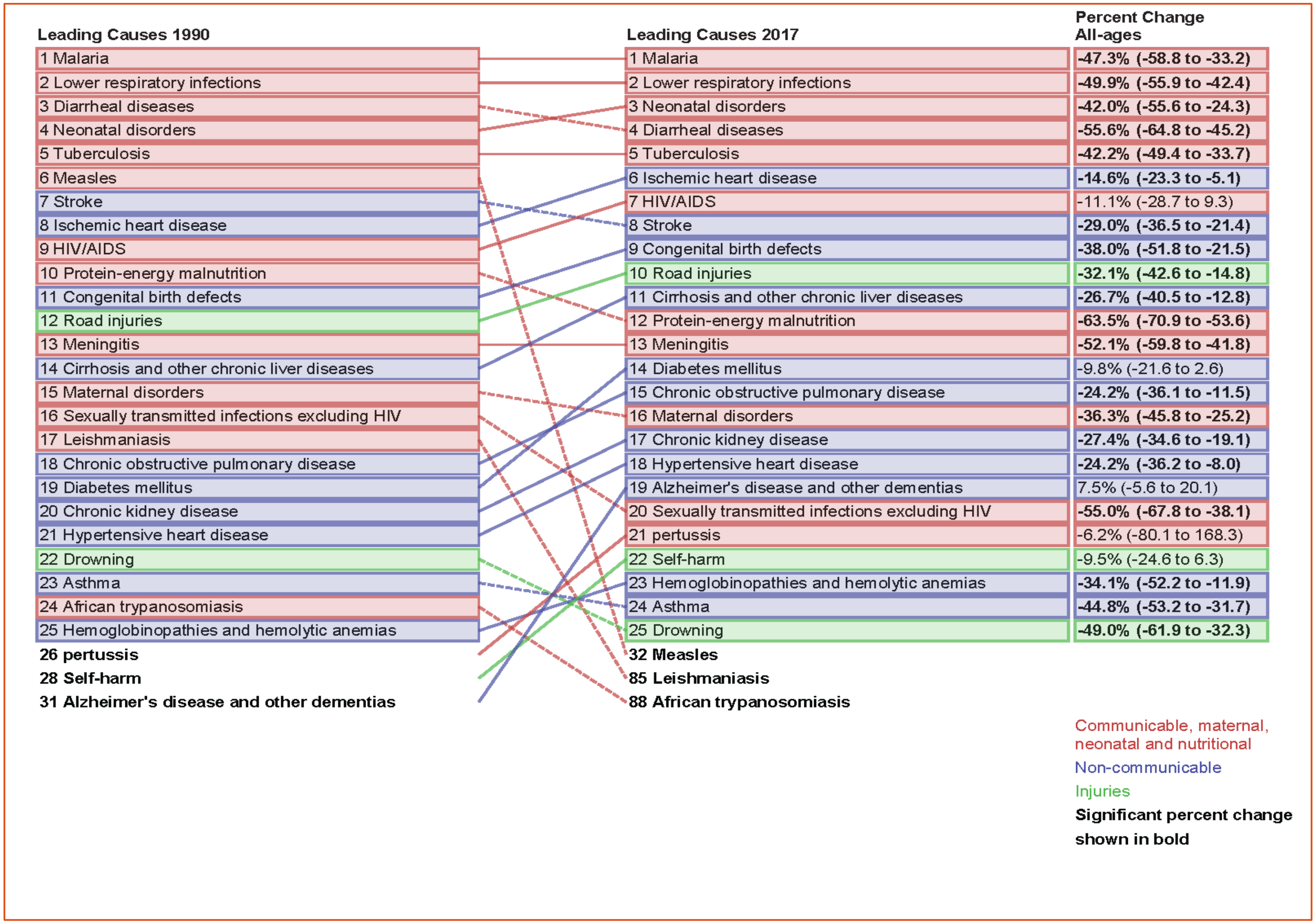

Figure 3**:** Leading causes of death and their evolution, all ages, Economic Community of Central African States, francophone countries, 1990–2017

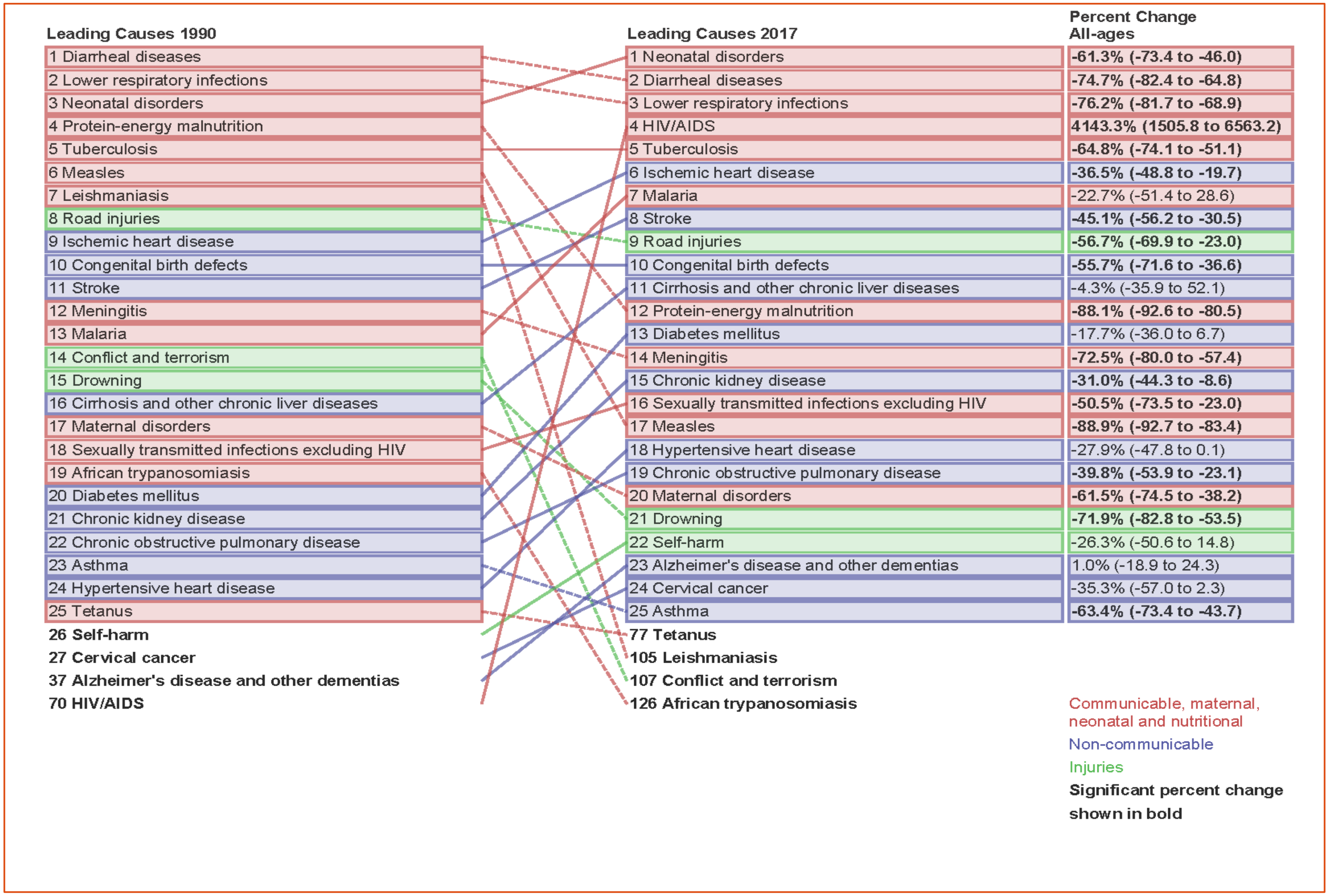

Figure 4: Leading causes of death and their evolution, all ages, Economic Community of Central African States, non-francophone countries, 1990–2017

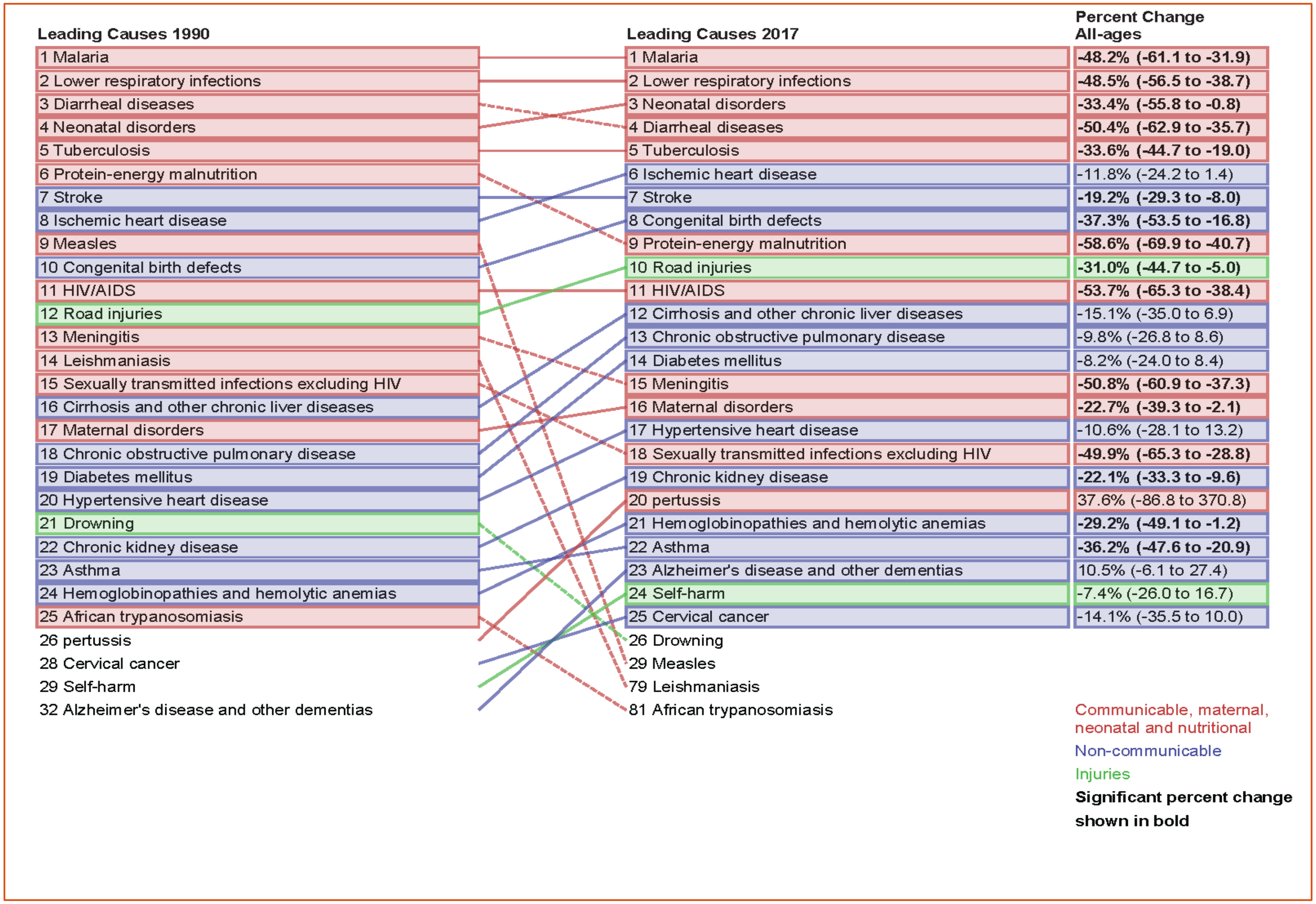

Figure 5: Leading causes of death and their evolution, all ages, Southern African Development Community, francophone countries, 1990–2017

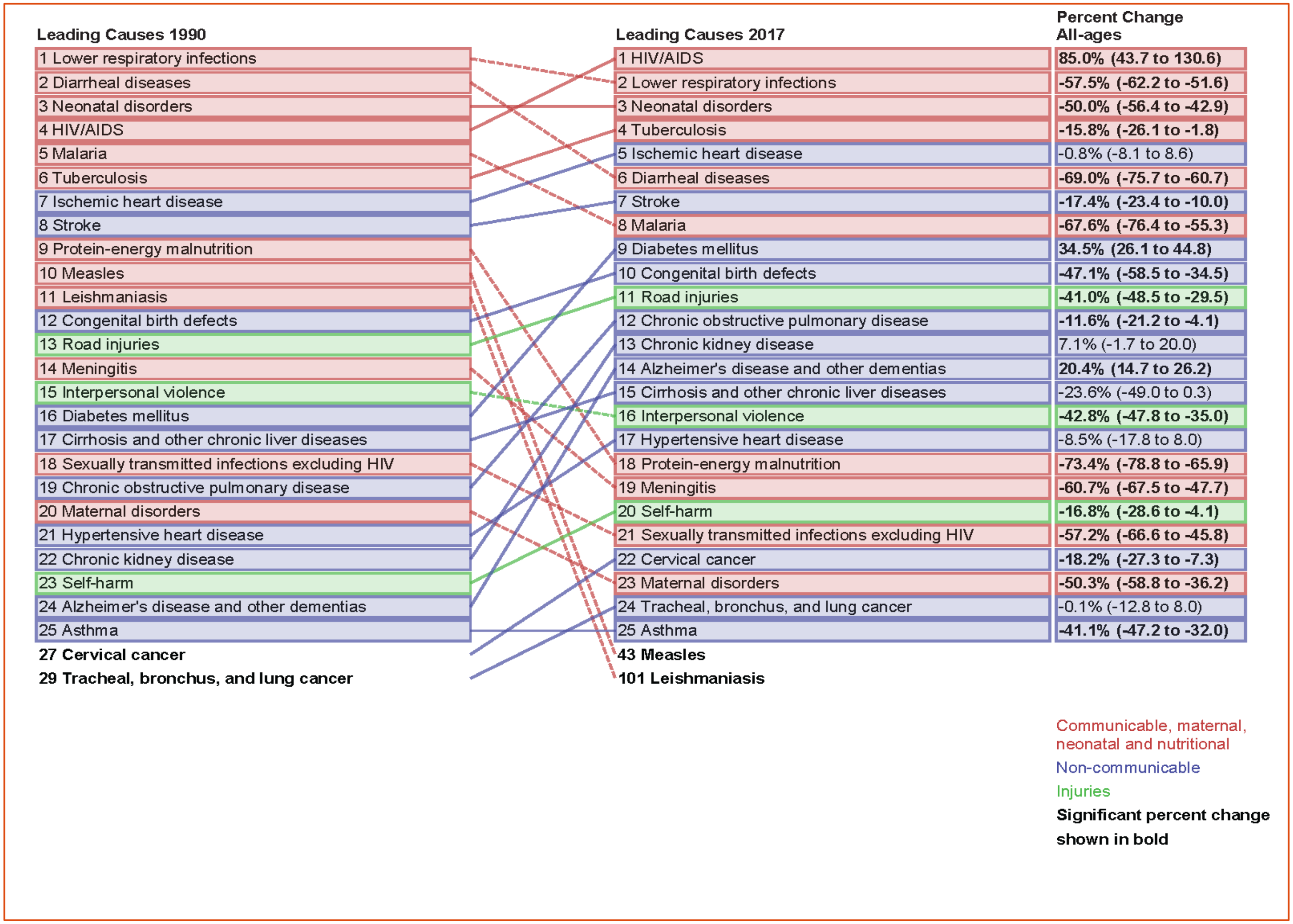

Figure 6**:** Leading causes of death and their evolution, all ages, Southern African Development Community, non-francophone countries, 1990–2017

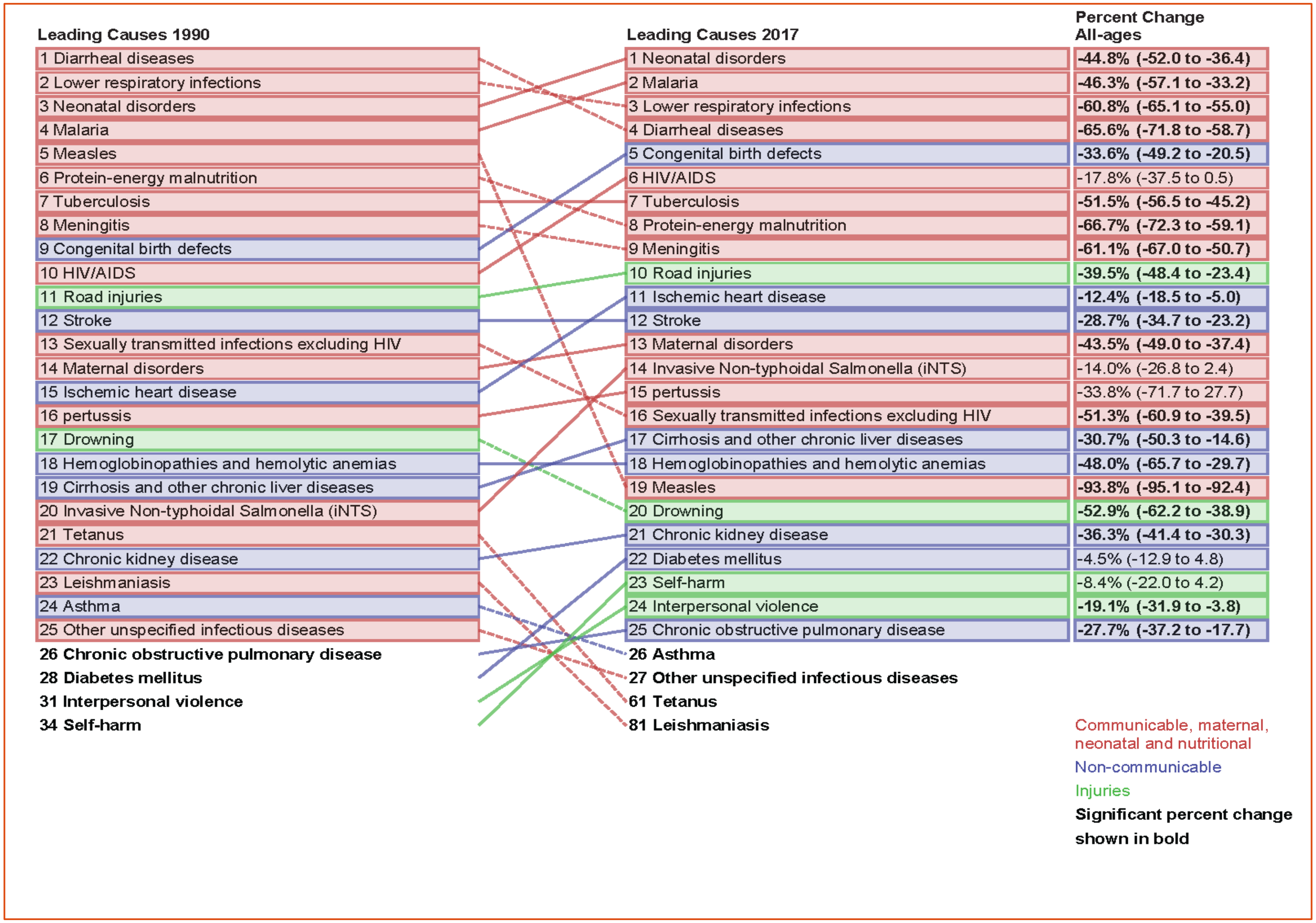

Figure 7**:** main causes of years of life lost and their evolution, all ages, francophone Africa, 1990–2017

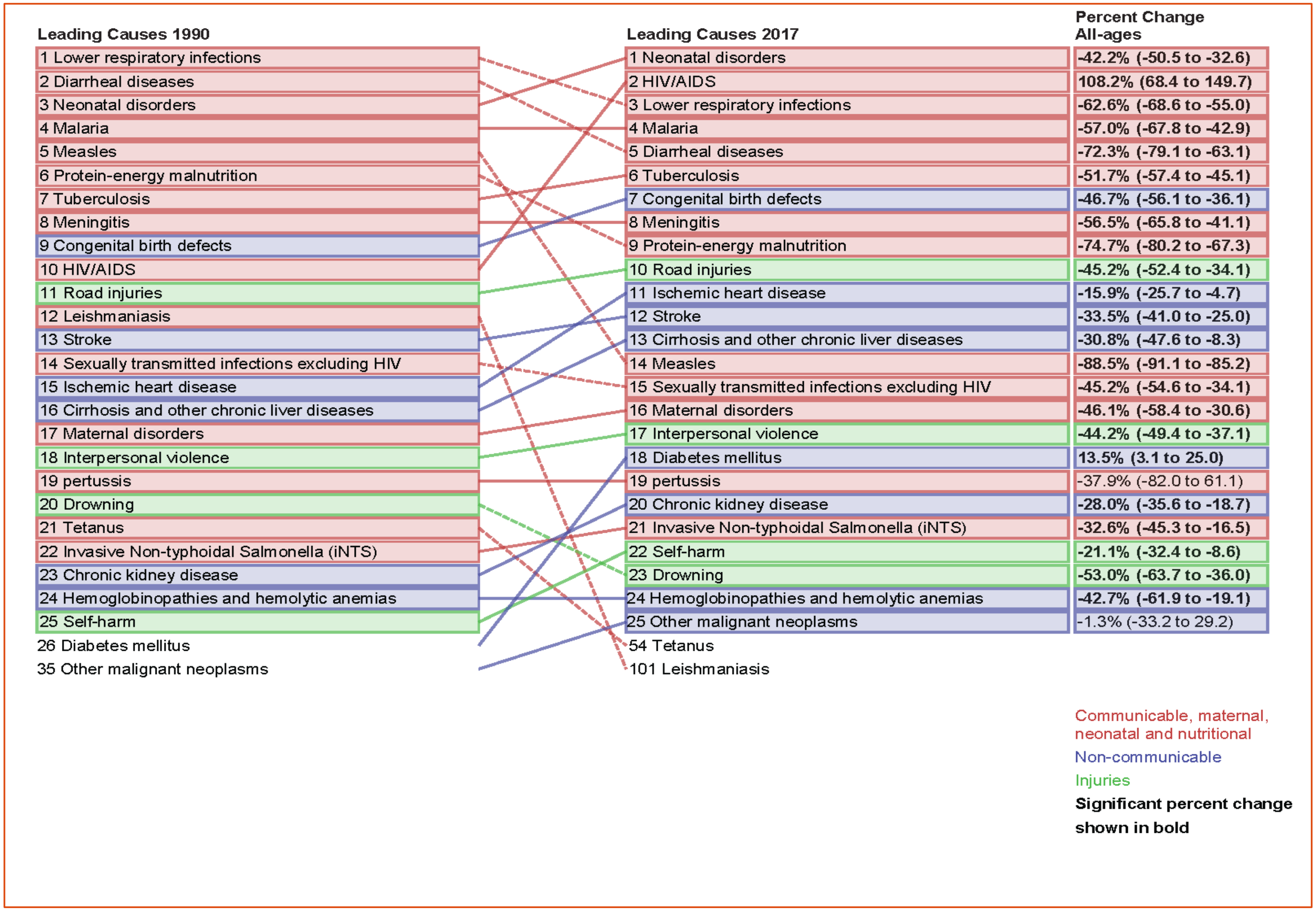

Figure 8: main causes of years of life lost and their evolution, all ages, non-francophone Africa, 1990–2017

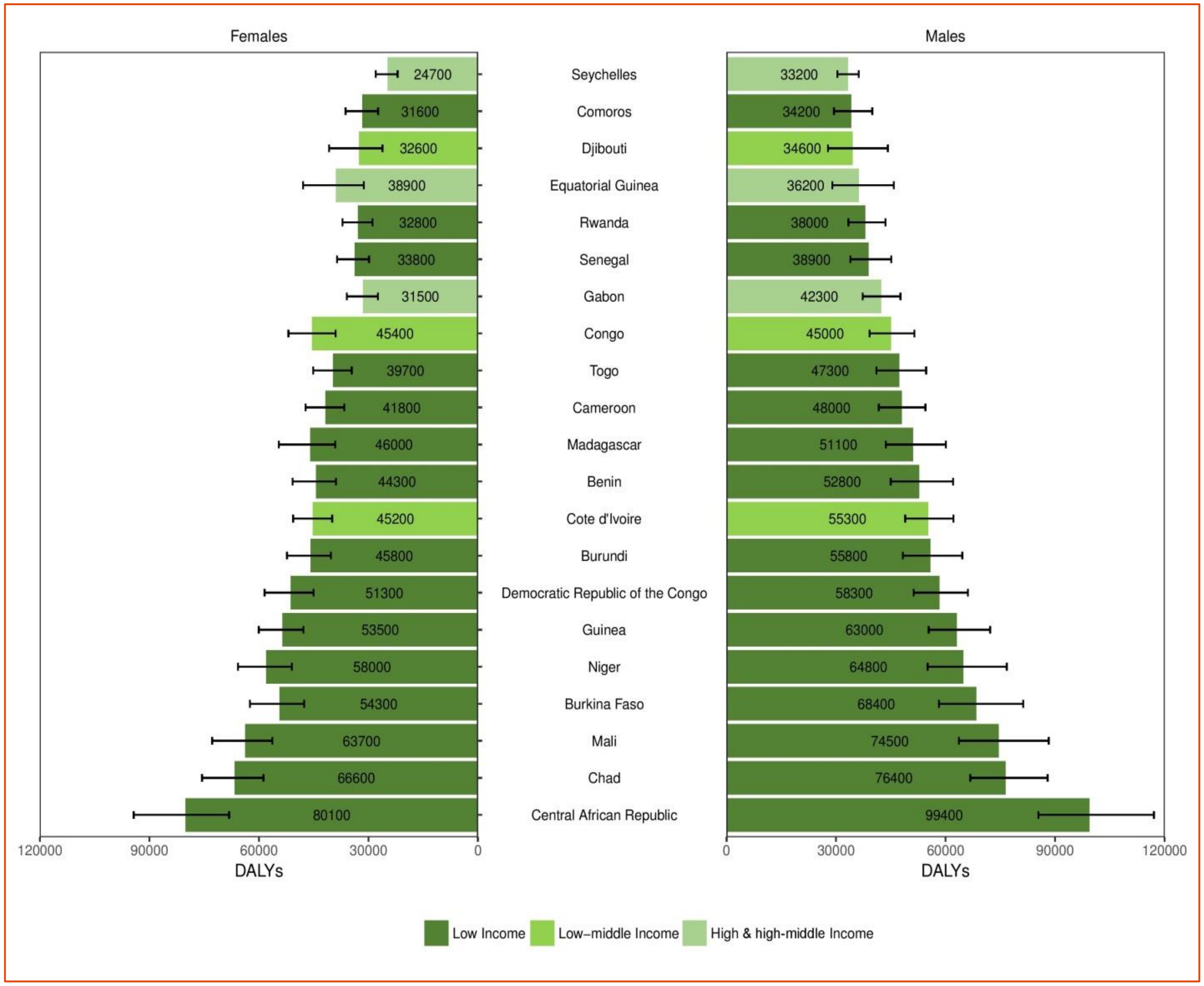

Figure 9**:** Disability-adjusted life-years (DALYs) per 100,000 population, and uncertainty intervals (black segments), all ages, by sex and country, francophone Africa, 2017

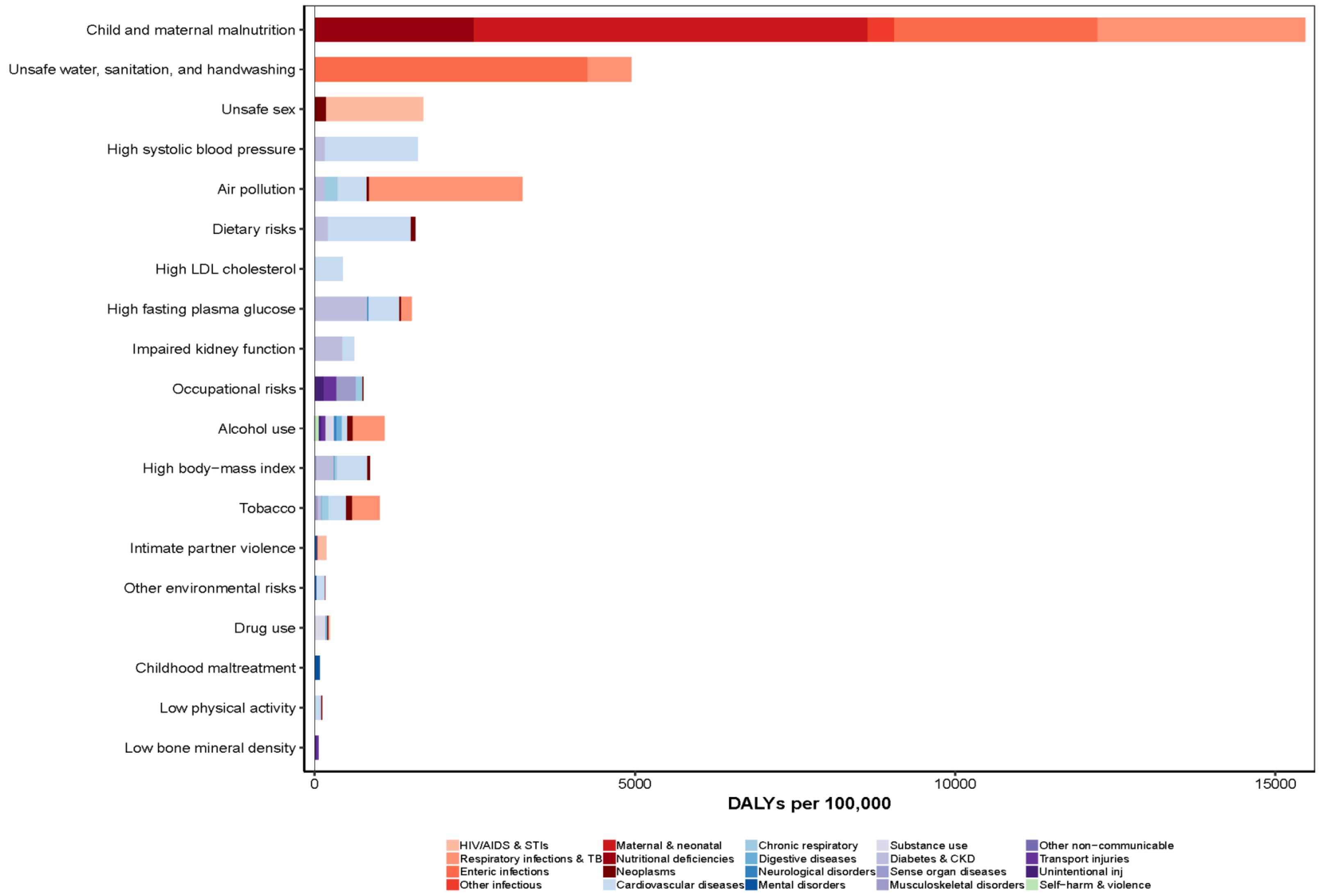

Figure 10: Risk factors by disability-adjusted life years, francophone Africa, 2017

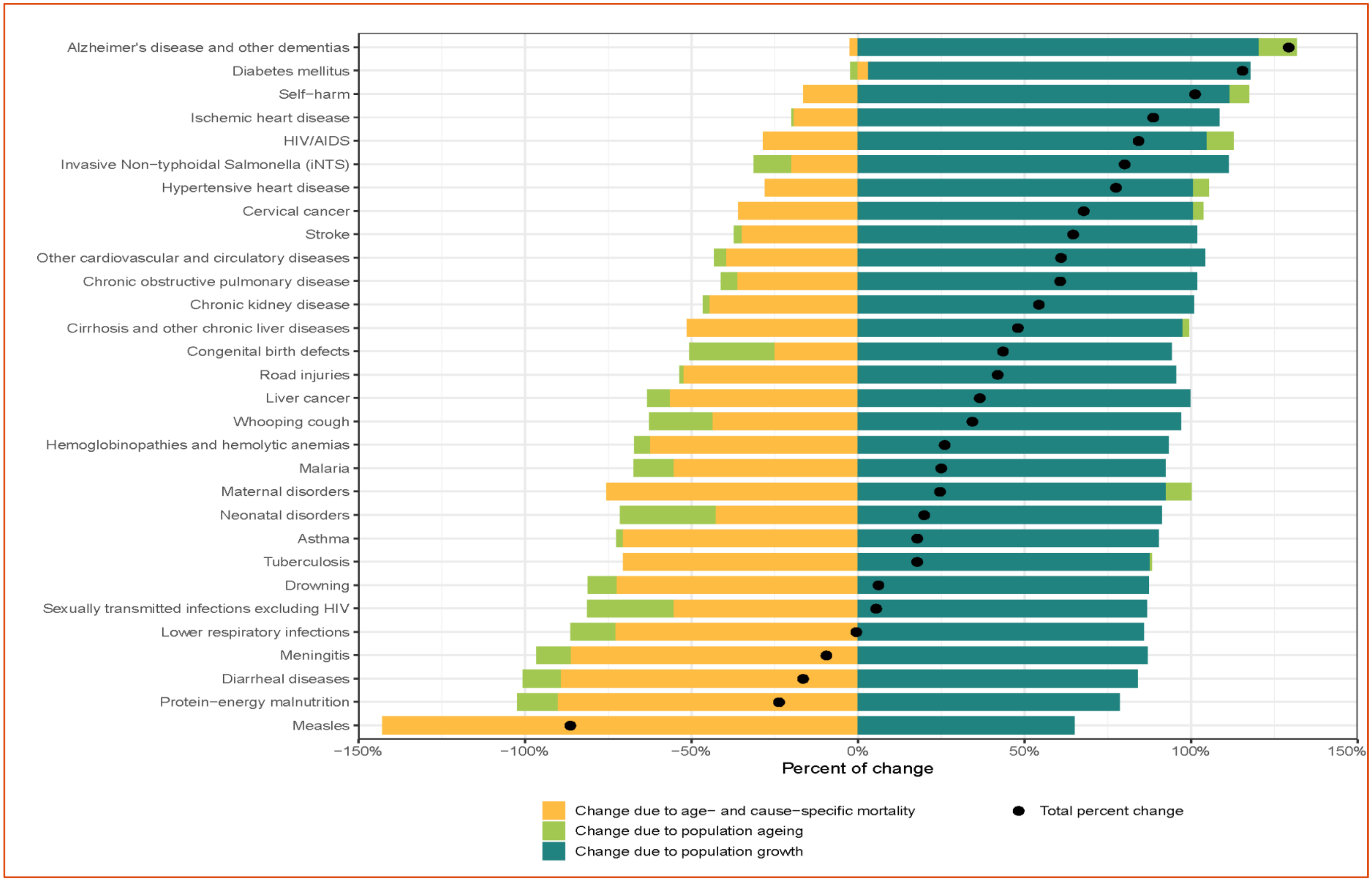

Figure 11**:** Decomposition of change among the 30 main causes of death due to population growth, population ageing, and age- and cause-specific mortality rate, francophone Africa, 1990–2017. For example, in the case on Alzheimer's disease and other dementias, age- and cause-specific mortality have contributed to a small decrease in DALYs, but population growth and population ageing drove a large increase making the total percent change about 130%.

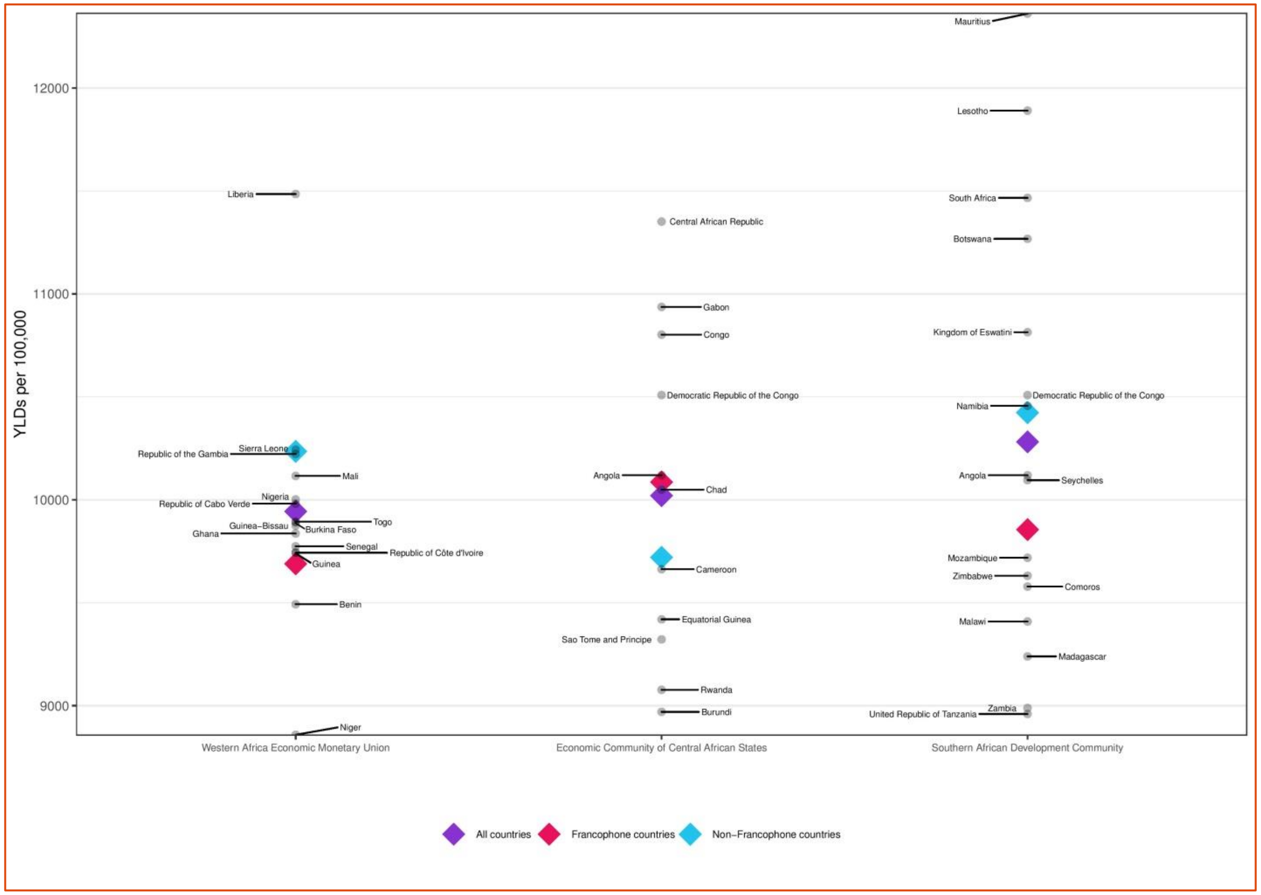

Figure 12**:** Years lived with disability (YLDs) in francophone (labeled to the right) and non-francophone (labelled to the left) countries, within the three economic communities

Table 3**:** Deaths, years of life lost (YLLs), years lived with disability (YLDs), and disability-adjusted life-years (DALYs) in 1990 and 2017, and life expectancy, expected DALYs, sociodemographic index (SDI) in 2017, and percent change in SDI between 1990 and 2017

**Location**	**1990**				**2017**								
	**Deaths (per 100,000)**	**YLLs (per 100,000)**	**YLDs (per 100,000)**	**DALYs (per 100,000)**	**Deaths (per 100,000)**	**YLLs (per 100,000)**	**YLDs (per 100,000)**	**DALYs (per 100,000)**	**Life Expectancy**	**Expected DALYs (per100,000)**	**Expected YLDs (per100,000)**	**SDI**	**Percent change in SDI from 1990-2017**
Benin (BEN)	1320 (1250 to 1390)	85400 (80400 to 91000)	11300 (8270 to 14800)	96600 (90400 to 103000)	697 (615 to 793)	39000 (33400 to 46300)	9490 (7040 to 12500)	48500 (42200 to 56200)	64.6 (62.7 to 66.5)	58257.08	9762.98	0.37	40.5%
Burkina Faso (BFA)	1590 (1510 to 1680)	104000 (98600 to 111000)	12000 (8800 to 15800)	117000 (110000 to 124000)	866 (770 to 986)	51300 (43500 to 61100)	9890 (7320 to 13000)	61100 (53000 to 71700)	61.7 (59.9 to 63.2)	84844.96	10197.89	0.28	53.6%
Burundi (BDI)	1580 (1480 to 1690)	97500 (91000 to 104000)	9190 (6780 to 11900)	107000 (99400 to 114000)	751 (670 to 843)	41800 (36300 to 48400)	8970 (6710 to 11600)	50800 (44600 to 58000)	61.6 (59.7 to 63.4)	76297.43	10050.75	0.31	38.7%
Cameroon (CMR)	1150 (1090 to 1210)	71200 (66900 to 75900)	11000 (8190 to 14400)	82100 (77100 to 87600)	689 (612 to 767)	35200 (30700 to 39800)	9660 (7210 to 12600)	44800 (39700 to 50600)	63 (61.1 to 64.9)	40458.35	9613.75	0.48	29.2%
Central African Republic (CAF)	1640 (1520 to 1760)	98100 (90500 to 107000)	11900 (8830 to 15500)	110000 (101000 to 120000)	1380 (1210 to 1570)	78300 (67200 to 92900)	11400 (8490 to 14700)	89700 (77900 to 105000)	51.9 (49.8 to 54.1)	68562.77	9925.1	0.33	24.2%
Chad (TCD)	1670 (1570 to 1770)	112000 (105000 to 119000)	11200 (8240 to 14600)	123000 (115000 to 131000)	963 (860 to 1080)	61400 (53700 to 71000)	10000 (7330 to 13100)	71500 (62900 to 81400)	60 (58.2 to 61.8)	95945.32	10401.52	0.25	28.0%
Comoros (COM)	1050 (966 to 1150)	62200 (56000 to 68100)	9800 (7170 to 12900)	72000 (65500 to 78600)	615 (549 to 683)	23300 (20200 to 27300)	9580 (7120 to 12400)	32900 (28900 to 37700)	68.6 (67.1 to 70.2)	46806.31	9620.36	0.43	37.2%
Congo (COG)	1240 (1140 to 1340)	65300 (60000 to 70800)	10900 (8170 to 14300)	76200 (70400 to 82400)	772 (686 to 872)	34400 (30100 to 39500)	10800 (8030 to 14000)	45200 (39800 to 51100)	62.7 (60.8 to 64.6)	32604.6	10003.77	0.57	33.3%
Cote d'Ivoire (CIV)	1150 (1060 to 1280)	75500 (69600 to 83000)	10600 (7750 to 13800)	86100 (79100 to 94800)	741 (663 to 819)	40600 (35900 to 46000)	9740 (7220 to 12800)	50400 (44900 to 56400)	62.5 (60.6 to 64.3)	51105.01	9663.38	0.41	31.7%

Democratic Republic of the Congo (COD)	1330 (1240 to 1410)	85600 (79700 to 91500)	11600 (8440 to 15100)	97200 (90300 to 104000)	795 (714 to 884)	44300 (38500 to 50700)	10500 (7810 to 13700)	54800 (48500 to 62000)	62.4 (60.7 to 64.1)	60612.28	9799.76	0.36	22.2%
Djibouti (DJI)	770 (657 to 896)	49600 (42600 to 57000)	8350 (6130 to 10900)	57900 (50700 to 65700)	559 (431 to 734)	24700 (18600 to 33300)	8940 (6650 to 11500)	33600 (27100 to 42700)	67.4 (64.1 to 70.2)	39860.25	9619.01	0.48	58.3%
Equatorial Guinea (GNQ)	1770 (1550 to 2010)	109000 (95200 to 125000)	11700 (8560 to 15500)	121000 (107000 to 137000)	540 (413 to 702)	28000 (21300 to 36900)	9420 (7070 to 12200)	37400 (30400 to 46700)	65.5 (62 to 68.8)	30856.14	10418.34	0.63	54.0%
Gabon (GAB)	1030 (961 to 1100)	47800 (44200 to 51500)	11300 (8280 to 14600)	59100 (54000 to 64000)	654 (593 to 716)	25800 (22800 to 28900)	10900 (8210 to 14100)	36700 (32500 to 41200)	68.4 (66.9 to 69.7)	30193.24	10627.78	0.65	29.2%
Guinea (GIN)	1800 (1700 to 1890)	119000 (110000 to 126000)	10700 (7870 to 13900)	129000 (121000 to 137000)	918 (834 to 1020)	48400 (42300 to 55900)	9740 (7220 to 12600)	58100 (51600 to 65700)	60.7 (59.1 to 62.3)	71532.98	9972.47	0.32	34.4%
Madagascar (MDG)	1250 (1190 to 1310)	77800 (73400 to 82700)	10200 (7470 to 13300)	87900 (82300 to 93800)	708 (617 to 816)	39300 (33100 to 47500)	9240 (6950 to 12100)	48500 (41600 to 57000)	63.5 (61.6 to 65.4)	70026.43	9948.33	0.33	24.2%
Mali (MLI)	1920 (1830 to 2010)	133000 (126000 to 141000)	11400 (8440 to 15200)	144000 (137000 to 153000)	920 (813 to 1050)	59000 (50500 to 69800)	10100 (7450 to 13300)	69100 (59900 to 80300)	62 (60.2 to 63.5)	92215.71	103059.81	0.27	48.1%
Niger (NER)	2080 (1960 to 2200)	156000 (146000 to 167000)	9990 (7340 to 13200)	166000 (156000 to 177000)	809 (709 to 929)	52500 (44900 to 62200)	8860 (6600 to 11600)	61400 (53200 to 71100)	62.4 (60.5 to 64.1)	123593.81	10934.33	0.19	26.3%
Rwanda (RWA)	1550 (1480 to 1620)	93600 (89100 to 98200)	9090 (6740 to 11800)	103000 (97500 to 108000)	543 (493 to 598)	26200 (23200 to 30200)	9080 (6770 to 11700)	35300 (31300 to 40000)	68.5 (67.1 to 69.9)	52028.61	9674.84	0.41	56.1%
Senegal (SEN)	1140 (1070 to 1200)	71000 (66100 to 75800)	10800 (7840 to 14200)	81700 (76200 to 87900)	596 (542 to 658)	26600 (23200 to 30900)	9770 (7280 to 12600)	36300 (32000 to 41700)	68 (66.7 to 69.3)	58257.08	9762.98	0.37	35.1%
Seychelles (SYC)	740 (724 to 758)	20600 (20000 to 21300)	8650 (6460 to 11100)	29300 (27000 to 31900)	756 (727 to 785)	19100 (18200 to 19900)	10100 (7560 to 13100)	29200 (26500 to 32100)	73.6 (73.1 to 74.1)	29076.79	10924.45	0.69	13.0%
Togo (TGO)	1060 (986 to 1140)	70000 (64600 to 75800)	10100 (7460 to 13200)	80100 (74200 to 86900)	671 (599 to 756)	33500 (29300 to 39100)	9890 (7320 to 12900)	43400 (38200 to 49300)	64.4 (62.6 to 66.2)	50212.42	9652.85	0.41	39.0%
Total	1423 (1395 to 1451)	92542 (90648 to 94501)	10887 (8034 to 14089)	103429 (100053 to 107349)	779 (750 to 809)	43708 (41673 to 45742)	9862 (7331 to 12749)	53570 (50164 to 57361)	64 (62.1 to 65.74)	67796.81	9963.50	0.35	31.4%

Table 4: cause of death star rating for vital registration systems, francophone Africa

**Location**	**Rating (0 – 5)**	**Stars**
Central African Republic	0	☆☆☆☆☆
Congo	0	☆☆☆☆☆
Democratic Republic of the Congo	1	★☆☆☆☆
Equatorial Guinea	0	☆☆☆☆☆
Gabon	0	☆☆☆☆☆
Burundi	1	★☆☆☆☆
Comoros	0	☆☆☆☆☆
Djibouti	0	☆☆☆☆☆
Madagascar	1	★☆☆☆☆
Rwanda	1	★☆☆☆☆
Seychelles	3	★★★☆☆
Benin	1	★☆☆☆☆
Burkina Faso	1	★☆☆☆☆
Cameroon	0	☆☆☆☆☆
Chad	0	☆☆☆☆☆
Cote d'Ivoire	1	★☆☆☆☆
Guinea	1	★☆☆☆☆
Mali	1	★☆☆☆☆
Niger	1	★☆☆☆☆
Senegal	1	★☆☆☆☆
Togo	0	☆☆☆☆☆
